# Community-based versus facility-based services to improve hepatitis C screening in Cambodia: a cluster randomized controlled trial (ANRS 12384 Cam-C study)

**DOI:** 10.1016/j.lanwpc.2025.101703

**Published:** 2025-10-08

**Authors:** Dyna Khuon, Luis Sagaon-Teyssier, Sansothy Neth, Saly Saint, Laurence Meyer, Emilie Mosnier, Diana Molino, Chan Leakhena Phoeung, Chhingsrean Chhay, Kimeang Heang, Sovatha Mam, Jean-Charles Duclos-Vallée, Olivier Ségéral, Vonthanak Saphonn

**Affiliations:** a University of Health Sciences, Phnom Penh, Cambodia; b Aix Marseille Univ, Inserm, IRD, SESSTIM, Sciences Economiques & Sociales de la Santé & Traitement de l'Information Médicale, ISSPAM, Marseille, France; c INSERM CESP U1018, Paris Saclay University, Le Kremlin-Bicêtre, Paris, France; d University of Health Sciences, ANRS Emerging Infectious Diseases (ANRS MIE) Partner Site, Phnom Penh, Cambodia; e National Institute of Health and Medical Research (Inserm), ANRS Emerging Infectious Diseases (ANRS MIE), Paris, France; f Fédération Hospitalo-Universitaire Hépatinov, Inserm UMR-1193, Paul Brousse Hospital, AP-HP, Paris-Saclay University, Villejuif, France; g HIV Unit, Infectious Diseases Department, Geneva University Hospital, Geneva, Switzerland

**Keywords:** Hepatitis C, HCV, Rapid diagnostic test uptake, Point-of-care, Cluster randomized trial, Community-based strategy, Active HCV infection detection, Cambodia

## Abstract

**Background:**

The estimated prevalence of hepatitis C virus (HCV) infection among adults aged over 45 years in Cambodia is approximately 5%. The present study aimed assessing the effectiveness of a novel community-based strategy comprising HCV rapid testing (RDT) and dried blood spot (DBS) collection performed by community health workers (CHWs) in outreach contexts.

**Methods:**

ANRS 12384 Cam-C is a parallel arm cluster randomized controlled trial conducted in the Siem Reap and Kampong Cham provinces of Cambodia between April 2022 and September 2023. Four geographical areas in each province were randomized into two arms to compare HCV RDT and DBS collection provided in homes by community health workers (i.e., community-based strategy) with facility-based HCV RDT and whole blood collection provided by healthcare workers in health centers (i.e., facility-based strategy). The primary outcome was combined testing uptake, defined as the proportion of participants who were actually tested for HCV using RDT or RDT/RNA if positive out of all those who participated in the study. Mixed effects logistic models were used to estimate the effect of the community-based strategy. The study is registered on ClinicalTrials.gov (NCT03992313).

**Findings:**

Among the 7692 participants who initially enrolled in the study, 5590 (72.7%) actually went on to have HCV RDT or HCV RDT/RNA screening (i.e., combined testing uptake). Combined testing uptake in the community-based (2990 participants) and facility-based (2600 participants) arms was 78.1% and 67.3%, respectively (p < 0.001). Positive HCV RDT was found for 57 (2.2%) including 39 (1.5%) with detectable HCV RNA among participants in the facility-based arm. In the community-based arm, 37 (1.2%) had positive HCV RDT including 32 (1.1%) with detectable HCV RNA. Treatment uptake concerned 36 participants (out of 39 with positive HCV RDT) and 30 (out of 32) in the facility-based and community-based arms, respectively. The odds of combined testing uptake in the community-based strategy were 2.18 (95% CI [1.28–3.73], p = 0.004) times higher than in the facility-based strategy.

**Interpretation:**

Combined testing uptake (HCV RDT or HCV RDT/RNA) was higher for the community-based strategy than for the facility-based strategy. The involvement of community health workers improved first contact with HCV services and uptake. Integrating this trial's community-based screening strategy into the national Cambodian HCV program could improve its effectiveness as part of the country's broader goal of eliminating HCV by 2030.

**Funding:**

This study was supported by the 10.13039/501100003323ANRS MIE (ANRS 12384).

**Research in context**

**Evidence before this study**

Hepatitis C virus (HCV) infection prevalence in Cambodia is estimated at between 1.1% and 5.2%, with substantial variations across the country's different regions. The country's HCV epidemic is characterized by high geographic clustering, a concentration among adults over 40 years, and limited access to HCV-related health services—primarily diagnosis and treatment—especially in rural areas. Prevalence is also higher in people living with HIV compared to the general population. All these factors create major challenges for Cambodia to reach its goal of eliminating HCV by 2030. This is especially true for access to diagnosis and treatment. In 2022, an estimated 23% of people living with HCV were aware of their status, and only 12% of them received treatment.

In 2018, the Cambodian Ministry of Health and the non-governmental organization Médecins Sans Frontières implemented a pilot HCV screening and treatment program in the operational district (i.e., the most geographically peripheral sub-unit in the country's health system) of Moung Russei, located in Battambang province. The program demonstrated the importance of simplifying and decentralizing HCV care in order to improve access to testing, retention in care and consequently, treatment effectiveness. However, as the program used a facility-based strategy for HCV testing and treatment, it is very probable that people furthest from the healthcare system were not reached. These people tend to come from the most vulnerable populations. In order to reach them, innovative strategies tailored to resource-limited settings are required which provide HCV services outside of health facilities (i.e., outreach). Involving community health workers in such strategies—especially by demedicalizing HCV screening—is crucial to increase accessibility. We searched for studies conducted in low- and middle-income countries between 2016 and 2024 on PubMed using the following search syntax: (“HCV” or “hepatitis C” or “Hep C”) and (“community” or “community-based” or “participatory” or “decentralized” or “primary care”) and (“screening” or “testing” or “treatment”). In the relevant studies we identified, the community-based nature of the strategies implemented principally concerned activities conducted by health professionals in communities, sometimes with community health workers providing counseling for prevention and support focused on retention in treatment. We also searched for studies conducted within the framework of trials (randomized or not) for strategies aimed at improving HCV screening and treatment. We used the following search syntax: (“HCV” or “hepatitis C” or “Hep C”) and (“screening” or “testing” or “treatment”) and (“trial”). Although the various interventions we identified in relevant studies proved to be effective in terms of screening, linkage to care, and retention in care, all corresponded to high-income country contexts.

**Added value of this study**

Our parallel arm cluster randomized controlled trial demonstrated that further simplification and effectiveness of HCV care in Cambodia is possible by training community health workers in HCV infection screening. The community-based demedicalized strategy used in the trial was more effective in increasing accessibility to HCV rapid testing and uptake in vulnerable populations (specifically, women, older persons, those living alone, and inactive people) than the facility-based strategy. Our results suggest that this strategy could help to overcome structural barriers by widening the ‘gateway’ to HCV care, and could reduce gender and socioeconomic inequalities in access to HCV services. We also found that once people became aware of their HCV positive status, linkage to care, access to treatment and retention did not depend on the strategy used (i.e., facility-based versus community-based); this demonstrates that the community-based strategy specifically contributes to improved screening and diagnosis. Finally, our results provide evidence that the community-based strategy we used in Cambodia could be exported to other countries with similar HCV epidemic, economic and health system organization profiles.

**Implications of all the available evidence**

The community-based (i.e., demedicalized) HCV screening strategy tested in this cluster randomized controlled trial can be added to existing tools to improve access to HCV screening and care in Cambodia, as part of the country's wider plan to eliminate HCV by 2030. It can be replicated in other regions and integrated into existing health systems, as no additional human or material resources are needed. Furthermore, this strategy helps to overcome the challenges of simplifying the HCV care model raised in previous studies. A consortium comprising local health authorities and researchers is currently planning to incorporate this demedicalized screening strategy into the planned Cambodian National Strategic program for Viral Hepatitis C infection control. As part of this process, we shall conduct implementation research to maximize its potential.

## Introduction

In 2022, 7.1 million people were living with hepatitis C virus (HCV) in the World Health Organization's (WHO) Western Pacific region. Of these, 55% were unaware of their status and only 16% of total infections (including those cured) were treated.^1^ HCV in this region represents approximately 14% of the global burden, and 10% of the world's 980,000 new infections. These figures highlight the fact that one of the main ongoing weaknesses of current HCV programs in the region is insufficient screening and diagnosis.

The last decade has seen an increasing number of studies exploring strategies to improve HCV screening and linkage to care,^2^^,^^3^ including same-day testing and treatment,^4^ simplification of care pathways, and the decentralization of care using rapid diagnostic tests (RDT) in primary care settings.^5^^,^^6^ The success of programs in the broader context of the WHO's goal of eliminating HCV by 2030, depends on the capacity of health systems to strengthen primary care and to adapting services to specific population needs.^1^^,^^7^^,^^8^ In this regard, community-based strategies, where community health workers (CHWs) are directly involved in providing care, are a key element for improving the flexibility, simplification and adaptation of screening and diagnosis services, and, as a consequence, increasing acceptability of HCV services, especially by vulnerable populations.^9^, ^10^, ^11^ Our literature search highlighted that the role of CHWs is often limited to only providing counseling and support for HCV treatment adherence.^12^, ^13^, ^14^, ^15^ Training CHWs in relatively simple medical activities, including demedicalized (i.e., not performed by a healthcare professional) rapid HCV testing and dried blood spot collection, could ensure that strategies to provide HIV health services to more people are implemented more effectively. Moreover, thanks to their close relationship with these populations, besides simply giving support for treatment adherence, CHWs could also increase awareness about risk factors, and provide advice to reduce HCV-related stigma.^8^^,^^16^

In Cambodia, 77% of the population living with HCV infection are unaware of their status and only 12% of total infections are treated. These figures are much higher than the average values for the Western Pacific region (55% and 16%, respectively).^1^ In the relatively few HCV studies conducted in Cambodia to date, the estimated prevalence ranged from 1.1% to 5.2% across all of the country's 25 provinces. This variability has also been documented at the intra-provincial level (Moun Russei district) with prevalence among adults ranging from 0.74% to 4.6%.^17^ Variability is much higher in specific populations; for instance, HCV prevalence in people living with HIV (PLHIV) varies from 1.3% in Siem Reap province, to 12% in Preah Sihanouk province.^18^^,^^19^ Furthermore, all the above-mentioned studies found that infection was concentrated among adults over 40 years of age, the estimated prevalence in this population ranging from 3.6% to 9.5%. Besides this high geographic and age clustering of cases, the HCV epidemic in Cambodia is mostly iatrogenic, and driven by limited access to quality healthcare, especially in rural areas.^17^^,^^20^ To respond to this situation, Cambodian authorities launched an ambitious national HCV elimination strategy in 2016. Although testing is a central feature of this program, its uptake is still limited, especially among rural and socio-culturally marginalized populations.^17^ Involving CHWs could be a key element in such strategies, especially in contexts where hyperendemic areas need to be identified to ensure the effectiveness of the new national program.^4^^,^^21^^,^^22^

In this context, the present study aimed comparing the effectiveness of two strategies focused on improving HCV RDT uptake or HCV RDT/RNA uptake in adults aged 40 and over: the existing facility-based strategy, which comprises RDT and whole blood collection performed by healthcare workers in healthcare structures, and a novel community-based strategy, which comprises HCV RDT and dried blood spot collection performed by CHWs in outreach contexts. In order to take into account both the organization of Cambodia's health system and the community-based nature of the intervention, we used a cluster randomized design.

## Methods

### Design overview

ANRS 12384 Cam-C is a parallel arm cluster randomized controlled trial conducted in Siem Reap and Kampong Cham provinces in Cambodia between April 2022 and September 2023. Four geographical areas in each province (i.e., eight in total) were randomly assigned to one of the study's two HCV RDT strategies (i.e., study arms). In the **facility-based strategy**, CHWs provided residents in the four geographical areas with information about HCV testing; HCV RDT testing was subsequently performed by healthcare workers in local health facilities. For positive HCV RDT results, healthcare workers then took whole blood samples to be sent for confirmatory HCV RNA testing. In the **community-based strategy**, just as was the case for the other strategy, CHWs provided residents with information about HCV testing; they also conducted HCV RDT testing in people's homes. For positive HCV RDT results, the collected dried blood spots (DBS) to be sent for confirmatory HCV RNA testing. The study was designed and reported in accordance with CONSORT guidelines and the extension to cluster randomized trials.^23^ A detailed description of the design can be found elsewhere.^24^

### Settings and participants

The three-tiered health system in Cambodia is organized into provincial health departments (PHD) which each have one provincial hospital. Each PHD covers from one to ten operational districts (OD), each of which has at least one referral hospital. Finally, health centers (HC), which constitute the lowest level of the healthcare system, are located in OD and cover between ten and twenty thousand people.

**Fig. 1:**
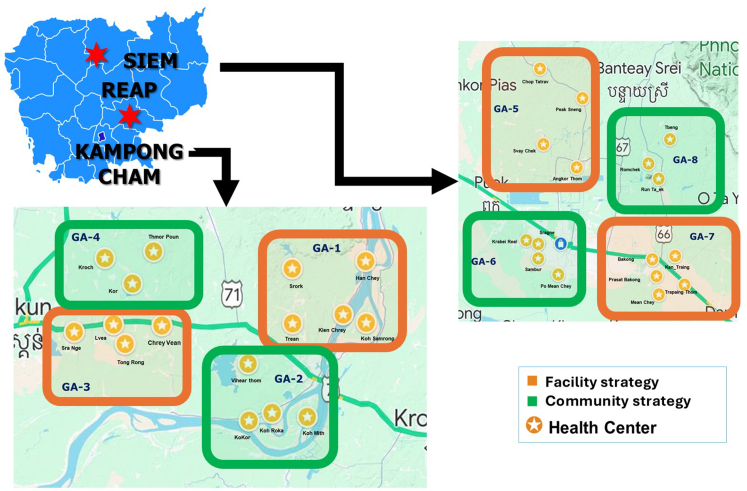
**Cambodia: provinces and geographical areas concerned according to the study's two strategies**.

Siem Reap and Kampong Cham provinces are approximately 140 km from each other. Both have a similar population of approximately one million habitants. Accordingly, we can say that they are similar in terms of healthcare service supply. For the purposes of our study, four geographical areas (GA) per province were defined and randomly assigned to either the facility-based (Arm 1) or community-based (Arm 2) strategies (i.e., four GA per strategy). GA did not overlap in order to avoid information contamination (Fig. 1).

In Siem Reap province, the four GA were located in one OD, whereas the four GA in Kampong Cham province were located in two OD. The three ODs were comparable in terms of the urban/rural structure and equidistant from their respective provincial hospital. This ensured having the same number of villages and approximately the same population in each GA. All adults over 40 years old who resided in the eight GA, and who provided consent to participate in the CAM-C study were eligible. The study itself comprised a quantitative survey administered by trained research assistants which all participants answered. It collected information about demographic, socioeconomic and household characteristics, perceived health status as well as history of HCV testing and treatment. After participants completed this survey, CHWs invited them to test for HCV either at home (community-based strategy) or in a nearby HC (facility-based strategy) depending on which GA they lived in. People with known positive HCV status, those who had previously been treated for HCV, those with a severe life-threatening disease, and those currently participating in another clinical study were all excluded from participation. Recruitment started in April 2022 and the study including data collection ended in September 2023.

### The intervention

The study intervention consisted in comparing two HCV testing strategies—called the ‘facility-based and community-based strategy—which targeted adults aged 40 years and over (i.e., the population with the greatest concentration of HCV infection). For both strategies, CHWs visited all the households in the 160 clusters covered by the study's eight GA. They identified eligible participants, provided information about the study, invited people to participate and obtained written consent for the collection of data concerning demographic, socioeconomic, health- and household-related information.

In the facility-based strategy, the CHWs who provided information (through information notices and leaflets) about HCV screening were not trained in RDT testing. After the trained research assistants administered the quantitative survey, CHWs invited participants to test for HCV; they provided those who agreed with a free-test voucher for a HCV RDT test to use in their closest HC. Health professionals in HC conducted the testing. Results were provided after 15 min; people with a negative HCV RDT were provided with counseling about how to avoid transmission. People with a positive HCV RDT who agreed to have HCV RNA testing immediately provided a complete blood sample (5 mL in an EDTA tube) which was sent to the relevant provincial hospital for plasma extraction. The plasma was then sent to the Pasteur Institute of Cambodia in the capital Phnom Penh for HCV RNA laboratory testing.

Unlike the facility-based strategy, the CHWs who provided information about HCV testing in the community-based strategy, were specifically trained in HCV RDT testing and dried blood spot (DBS) collection. They invited participants who completed the quantitative survey to screen for HCV at home. When this was not possible—for example because of socio-cultural, time-related or other structural barriers—an appointment for a later date was set up to have the test in a pre-designated premises specifically chosen by the head of the relevant village for the CAM-C study. More generally, the heads of villages facilitated the organization and planning of HCV RDT screening by CHWs in these premises. Participants who missed their first appointment were given a second one. No additional opportunities were organized for participants who missed both appointments. DBS samples were sent to the Rodolph Mérieux laboratory in Phnom Penh for HCV RNA extraction and amplification.

### Clinical treatment and follow-up

General practitioners consulted participants diagnosed with active HCV infection in the corresponding provincial hospital. When necessary, a hepatologist provided support for clinical examinations, blood sampling and liver ultrasounds. Patients presenting ascites, collateral circulation around the umbilicus, encephalopathy, jaundice, or hepatocellular carcinoma were referred to the hepatology unit in the Calmette National Hospital in Phnom Penh. All other patients were offered treatment with DAA after excluding those with at least one of the following conditions: an indication for palliative care, HCC diffuse with infiltration, hepato-renal syndrome type 1, severe renal insufficiency (creatinine clearance <30mL/mn), pregnancy, breastfeeding, contraindication for sofosbuvir and/or daclatasvir, allergic reactions to active substances and/or to excipients, any condition compromising the safety of the patient (at the judgment of the physician). DAA treatment consisted in a combination of daily sofosbuvir 400 mg + daclatasvir 60 mg (WHO pre-qualified generic drugs) for 12 weeks. Eligible patients started treatment immediately after the first consultation in the provincial hospital. Patients were offered monthly follow-up consultations. Consultations at weeks 4, 8, and 12 included the assessment of potential adverse events and the delivery of DAA treatment for the subsequent month. DAA adherence was also evaluated at each consultation using a questionnaire and a visual analog scale.

### Completion of treatment

Blood samples were collected to assess sustained viral response (SVR12) at the 24-week consultation. We assessed the Child score for cirrhotic patients and screened for HCC using ultrasound. Patients were informed they were cured at week 28 if their blood sample at 24 weeks indicated SVR12. A liver ultrasound was recommended every six months for cirrhotic patients. Patients without SVR12 were referred to the National Hospital in Phnom Penh where a multidisciplinary team decided on therapeutic options.

### Study outcomes

The study's *primary outcome* was HCV RDT or HCV RDT/RNA uptake (combined testing uptake), defined as the proportion of participants who were actually tested for HCV using RDT or RDT/RNA if positive out of all those who participated in the study.

*Secondary outcomes* included: i) confirmation testing uptake, defined as the proportion of persons testing positive after an RDT who also had laboratory HCV RNA testing, among the total number of persons who accepted HCV RDT; ii) active HCV infection detection proportion, defined as the proportion of persons with active HCV infection (i.e., a positive HCV RDT and a detectable HCV RNA test) among the total number of persons who initially accepted HCV RDT; iii) linkage to care, defined as the proportion of people with active HCV infection who subsequently consulted for treatment and care among the total number of persons with active HCV infection; and v) treatment initiation, as the proportion of people initiating DAA treatment among those who consulted for treatment and care (i.e., linked to care). In addition, we have considered an additional exploratory outcome concerning the confirmation testing uptake specific to positive RDT, defined as the proportion of persons who had laboratory HCV RNA testing, among the total number of people testing positive to RDT.

We hypothesized that the offer of HCV RDT by CHWs in people's homes (or dedicated premises that were not a HC) and the collection and storage of DBS by CHWs for those who tested positive would result in significant differences between screening uptake between the community-based and facility-based strategies. We also hypothesized that there would be no differences between the two strategies for active case detection or linkage to care, as healthcare and support activities related to these steps in the cascade of care depended exclusively on structural factors (e.g., organization of health centers, laboratories, increased workload, etc.) and individuals' care seeking behaviors.

### Sample size

The sample size calculation was based on the hypothesis that a higher proportion of participants in the community-based strategy, specifically 80%, would agree to get screened than participants in the facility-based strategy, specifically 60%. We also took into account the following factors: i) demographic information indicating that the mean number of four persons in rural households, including 1 person aged 40 years or more,^25^ leading to equal cluster sizes of 50 households per cluster; and ii) HCV epidemiological information indicating a seroprevalence of approximately 5% among people aged 45 years or more.^17^ Furthermore, to calculate the sample size, we used the following statistical assumptions: an intra-class correlation (ICC) of 0.01, a significance level of 5%, a power of 80%, and a design effect of 1.5 to account for the extent to which the expected sampling error was due to cluster design compared to simple random sampling. Combining these assumptions, we calculated that the study required 300 people aged 40 years or more testing positive using HCV RDT (i.e., 150 per strategy). In order to reproduce a global seroprevalence of 5%, 6000 households (i.e., 3000 per strategy) needed to be visited to ensure that at least one person per household aged 40 years or more would accept HCV RDT. This translated into an expected number of 6000 people accepting HCV RDT. On the assumption that 75% of the participants in the study would actually go through with HCV RDT (i.e., a total of 25% either not using their free-test voucher at an HC (facility-based strategy) or missing both scheduled appointments in a predesignated premises (community-based strategy)), we calculated that 8000 households would be needed to be visited to reach at least one person aged 40 year or more to be enrolled in the study. This translated into a required sample size of 4000 people per strategy, in a total of 160 clusters, or 80 clusters of 50 people per strategy.

### Randomization

Participants were randomized into the two study arms (i.e., strategies) in the eight GA (see above), stratified according to the province, four in Siem Reap province and four in Kampong Cham province, with 1:1 randomized allocation. We used multi-stage cluster sampling to select study participants. In each GA, 10 villages were randomly selected in urban and rural areas. This random selection together with the comparable structure between the operational districts of the study in terms of urban/rural population for the study ensured that there was no bias introduced. We then visited each village to evaluate the number of households per village, and the geographical location of each household; the head of each village provided information about inhabitants' ages. Two or more neighboring small villages (<100 households) were combined within the same GA in order to form a group (namely “village”) with at least 100 households. From this we constructed a list to select 100 households per village using simple random sampling.

A group of 50 households defined a cluster; accordingly, there were two clusters per village, 20 per GA, 80 per province, for a total of 160 clusters. In each household visited, all persons aged 40 years or more were invited to participate in ANRS 12384 Cam-C. Separate written consent for different elements of the study was collected: i) consent to participate in the cross-sectional quantitative survey (which was separate from consent to have HCV RDT screening); ii) consent for HCV RDT screening; iii) for participants with a positive HCV RDT, consent for subsequent HCV RNA testing; iv) for participants testing positive the HCV RDT and with detectable HCV RNA test, consent for treatment if. A research assistant administered the cross-sectional quantitative questionnaires face to face using a tablet to collect demographic, socioeconomic, health data and household-related information. Participants who agreed to have HCV RDT screening, those who had a positive HCV RDT result, those who agreed to subsequent HCV RNA testing, and those who had a confirmed diagnosis and agreed to start treatment answered additional questions in the questionnaire. Clinical information was recorded using electronic case report forms (eCRF).

### Statistical methods

We described the baseline characteristics of participants and compared them in both strategies using the standardized mean/proportion difference as a measure indicating the magnitude of the difference between the two strategies (i.e., effect size).^26^ Given that one of the main limitations of this measure is that there is no consensus on the threshold to determine the significant difference, especially in presence of large heterogeneous samples,^27^ we adopted the expanded Cohen's rules with 0.01 indicating at least a very small difference between strategies.^28^ The primary outcome (i.e., combined testing uptake) was presented as a percentage with its corresponding 95% confidence interval, and was described for each strategy. Secondary outcomes (i.e., confirmation testing uptake; active HCV infection detection, linkage to care, and on DAA treatment) were also presented as percentages with their corresponding 95% confidence intervals.

We estimated mixed effects logistic models to account for data nested at different levels (i.e., multilevel models): individuals (level 1), clusters of households (level 2), and villages (level 3). In these models, intercept random effects were specified for clusters of households and villages in order to account for the different sources of variability in the outcomes. The pertinence of the multilevel structure was tested using the LR-test statistic (H0: $\sigma_{cluster}^{2}=\sigma_{village}^{2}=0$, i.e., no difference across clusters or across villages. Intent-to-treat and per protocol estimations were conducted for the primary outcome by specifying fixed effects for both strategies (community-based; facility-based. Then, all per protocol estimations included: age (continuous, testing its quadratic effect) and province (Siem Reap; Kampong Cham) as confounding variables pre-planned in the protocol. In addition, multi-level models also included the following variables for adjustment as they presented differences at baseline: gender (women; men), marital status (single; married-cohabiting; widowed-divorced-separated), activity workload (no activity; working ≤7 day/week and <8 h/day; working 7 day/week and ≥8 h/day); household members previously diagnosed with and treated for HCV (yes; no); lifetime HCV testing for the participant (yes; no); participant-perceived health status at the time of the interview (very poor/poor/moderate; good/very good). For the secondary outcomes only the adjusted effect for each strategy is shown. Adjusted odd-ratios (OR), 95% confidence intervals (95% CI) and p-values are presented for fixed effects, whereas estimated coefficients (Coeff.) and 95% CI are presented for intercepting random effects for clusters and villages, as well as ICC with 95% CI. Sensitivity analysis was conducted using the inverse probability weighting (IPW) technique in order to correct the differences between the facility-based and the community-based arms in terms of the abovementioned characteristics (i.e., reducing the bias between arms).^29^ The weighted mixed effects logistic models were estimated for the primary and secondary outcomes in order to verify whether the uneven distribution of characteristics between arms resulted in different conclusions or not (Supplementary File 1).

All statistical analyses were carried out using Stata 17.^30^

### Ethics approval

ANRS 12384 Cam-C is registered on ClinicalTrials.gov (NCT03992313). The protocol was approved by the National Ethics Committee for Human Research (NECHR) in Cambodia on February 25, 2019 (N°047). It conforms with the 2013 updated version of the Declaration of Helsinki, and with the French Public Health Code. All participants provided written informed consent.

### Role of the funding source

The funder of this study had no role in study design, data collection, data analysis, data interpretation or writing of the article.

## Results

**Fig. 2:**
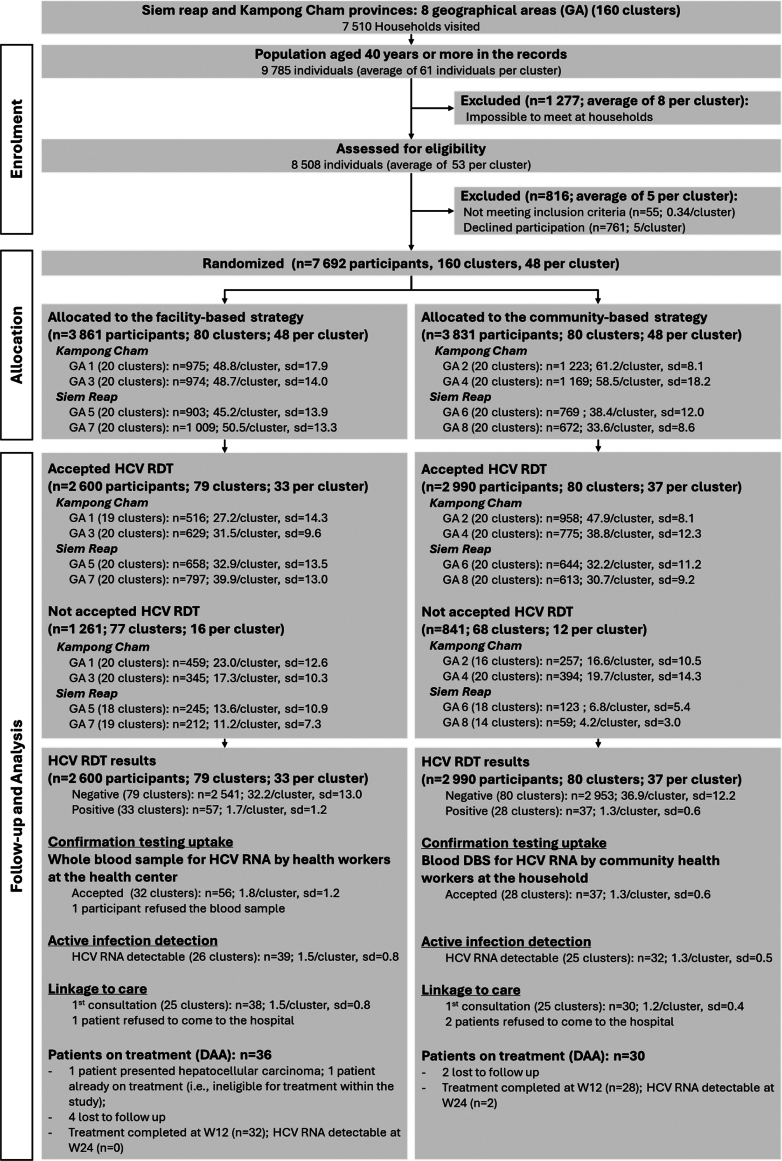
**ANRS 12384 Cam-C study—flow diagram of progress of clusters and individuals**.

Fig. 2 provides the CONSORT (Consolidated Standards of Reporting Trials and the extension to cluster randomized trials, Supplementary File 2) study flow diagram of the study. Between April and August 2022, we visited a total of 160 clusters (i.e., 80 per arm), each comprising a mean of 47 households, for a total of 7510 households and 9785 people aged 40 years or more. Among the latter, 8508 were assessed for eligibility for the study. Of these, 7692 participated, representing 96% of the 8000 required. All of them answered at least the cross-sectional quantitative survey, and subsequently either agreed or refused to be screened at a HC by healthcare workers (facility-based strategy) or at home/predesignated village premises by CHWs (community-based strategy).

### Sample characteristics

Table 1 summarizes the characteristics of the 7692 participants; 3861 (56.4%) and 3831 (54.6%) were enrolled in Kampong Cham and Siem Reap provinces, respectively. In the community-based strategy, participants were older (median [IQR] age 56 [48–66] years versus 55 [46–64] years in the facility-based strategy), there was a slightly higher proportion of females (58.7% versus 56.8%), a lower proportion of married/cohabitating participants (75.9% versus 78.5%), and a higher proportion of participants with no activity (68.4% versus 58.4%). Given the standardized mean/proportion differences observed in all the factors in Table 1, they were included as covariates in the mixed effects model which investigated the effect of the community-based strategy on the probability of combined testing uptake with respect to the facility-based strategy.

**Table 1:** Baseline characteristics according to strategy (i.e., study arm) at individual and cluster levels (N = 7692).

Characteristics	Arm 1: facility-based (n = 3861)	Arm 2: community-based (n = 3831)	Standardized difference^a^	Total
**Individual level**	**% (n) or median [IQR]**	**% (n) or median [IQR]**		**% (n) or median [IQR]**

**Age (years)**	55 [46–64]	56 [48–66]	0.153	55 (47–65)
**Gender**				
Men	1669 (43.2)	1583 (41.3)	0.039	3252 (42.3)
Women	2192 (56.8)	2248 (58.7)		4440 (57.7)
**Marital status**				
Single	90 (2.3)	128 (3.3)	0.076	218 (2.8)
Married/cohabitating	3030 (78.5)	2906 (75.9)		5936 (77.2)
Widowed/divorced/separated	741 (19.2)	797 (20.8)		1538 (20.0)
**Activity workload**				
No activity	2253 (58.4)	2621 (68.4)	0.235	4874 (63.4)
Work ≤7 days per week and <8 h per day	699 (18.1)	632 (16.5)		4331 (17.3)
Work 7 days per week and ≥ 8 h per day	909 (23.5)	578 (15.1)		1487 (19.3)
**Other household member(s) previously treated for HCV**^b^				
Yes	62 (1.6)	41 (1.1)	0.047	103 (1.3)
No	3775 (97.8)	3783 (98.8)		7558 (98.7)
**Pre-study history of HCV testing (participant)**^c^				
Yes	130 (3.4)	119 (3.1)	0.016	249 (3.3)
No	3706 (95.9)	3705 (96.7)		7411 (96.8)
**Perceived health status at the time of the interview**^d^				
Good/very good	2346 (61.0)	2568 (67.2)	0.129	4914 (64.1)
Very poor/poor/moderate	1499 (39.0)	1253 (32.8)		2752 (35.9)
**Province**				
Kampong Cham	1949 (50.5)	2392 (62.4)	0.243	4341 (56.4)
Siem Reap	1912 (49.5)	1439 (43.6)		3351 (43.6)
**Cluster level**	**No. clusters (mean; sd)**	**No. clusters (mean; sd)**	**No. clusters (mean; sd)**	

**Age groups**				
<50	80 (16.0; 6.6)	80 (13.9; 5.7)	160 (14.9; 6.3)	
50–59	80 (14.6; 6.5)	80 (14.5; 6.6)	160 (14.6; 6.5)	
60–69	80 (11.2; 4.9)	80 (11.0; 4.9)	160 (11.1; 4.9)	
70+	78 (6.5; 4.0)	79 (8.5; 5.5)	160 (7.4; 5.0)	
**Gender**				
Men	80 (20.9; 6.9)	80 (19.8; 8.8)	160 (20.3; 7.5)	
Women	80 (27.4; 8.6)	80 (28.1; 9.9)	160 (27.6; 9.2)	
**Marital status**				
Single	41 (2.2; 1.6)	53 (2.4; 1.8)	94 (2.3; 7.7)	
Married/cohabitating	80 (37.9; 11.8)	80 (36.2; 14.9)	160 (37.1; 13.4)	
Widowed/divorced/separated	80 (9.3; 4.8)	80 (10.0; 4.4)	160 (9.6; 4.6)	
**Activity workload**				
No activity	80 (28.2; 13.4)	80 (32.8; 15.6)	160 (30.5; 14.7)	
Work ≤7 days per week and <8 h per day	72 (9.7; 8.0)	62 (10.2; 11.1)	134 (9.9; 9.5)	
Work 7 days per week and ≥ 8 h per day	78 (11.7; 8.9)	73 (7.9; 7.2)	151 (9.8; 8.3)	
**Other household member(s) previously treated for HCV**				
Yes	14 (4.4: 5.3)	20 (2.1; 1.4)	34 (3.0; 3.7)	
No	160 (23.6; 14.3)	160 (23.6; 16.7)	160 (47.2; 15.5)	
**Pre-study history of HCV testing (participant)**				
Yes	36 (3.6; 4.7)	33 (3.6; 3.9)	69 (3.6; 4.4)	
No	80 (46.3; 14.4)	80 (46.3; 16.1)	160 (46.3; 15.2)	
**Perceived health status at the time of the interview**				
Good/very good	77 (30.5; 17.6)	78 (32.9; 20.5)	155 (31.7; 19.1)	
Very poor/poor/moderate	78 (19.2; 16.9)	75 (16.7; 13.8)	153 (18.0; 15.4)	
**Province**				
Kampong Cham	40 (48.7; 15.9)	40 (59.8; 14.0)	80 (54.2; 13.6)	
Siem Reap	40 (47.8; 13.7)	40 (36.0; 10.6)	80 (41.9; 13.6)	

a Standardized difference: differences in means or proportions divided by standard error, with imbalance defined by an effect size according to the following values: 0.01 (very small), 0.2 (small), 0.5 (medium), 0.8 (large), 1.2 (very large), and 2 (huge).

b 31 missing values.

c 32 missing values.

d 26 missing values.

### Primary outcome

Of the 7692 participants enrolled in the study, after completing the quantitative questionnaire, 5590 actually had an HCV RDT or HCV RDT/RNA if positive (overall uptake proportion of 72.7% 95% CI [71.7%–73.7%]). Uptake was higher in the community-based strategy, with 78.1% 95% CI [76.7%–79.4%] of enrolled participants (n = 2990) being tested, compared to 67.3% 95% CI [65.9%–68.8%] in the facility-based strategy (n = 2600) (p < 0.001). Table 2 shows that, except for gender or marital status, there were important differences between the two strategies. Overall, 94 participants tested positive for HCV: 57 in the facility-based strategy and 37 in the community-based strategy.

**Table 2:** Characteristics according to study strategy (i.e., study arm) at individual and cluster level for participants who had an HCV RDT (N = 5590).

Characteristics	Facility-based (n = 2600)	Community-based (n = 2990)	Standardized difference^a^	Total
**Individual level**	**% (n) or median [IQR]**	**% (n) or median [IQR]**		**% (n) or median [IQR]**

**Age (years)**	55 [46–64]	56 [48–65]	−0.146	56 [47–65]
**Gender**				
Men	1065 (41.0)	1187 (39.7)	0.026	2252 (40.3)
Women	1535 (59.0)	1803 (60.3)		3338 (59.7)
**Marital status**				
Single	65 (2.5)	83 (2.8)	0.044	148 (2.7)
Married/cohabitating	2018 (77.6)	2265 (75.7)		4283 (76.6)
Widowed/divorced/separated	517 (19.9)	642 (21.5)		1159 (20.7)
**Activity workload**				
No activity	1724 (66.3)	2158 (72.2)	0.137	3882 (69.5)
Work ≤7 days per week and <8 h per day	397 (15.3)	416 (13.9)		813 (14.5)
Work 7 days per week and ≥ 8 h per day	479 (18.4)	416 (13.9)		895 (16.0)
**Other household member(s) previously treated for HCV**				
Yes	4 (0.2)	15 (0.5)	0.061	19 (0.3)
No	2596 (99.8)	2975 (99.5)		5571 (99.7)
**Pre-study history of HCV testing (participant)**				
Yes	19 (0.7)	39 (1.3)	0.057	58 (1.0)
No	2581 (99.3)	2951 (98.7)		5532 (99.0)
**Perceived health status at the time of the interview**				
Good/very good	1667 (64.1)	2033 (68.0)	0.082	3700 (66.2)
Very poor/poor/moderate	933 (35.9)	957 (32.0)		1890 (33.8)
**Province**				
Kampong Cham	1145 (44.0)	1733 (58.0)	0.281	2878 (51.5)
Siem Reap	1455 (56.0)	1257 (42.0)		2712 (48.5)
**Cluster level**	**No. clusters (mean; sd)**	**No. clusters (mean; sd)**	**No. clusters (mean; sd)**	

**Age groups**				
<50	79 (10.8; 6.6)	80 (11.1; 4.7)	159 (11.0; 5.7)	
50–59	79 (10.1; 5.4)	80 (11.2; 4.3)	159 (10.7; 4.9)	
60–69	78 (8.1; 4.1)	79 (9.1; 4.0)	157 (8.6; 4.1)	
70+	74 (4.2; 2.8)	77 (6.3; 4.1)	151 (5.3; 3.6)	
**Gender**				
Men	79 (13.5; 6.1)	80 (14.8; 5.4)	159 (14.2; 5.8)	
Women	78 (19.7; 7.5)	80 (22.5; 7.6)	158 (21.1; 7.7)	
**Marital status**				
Single	33 (2.0; 1.2)	42 (2.0; 1.4)	75 (2.0; 1.3)	
Married/cohabitating	79 (24.5; 10.7)	80 (28.3; 10.3)	159 (26.9; 10.6)	
Widowed/divorced/separated	78 (6.6; 3.7)	79 (8.1; 4.0)	157 (7.4; 3.9)	
**Activity workload**				
No activity	74 (23.3; 11.7)	80 (27.0; 14.3)	154 (25.2; 13.2)	
Work ≤7 days per week and <8 h per day	54 (7.4; 8.1)	48 (8.7; 7.8)	102 (8.0; 7.9)	
Work 7 days per week and ≥ 8 h per day	64 (7.5; 8.4)	61 (6.8; 6.6)	125 (7.2; 7.6)	
**Other household member(s) previously treated for HCV**				
Yes	3 (1.3; 0.6)	9 (1.7; 1.3)	12 (1.6; 1.2)	
No	79 (32.9; 13.3)	80 (37.2; 12.1)	159 (35.0; 12.8)	
**Pre-study history of HCV testing (participant)**				
Yes	15 (1.3; 0.6)	14 (2.8; 4.3)	29 (2.0; 3.0)	
No	79 (32.7; 13.3)	80 (36.9; 11.9)	159 (34.8; 12.8)	
**Perceived health status at the time of the interview**				
Good/very good	64 (26.0; 14.0)	72 (28.2; 16.2)	136 (27.2; 15.2)	
Very poor/poor/moderate	58 (16.1; 18.2)	58 (16.5; 13.3)	116 (16.3; 16.0)	
**Province**				
Kampong Cham	39 (29.4; 12.1)	40 (43.3; 11.3)	79 (36.4; 13.6)	
Siem Reap	40 (36.4; 13.5)	40 (31.4; 10.1)	80 (33.9; 12.3)	

a Standardized difference: differences in means or proportions divided by standard error, with imbalance defined by an effect size according to the following values: 0.01 (very small), 0.2 (small), 0.5 (medium), 0.8 (large), 1.2 (very large), and 2 (huge).

Table 3 presents the mixed effects models for HCV RDT uptake for intention-to-treat and per protocol estimations. The LR-test comparing the logistic three-level null model (Models 0 and 1) with a single-level logistic model, indicates significant variability of the outcome due to differences between villages (**σ^2^_villages_**: 0.59, 95% CI [0.39–0.92], **σ^2^_villages_**: 1.35, 95% CI [0.91–1.99], respectively) and clusters (**σ^2^_clusters_**: 0.29, 95% CI [0.18–0.45], **σ^2^_clusters_**: 0.18, 95% CI [0.10–0.33], respectively). The LR-statistics for Model 0 (LR = 1106) and for Model 1 (LR = 1065.99) were higher than >5.991 (i.e., the χ^2^ (2) theoretical value at 5% significance level) indicating the pertinence of the mixed effects model. The unadjusted odds-ratio (OR) and the 95% CI in the intention-to-treat estimation (Model 0) indicate that the probability of HCV RDT uptake would be 1.91 times higher in the community-based arm compared with the facility-based arm (OR: 1.91, 95% CI [1.29–2.83], p = 0.001). This result remained consistent in the per protocol estimation (Model 1) where the odds of combined testing uptake were 2.44 times higher in the community-based arm (OR: 2.44, 95% CI [1.41–4.23], p < 0.001).

**Table 3:** Mixed effects model to assess the effect of the community-based strategy (i.e., study arm) with respect to the facility-based strategy (i.e., study arm) on combined testing uptake (Intention-to-treat and per protocol estimations).

Fixed-effects	Intention-to-treat (N = 9785)			Per protocol (N = 7692)^a^					
	Model 0			Model 1			Model 2		
	OR	[95% CI]	p-value	OR	[95% CI]	p-value	aOR	[95% CI]	p-value
**Arm**									
Community-based (ref. facility-based)	1.91	[1.29–2.83]	0.001	2.44	[1.41–4.23]	<0.001	2.18	[1.28–3.73]	0.004
**Age** (continuous)							1.11	[1.05–1.16]	<0.001
**Age^2^** (non-linear effect)							0.999	[0.998–0.9994]	<0.001
**Gender**: female (ref. male)							1.16	[1.02–1.32]	0.024
**Marital status** (ref. single)									
Married/cohabitation							2.01	[1.43–2.84]	<0.001
Widowed/divorced/separated							1.97	[1.36–2.84]	<0.001
**Activity workload** (ref. no activity)									
Work ≤7 d/7 and <8 h/d							0.33	[0.28–0.40]	<0.001
Work 7 d/7 and ≥8 h/d							0.25	[0.21–0.30]	<0.001
**Members in the household ever diagnosed and treated for HCV**									
Yes (ref. no)							0.19	[0.10–0.37]	<0.001
**Ever tested for HCV**									
Yes (ref. No)							0.13	[0.09–0.20]	<0.001
**Perceived health status at the time of the interview**									
Moderate/bad/very bad (ref. very good/good)							0.39	[0.33–0.45]	<0.001
**Province**: Seam Reap (ref. Kampong Cham)							3.12	[1.82–5.34]	<0.001
**Random-effects: 80 villages/160 clusters**	**Coeff.**	**95% CI**		**Coeff.**	**95% CI**		**Coeff.**	**95% CI**	

**σ^2^_clusters_**	0.29	[0.18–0.45]		0.18	[0.10–0.33]		0.23	[0.13–0.42]	
ICC-clusters	0.21	[0.17–0.27]		0.32	[0.25–0.39]		0.31	[0.24–0.39]	
**σ^2^_villages_**	0.59	[0.39–0.92]		1.35	[0.91–1.99]		1.24	[0.82–1.86]	
ICC-villages	0.14	[0.10–0.21]		0.28	[0.21–0.37]		0.26	[0.19–0.35]	

a 60 missing values resulted in a sample of N = 7632: the combination of the variables ‘other household members previously diagnosed with and treated for HCV’, ‘pre-study history of HCV testing (participant)’, and ‘perceived health status at the time of the survey’.

The higher odds of combined testing uptake in the community-based arm persisted after adjustment for individual characteristics (Table 3, Model 2), as indicated by the aOR value of 2.18, 95% CI [1.28–3.73] (p = 0.004). In addition, the significant non-linear effect of age indicates that younger and older participants were less likely to actually have HCV RDT or HCV RDT/RNA testing (aOR_age: 1.11, 95% CI [1.05–1.16]; aOR_age^2^: 0.999, [0.998–0.9994], p < 0.001). Participants working ≤7 days per week and <8 h per day (aOR: 0.33, 95% CI [0.28–0.40], p < 0.001), those with a heavy workload (i.e., working 7 days per week and ≥ 8 h per day (aOR: 0.25, 95% CI [0.28–0.30], p < 0.001)), those with household members previously diagnosed with/treated for HCV (aOR: 0.19, 95% CI [0.10–0.37], p < 0.001), those who had themselves a history of HCV testing (aOR: 0.13, 95% CI [0.09–0.20], p < 0.001), and those perceiving they had moderate/poor/very poor health status (aOR: 0.39, 95% CI [0.33–0.41], p < 0.001) were all less likely to actually have HCV RDT testing. On the contrary, HCV RDT uptake was more likely in females (aOR: 1.16, 95% CI [1.02–1.32], 0.024), in married/cohabitating participants (aOR: 2.01, 95% CI [1.13–2.84], p < 0.001), those widowed/divorced/separated (aOR: 1.97, 95% CI [1.36–2.84]), and participants in Siem Reap province (aOR: 3.12, 95% CI [1.82–5.34], p < 0.001). The higher odds of combined testing uptake in the community-based arm were confirmed in the sensitivity analysis where differences between arms were corrected using the IPW technique (aOR: 2.30 95% CI [1.29–1.10], p = 0.005; Supplementary File 1, Table S3).

### Secondary outcomes

Overall, the uptake of confirmation testing (i.e., having a positive HCV RDT and having a blood sample collected for HCV RNA testing) in the 5590 participants tested in both strategies was 1.7% (95% CI [1.3%–2.0%]) (n = 93; one participant with a positive RDT subsequently refused to give a blood sample, Table 4). Confirmation testing uptake was higher in the facility-based strategy, at 2.2% (95% CI [1.6%–2.7%]), compared with 1.2% (95% CI [0.8%–1.6%]) in the community-based strategy (p = 0.008). Regarding the confirmation testing uptake among participants testing positive to RDT, proportions were 98.2% and 100% for the facility-based strategy and the community-based strategy respectively. Table 4 also shows an active-HCV infection detection proportion (i.e., positive RDT and detectable subsequent RNA test) of 1.3% [0.1%–1.6%] among the 5590 who actually had an HCV RDT. No significant difference was found between the strategies in terms of active case detection (39 active cases (1.5% [1.0%-1.9]) in the facility-based strategy and 32 (1.1% [0.7%–1.4%]) in the community-based strategy (p = 0.152)). In addition, no difference was found for individual characteristics, including age (median [IQR] of 67 [60–70] years and 62 [57–67] years among active cases in the facility-based and community-based strategies, respectively (p = 0.278)). Finally, a total of 68 of the 71 HCV active infection cases had linkage to care (95.8%; three participants refused to go to a referral hospital): 97.4% (n = 38 out of 39) and 93.4% (n = 30 out of 32) for the facility-based and the community-based strategies, respectively. Among the 38 active cases in the facility-based strategy who were linked to care, 36 (94.7%) were eligible and initiated DAA treatment: one ineligible was already on HCV treatment and one had hepatocellular carcinoma. All 30 active cases (100%) in the community-based strategy were linked to care and were eligible for DAA. Four patients were lost to follow-up for the facility-based strategy and two for the community-based strategy. Overall, 60 of the 66 participants who started DAA treatment completed it, 58 achieved SVR12 and two (both randomized into the community-based strategy) had still detectable HCV RNA at week 24.

**Table 4:** Summary of data analyzed and mixed effects model to assess the effect of the community-based strategy with respect to the facility-based strategy on primary and secondary outcomes.

Variable	Facility-based N (%)	Community-based N (%)	ICC_villages_ [95% CI]	ICC_clusters_ [95% CI]	aOR [95% CI]	p-value
**Intention-to-treat**			–	–	–	–
No of participants	5098	4687	–	–	–	–
**Per protocol**						
No. of participants	3861	3831				
No. HCV RDT or HCV RDT/RNA (combined testing uptake)	2600	2990	–	–	–	–
No. positive HCV RDT	57	37				
No. HCV RDT & HCV RNA (confirmation testing uptake)	56	37	–	–	–	–
No. positive HCV RDT & detectable HCV RNA (active infection detection)	39	32	–	–	–	–
No. of patients linked to care	38	30	–	–	–	–
No of participants on DAA treatment	36	30	–	–	–	–

Community-based strategy versus facility-based strategy (ref.)	Facility-based N (%)	Community-based N (%)	ICC_villages_ [95% CI]	ICC_clusters_ [95% CI]	aOR [95% CI]	p-value
**Primary outcome**						
**Intention-to-treat**						
HCV RDT or HCV RDT/RNA (combined testing uptake)	2600/5098 (51.0)	2990/4687 (63.8)	0.14 [0.10–0.21]	0.29 [0.18–0.45]	1.91 [1.29–2.83]	0.001
**Per protocol**						
HCV RDT or HCV RDT/RNA (combined testing uptake)—unadjusted	2600/3861 (67.3)	2990/3831 (78.1)	0.28 [0.21–0.37]	0.21 [0.17–0.27]	2.44 [1.41–4.23]	<0.001
HCV RDT or HCV RDT/RNA (combined testing uptake)—adjusted^a^			0.26 [0.19–0.35]	0.31 [0.24–0.39]	2.18 [1.28–3.73]	0.004
**Secondary outcomes**						
HCV RDT & HCV RNA (confirmation testing uptake)—adjusted^b^	56/2600 (2.2)	37/2990 (1.2)	0.18 [0.01–4.33]	0.42 [0.08–2.05]	0.55 [0.32–0.94]	0.028
HCV RNA (confirmation testing uptake specific to positive RDT)	56/57 (98.2)	57/57 (100)	–	–	–	–
Positive HCV RDT & detectable HCV RNA (active infection detection)—adjusted^b^	39/2600 (1.5)	32/2600 (1.1)	0.29 [0.03–2.73]	0.18 [0.003–10.61]	0.67 [0.38–1.19]	0.171
Linkage to care—adjusted^c^	38/39 (97.4)	30/32 (93.8)	–	–	0.39 [0.34–4.56]	0.457
On DAA treatment^d^	36/38 (94.7)	30/30 (100)	–	–	–	–

a Adjusted for age, age^2^, gender, marital status, activity workload, other household member(s) previously diagnosed with and treated for HCV, pre-study history of HCV testing (participant), perceived health status and province.

b Adjusted for age, age^2^, gender, marital status, activity workload, other household member(s) previously diagnosed with and treated for HCV, pre-study history of HCV testing (participant), and perceived health status; adjusted for age, age^2^, gender, marital status, activity workload, other household member(s) previously diagnosed with and treated for HCV, pre-study history of HCV testing (participant), perceived health status.

c Mixed effects model was not better than single-level logistic model (LR-test p = 0.259).

d Not possible to estimate a model given that 100% of patients linked to care in the community-based arm were eligible and received DAA treatment.

Table 4 shows the adjusted odd-ratios for both strategies according to the study's secondary outcomes. Confirmation testing uptake (i.e., HCV RDT & HCV RNA) was less likely in the community-based strategy (aOR: 0.55, 95% CI [0.32–0.94], p = 0.028). However, no difference was found between the strategies concerning active HCV infection detection (p = 0.171) or linkage to care among active cases (p = 0.457). Sensitivity analysis resulted in no significant difference between arms concerning confirmation testing (p = 0.145), and similar results were estimated for the other secondary outcomes (Supplementary File 1, Table S4).

## Discussion

This cluster randomized controlled trial, conducted among adults aged 40 years or more in Cambodia in 2022–2023, demonstrated the effectiveness of the community-based strategy, with 2.18 times higher odds of combined testing uptake (HCV RDT or HCV RDT/RNA) in comparison with the facility-based strategy. However, confirmation testing uptake was less likely in the community-based strategy, while no differences between arms were found in terms of active HCV infection detection and linkage to care. In addition, 94.7% and 100% of active cases were initiated DAA treatment in the facility-based and community-based arms respectively. These results show that innovative strategies such as the home-based HCV screening and confirmation by CHW tested in this study are relatively more effective in the detection of new HCV active infections, without any disruption of the continuum of care until treatment completion.

In the past decade, several interventions to improve HCV testing have been documented, although they are mostly conducted in facility settings and focus on specific populations.^2^^,^^3^ The community-based strategy tested in this study responds to the need of innovative interventions to inform public policy for the scale-up of HCV services.^2^ The present study demonstrates the feasibility, relevance and effectiveness of demedicalizing HCV screening, by shifting these tasks to CHWs. It demonstrates that the implementation of screening and blood collection tools (i.e., RDT and DBS) easy to use by non-healthcare personnel^31^ is suitable in outreach settings providing an alternative for further decentralization of HCV services. Although several previous community-based studies^10^^,^^11^^,^^13^^,^^32^ highlighted the importance of CHWs—including peers—in HCV activities,^9^^,^^33^ ours is the first to show that with appropriate capacity building, CHWs can become key players in the first stage of the HCV cascade of care without compromising the clinical performance of HCV diagnosis.^31^^,^^34^ Specifically, training CHWs in demedicalized HCV screening and diagnosis, especially among vulnerable populations, could significantly impact the country's national HCV program in several ways: first, it could help to overcome structural barriers by simplifying access to HCV screening, diagnosis and linkage to care services, as well as by reducing health workers' workloads in HC. As in previous studies, these results suggest that involving CWHs contributes to the simplification of HCV services, a crucial aspect to reach vulnerable populations, especially in rural settings.^4^^,^^6^^,^^22^ Second, CHWs' close geographical proximity to vulnerable communities could help to identify at-risk populations, including hard-to-reach groups, especially people far from the healthcare system. Our study also highlighted that HCV testing by CHWs in persons' homes reduced demographic and socioeconomic disparities in uptake; more specifically, relatively more women, people without a partner, and individuals with no activity had higher odds of combined testing uptake in the community-based strategy than in the facility-based one. However, further areas of simplification of HCV screening and diagnosis could be contemplated, for example, by integrating our community-based strategy with tools such as the Xpert HCV Viral Load Fingerstick Point-of-Care in primary care settings.^35^ Although our results demonstrate that once people became aware of their HCV positive status, linkage to care, access to treatment and retention did not depend on the strategy used, a point-of-care strategy could allow a more streamlines follow-up (i.e., by shortening the time for blood sample analysis).

In the wider context of the Cambodian national plan to eliminate HCV by 2030, the community-based screening strategy we used in ANRS 12384 Cam-C could provide a major contribution to the construction of HCV micro-elimination strategies (by targeting specific populations), especially by creating synergies with facility-based activities.^15^ Our results highlighted that the two strategies we compared complement each another, as HCV combined testing uptake was more likely in the community-based strategy, whereas confirmation testing uptake was more likely in the facility-based strategy. In addition, our results demonstrated that HCV care seeking behavior did not depend on the type of the strategy. Overall, HCV seroprevalence was 1.7% while the prevalence of active HCV cases was 1.3%. These values are lower than the respective 5.1% and 3.6% reported in a previous study for adults aged ≥45 years in the Moung Ruessei district of Battambang province.^17^ However, this may be explained by differences between both studies in terms of time-frames, objectives and designs, as well as different geographical and demographic settings. Surprisingly, HCV prevalence in participants in the community-based strategy was lower than in participants in the facility-based strategy. One possible explanation for this is that individuals in the latter may have been at a higher risk of HCV infection. This is particularly true for working age men, a group most likely missed when the CHWs contacted households about the community-based HCV screening offer. The higher percentage of at-risk individuals going to an HC for free HCV testing would therefore translate into a higher detection rate.

Photos of all HCV RDT tests were taken for both strategies in order to minimize the risk of misinterpretation by CHWs, and to ensure accurate results. Moreover, no difference was observed between the community- and facility-based strategies in terms of linkage to care, access to DAA and completion of treatment in persons who were diagnosed HCV positive. Looking forward, our study results underline that eliminating HCV requires a more comprehensive approach, with integrated prevention, screening, diagnosis and treatment services serving as a cornerstone. Specifically, integrated HCV services (prevention, screening, diagnosis and treatment) with services for other diseases would ensure that the HCV cascade of care is managed effectively.^36^ This is particularly true in developing countries, where different programs such as the HIV or malaria programs have already proven to be effective in reaching vulnerable.^7^^,^^37^ Despite the added-value of the community-based strategy, the overall relatively lower HCV prevalence we found in comparison with previous studies, suggests that integrating these two strategies into Cambodia's national HCV elimination program requires the identification of high-prevalence areas in order to have a greater impact on HCV RDT uptake and active HCV case detection. Targeting specific populations, as demonstrated in Vietnam^11^^,^^38^ and previous studies in Cambodia,^17^^,^^19^^,^^20^^,^^39^^,^^40^ is crucial to increase the effectiveness of our community-based HCV RDT strategy in synergy with the populations reached by the facility-based strategy. Implementing such micro-elimination strategies as part of a comprehensive framework could ensure that structural barriers (e.g., HC organization, a lack of human and material resources, intellectual property, etc.) and individual barriers (e.g., HCV awareness, health literacy, treatment adherence, etc.) to accessing healthcare services are overcome.^14^^,^^36^

This study has limitations. First, the small number of positive HCV active cases limits the statistical power to identify significant differences between the two screening strategies which we tested. However, the large sample size, and therefore good statistical power, ensures that our results highlighting the effectiveness of the community-based strategy are robust. In addition, the large sample size provided enough heterogeneity to identify specific groups to target in future community-based strategies, such as middle-aged men, especially those with heavy work schedules. Second, the fact that the study did not investigate the modalities of the real-world implementation of HCV RDT testing by CHWs limits our knowledge as to whether our community-based strategy would be operationally and financially sustainable over the long term. However, the lessons learned from Cambodia's successful ongoing community-based strategy to eradicate malaria suggest that it would.^41^ Although the success of the latter strategy reflects more operational than financial sustainability, the fact that CHWs working in coordination with HC has proven to be feasible and sustainable over the long-term, suggests the long-term financial sustainability of their involvement in various programs including those focusing on HCV elimination.

### Conclusion

Our study is one of the relatively few randomized controlled trials in the literature investigating novel strategies for HCV screening and active case detection, and one of the very few in the WHO Western Pacific region. It provides evidence for the added value of using a community-based strategy to overcome structural barriers to HCV RDT or HCV RDT/RNA uptake and to access to HCV services in general. It also highlights that our community-based strategy and the facility-based strategy are complementary and synergic; CHWs improve individuals' first contact with HCV services while healthcare workers ensure the continuum of HCV care until treatment completion. Integrating this trial's community-based strategy into the Cambodian national HCV program, which has set a target for eliminating HCV by 2030, could improve this program's effectiveness. In Cambodia, and other countries with a similar HCV epidemic profile, our community-based strategy could play an important role in HCV surveillance and the cascade of care. Ongoing analysis on cost-effectiveness of the community-based strategy will complete the results of the present article. In addition, scaling it up as part of a framework for implementation research is necessary to produce evidence ensuring its operational and financial sustainability.

## Contributors

DK Software, investigation, formal analysis, project administration, writing - original draft, writing - reviewing & editing, visualization. LST Conceptualization, methodology, software, investigation, formal analysis, writing - original draft, writing - reviewing & editing, visualization. SN Conceptualization, project administration, supervision, validation, visualization, writing - reviewing & editing. SS Project administration, writing - reviewing & editing. LM Conceptualization, methodology, validation, writing - reviewing & editing. EM writing - reviewing & editing. DM editing. CLP Writing - Project administration, writing - reviewing & editing. CC Project administration, writing - reviewing & editing. KH Project administration, writing - reviewing & editing. SM Project administration, data curation, software, writing - reviewing & editing. JCDV Conceptualization, methodology, investigation, writing - reviewing & editing. OS Conceptualization, formal analysis, funding acquisition, investigation, methodology, project administration, validation, writing - reviewing & editing. VS Conceptualization, methodology, funding acquisition, investigation, validation, supervision, writing - reviewing & editing. Furthermore, DK, LST, SN, CC, CLP, KH, SM, and OS had access to raw data. DK, LST, SN, LM, CLP, JCDV, OS, and VS verified the data. All authors substantially contributed to the manuscript, approved the final version and the decision to submit for publication.

## Data sharing statement

The study protocol and data collection documents will be made available upon reasonable request. Requests for the data reported in this article can be made by submitting a study proposal to the scientific board of the ANRS 12384 Cam-C study (khuondyna@uhs.edu.kh/vonthanak@uhs.edu.kh). Requests will be evaluated for compatibility with the Cam-C study and for overlap with our ongoing and future planned research work, in accordance with the Cambodian and French Ethics Committees' guidelines.

## Declaration of interests

Laurence Meyer's institution receives financing for operating costs and personal from ANRS MIE. All other authors declare that they have no competing interests, financial or otherwise, concerning this work.

## Appendix A Supplementary data

ANRS Protocol

Supplementary File–Tables

CONSORT Extension for Cluster Trials 2012 Checklist_CamC
