# Supplementary material for: Community-based versus facility-based services to improve hepatitis C screening in Cambodia: a cluster randomized controlled trial (ANRS 12384 Cam-C study)
Source: Lancet Reg Health West Pac. 2025 Oct 8;63:101703. doi: 10.1016/j.lanwpc.2025.101703 (PMC12538039; doi:10.1016/j.lanwpc.2025.101703)
Supplement: ANRS Protocol [file mmc1.pdf]

ANRS 12384 Cam-C study

Community versus facility-based services to improve the screening of active HCV infection in Cambodia: a cluster randomized controlled trial

Protocol version 5.0 – July. 2023

Clinicaltrial.gov registration number NCT03992313

Ethics committee approvals

Cambodia: Name of the committee: National Ethics Committee for Health Research (NECHR)

Date of approval: February 25, 2019 (N°047)

Sponsor:

Inserm-ANRS

French National Institute for Health and Medical Research (Inserm)

ANRS Emerging Infectious Diseases – Autonomous agency of Inserm (ANRS EID)

2 rue d'Oradour-sur-Glane

75015 PARIS

FRANCE

Tél. : 01 53 94 60 00 - Fax : 01 53 94 60 01

Coordinating investigators:

Pr SAPHONN Vonthanak<br>University of Health Sciences<br>73 Preah Monivong Bvd<br>Phnom Penh, Cambodia<br>Email: vonthanak@uhs.edu.kh /	Pr Jean-Charles DUCLOS VALLEE<br>Inserm U785 UMR-S 1193<br>Service d'hépatologie<br>Centre Hepato-Biliaire - DHU Hepatinov<br>Hôpital Paul Brousse<br>12-14 avenue Paul Vaillant-Couturier<br>94800 Villejuif, France<br>Email: jean-charles.duclos-vallee@aphp.fr

Confidentiality clause : This document contains information that is the property of the sponsor and entrusted to you in confidence for consideration by yourself, your team, members of the Ethics Committee and Competent Authority concerned. Information contained in this document shall not be communicated to third parties without the prior written consent of the sponsor of the study, except as necessary to obtain informed consent from people who could participate to the study.

PAGE DE SIGNATURES

Community versus facility-based services to improve the screening of active HCV infection in Cambodia: a cluster randomized controlled trial

Protocol version 5.0 – Jun.2023

Clinicaltrial.gov registration number NCT03992313

Ethics committee approvals

Cambodia: Name of the committee: ______NECHR______________________________________

Date of approval: ______________________________________

Sponsor signature

Inserm-ANRS

French National Institute for Health and Medical Research

ANRS Emerging Infectious Diseases (ANRS EID) – Autonomous agency of Inserm

Director: Pr Yazdan Yazdanpanah

2 rue d'Oradour-sur-Glane

75015 Paris, France

Phone: +33 153 94 60 46 / 60 00

Fax : +33 153 94 60 01

Paris, Date :

Name : Pr Yazdan Yazdanpanah

Signature

Coordinating investigators signature

Pr SAPHONN Vonthanak

University of Health Sciences

73 Preah Monivong Bvd

Phnom Penh, Cambodia

Email: vonthanak@uhs.edu.kh /

Date:

Name : Pr SAPHONN Vonthanak

Signature

Pr Jean-Charles DUCLOS VALLEE

Inserm U785 UMR-S 1193

Service d'hépatologie

Centre Hepato-Biliaire - DHU Hepatinov Hôpital Paul Brousse

12-14 avenue Paul Vaillant-Couturier

94800 Villejuif

Email: jean-charles.duclos-vallee@aphp.fr

Date :

Name : Pr Jean-Charles DUCLOS VALLEE

Signature

Contents

STUDY PROTOCOL VERSIONS ... *6*

STUDY TEAM MEMBERS ... *10*

1. STUDY SUMMARY ... *13*

2. STUDY SYNOPSIS ... *16*

3. SCIENTIFIC RATIONALE ... *18*

HCV situation in Cambodia ... 18

Cascade of care for HCV infection in LMICs ... 19

Health care system organization in Cambodia ... 21

Study main hypothesis ... 21

4. OBJECTIVES OF THE STUDY ... *22*

Primary objective ... 22

Secondary objectives ... 22

5. METHODOLOGY ... *22*

Study design ... 22

Study overview ... 22

Tentative study agenda ... 23

6. STUDY POPULATION ... *23*

Inclusion criteria ... 23

Non-inclusion criteria ... 23

7. STUDY ENDPOINTS ... *24*

Primary endpoint ... 24

Secondary endpoints ... 24

8. STRATEGIES AND TREATMENTS ... *24*

Strategies ... 24

Treatments used in the study ... 26

9. STUDY IMPLEMENTATION ... *29*

Study schedule ... 29

Patients' participation in the study ... 30

Withdrawal of consent and loss to follow-up ... 34

10. LABORATORY EVALUATIONS ... *35*

Blood samples collection and tests ... 35

HCV RNA viral load ... 36

Biobank ... 36

12. TRIAL BOARDS AND COMMITTEES **45**

12.1 Trial scientific committee **45**

Composition **45**

Meeting agenda **45**

Role 45

12.2 Data Safety & Monitoring Board (DSMB) **45**

Composition **45**

Meeting agenda **45**

Role 46

12.3 Decision Support Committee (DSC) **46**

13. COORDINATION, MONITORING, DATA MANAGEMENT ***47***

Coordination **47**

Trial documents **47**

Data management **47**

14. STATISTICAL ANALYSES ***49***

Calculation of number of patients needed ***49***

Statistical analysis plan **50**

15. ACCEPTIBILTY STUDY ***51***

15.1. Summary ***51***

15.2. Research questions ***52***

15.3. Study objectives ***52***

15.4. Methods ***53***

16. SCIENTIFIC COMMUNICATION ***57***

17. ETHICS AND LEGAL CONSIDERATIONS ***57***

18. ACCESS TO DATA AND SPECIMENS ***60***

19. DUTIES OF INVESTIGATORS ***60***

20. REFERENCES ***61***

STUDY PROTOCOL VERSIONS

Version	Date	Amendment	Modifications
**1.0**	**February 2019**		
**2.0**	**October 2020**	**1**	**1-New sample size estimation**<br>**2-Modification of information process for facility arm**<br>**3-Perform socioeconomic questionnaires during screening phase**<br>**4-Add 2019 NSP and guidelines in the background section**<br>**5-Modification of the randomization process**<br>**6-Modification of tentative study agenda**<br>**7-Modification of data collection section by changing to e-CRF for screening phase**<br>**8-Addition of cost-effectiveness section**
**3.0**	**March 2021**	**2**	**1- Modification of information process**<br>**2- Modification of HCV RNA quantification system for the facility arm**<br>**3- New information about the Sponsor**<br>**4- Information on drugs supply and delivery**
**4.0**	**April 2022**	**3**	**1- Annual resubmission**<br>**2- Modification of HCV RNA quantification system and location for the facility arm**<br>**3- New co-investigators for virology**<br>**4- New samples volumes for the therapeutic phase**

			**5- New information about the sponsor**<br>**6- Update of the information lettres**
**5.0**	**July 2023**	**4**	**1 -Added a new qualitative study in part 15.**

LIST OF ABBREVIATIONS

AIDS	Acquired immunodeficiency syndrome
ALT	Alanine aminotransferase
ANRS EID	ANRS Emerging Infectious Diseases
ARV	Antiretroviral drugs
ART	Antiretroviral therapy
AST	Aspartate aminotransferase
BMI	Body mass index
CBC	Complete blood count
CDC	Communicable Disease Control
CHAI	Clinton Health Access Initiative
CRF	Case report form
CHW	Community Health Workers
DAA	Direct Acting Antiviral
DBS	Dried dlood spots
DSC	Decision Support Committee
DSMB	Data safety & monitoring board
ELISA	Enzyme-linked immunosorbent assay
GCLP	Good clinical laboratory practice
GCP	Good clinical practice
GRT	Genotyping Resistance Test
HCC	Hepato Cellular Carcinoma
HCV Ab	Hepatitis C virus antibody
HBs Ag	Hepatitis B virus surface antigen
HIV	Human immunodeficiency virus
HC	Health Centers
IMP	Investigational medicinal product
IPC	Institut Pasteur du Cambodge
LMIC	Low and middle income countries
MIC	Minimum inhibitory concentration
MDMC	Monitoring and data management center
MDR	Multi drug resistant
NCD	Non Communicable Diseases
NCHADS	National Center for HIV/AIDS, Dermatology and STD
NRTI	Nucleoside reverse transcriptase inhibitor
OD	Operational District
PHD	Provincial Health Departments
PCR	Polymerase chain reaction

PI	Protease Inhibitor
PLHIV	People living with HIV
PK	Pharmacokinetic
PMTCT	Prevention of mother-to-child transmission
QS	
RDT	Rapid Diagnostic Test
RNA	Ribonucleic acid
RH	Referral Hospital
(S)AE	(Serious) Adverse event
(S)AR	(Serious) Adverse reaction
SmPC	Summary of the product characteristics
SOC	System organ class
SOPs	Standardized operating procedures
SUSAR	Suspected unexpected serious adverse reaction
TB	Tuberculosis
TDF	Tenofovir
TMF	Study master file
TWG	Technical Working Group
UHS	University of Health Sciences
USAID	United States agency for international development
WHO	World health organization

STUDY TEAM MEMBERS

SPONSOR	
**Inserm / ANRS**<br>2 rue d'Oradour-sur-Glane<br>75013 Paris, France.<br>Phone: +33 1 53 94 60 00	
Célia BOUHARATI<br>Clinical Research Department<br>Phone: +33 1 53 94 60 34<br>E-mail: celia.bouharati @anrs.fr	Dr Alpha DIALLO, Anaïs BOSTON<br>Pharmacovigilance Department<br>Phone: +33 153 94 60 40<br>E-mail: alpha.diallo@anrs.fr; anais.boston@anrs.fr

COORDINATING INVESTIGATORS	
Pr SAPHONN Vonthanak<br>University of Health Sciences<br>73 Preah Monivong Bvd<br>Phnom Penh, Cambodia<br>Email: vonthanak@uhs.edu.kh /	Pr Jean-Charles DUCLOS VALLEE<br>Inserm U785 UMR-S 1193<br>Service d'hépatologie<br>Centre Hepato-Biliaire - DHU Hepatinov Hôpital Paul Brousse<br>12-14 avenue Paul Vaillant-Couturier<br>94800 Villejuif, France<br>Email: jean-charles.duclos-vallee@aphp.fr

CO-INVESTIGATORS	
**Study coordinator**<br>Dr NETH Sansothy<br>University of Health Sciences<br>73 Preah Monivong Bvd<br>Phnom Penh, Cambodia<br>E-mail: nsothy@uhs.edu.kh	**Clinical coordinator**<br>Dr Olivier SEGERAL<br>ANRS<br>University of Health Sciences<br>73 Preah Monivong Bvd<br>Phnom Penh, Cambodia<br>Phone: +855 12 479 313<br>E-mail: olivier_segeral@uhs.edu.kh

MONITORING AND DATA MANAGEMENT CENTER (MDMC)

Grant Management Office, UHS

Monitoring / statistical analysis

Dr SUY Sovanthida

University of Health Sciences

Phnom Penh, Cambodia

Email: sovanthidasuy@uhs.edu.kh

System development and data managing:

Pr MAM Sovatha, UHS

University of Health Sciences

Phnom Penh, Cambodia

Email: sovatha@uhs.edu.kh

Methodology/Biostatistics

Pr Laurence MEYER

INSERM SC10/US019
Essais thérapeutiques et maladies infectieuses
Centre Inserm

16 avenue Paul Vaillant-Couturier 94807
Villejuif Cedex, France

EMail : laurence.meyer@inserm.fr

Pr Bruno GIRAUDEAU

INSERM U1246 - SPHERE

2 Bd Tonnellé

37044 Tours cedex 9, France

Email: Bruno.giraudeau@univ-tours.fr

COST-EFFECTIVENESS ANALYSIS	
Dr Luis SAGAON TEYSSIER<br>UMR912 SESSTIM/ORS PACA<br>SESSTIM<br>ESSEM<br>IHU<br>19-21 Bd Jean Moulin<br>13005, MARSEILLE, France<br>Email : luis.sagaon-teyssier@inserm.fr	

VIROLOGY	
Dr PHOEUNG Chan Leakhena<br>Head of laboratory<br>University of Health Sciences<br>Laboratoire Merieux<br>73 Preah Monivong Bvd<br>Phnom Penh, Cambodia	Dr SEK Mardy and Dr Eric NERIENNET<br>Fondation Merieux<br>17 rue Bourgerat<br>69002, Lyon, France<br>Email: enerrienet@gmail.com

1. STUDY SUMMARY

Clinicaltrial Id: NCT03992313

Title of Study: Community versus facility-based services to improve the screening of active HCV infection in Cambodia: a cluster randomized controlled trial

Short title – Sponsor N°: -ANRS 12384 Cam-C

Sponsor

Inserm-ANRS

French National Institute for Health and Medical Research

ANRS Emerging Infectious Diseases (ANRS EID) – Autonomous agency of Inserm

Coordinating Investigator(s): Pr SAPHONN Vonthanak, Pr Jean Charles DUCLOS VALLEE

Participating countries: CAMBODIA

Objectives

Principal objective: to compare the effectiveness of a community-based intervention to a facility-based intervention to improve the combined-testing uptake (Antibody + RNA) of HCV infection among general population aged more than 40 years old in Cambodia

Secondary objectives

- To compare the HCV antibody testing uptake between the 2 arms for the eligible population
- To compare the active case detection rate between the 2 arms for the eligible population
- To compare the linkage to care between the 2 arms for those with active infection
- To compare the cost-effectiveness of the two strategies
- To evaluate the treatment uptake
- To evaluate the effectiveness and safety of a 12-week dual-therapy of direct-acting antiviral (DAA) treatment

Methodology: two-arms cluster-randomized controlled trial. Clusters are defined as a group of 50 households

Expected enrolment 8000 patients in 160 clusters located in 2 provinces (Kompong Cham and Siem Reap)

Outcomes **Primary outcome: combined-testing uptake** defined as the number of persons tested for HCV RDT AND HCV RNA and aware of their status among the total number of persons eligible residing in the region where the intervention takes place

Secondary outcomes:

The following secondary endpoints will be measured and compared between the 2 arms:

- **HCV antibody testing uptake** defined as the number of persons tested for HCV RDT and aware of their status among the total number of persons eligible residing in the region where the intervention takes place
- **Active case detection rate** defined as the number of persons with HCV active infection (positive HCV Ab and positive HCV RNA) and results given and explained among the total number of persons eligible residing in the region where the intervention takes place
- **Linkage to care** defined as the number of persons with at least one consultation in the Provincial Hospital among the estimated total number of persons with active infection residing in the region where the intervention takes place

The following secondary endpoints will be measured for the total population and not compared:

- **Treatment uptake** defined as the number of people initiating HCV treatment among the total number of persons with active infection linked to care
- **Liver-related morbidity and mortality** (decompensated cirrhosis, HCC)
- Proportion of patients with sustained virologic response 12 weeks after discontinuation of treatment (**SVR12**)
- Proportion of patients with **treatment failure** defined as absence of SVR12 or missing HCV-RNA at 12 weeks post-treatment (PT12) due to treatment discontinuation for AEs or death

Eligibility **Inclusion criteria**

- All persons aged more than 40 years old
- Residing in the study area
- Informed consent obtained with oral information given and explained and the consent form signed by the participant and the nurse hired by the study at the latest the time of the RDT realization

Non-inclusion criteria

- Known positive HCV status with previous HCV treatment
- Severe disease present at inclusion involving life threatening
- Concurrent participation in any other clinical study without written agreement of the two study teams

Intervention

Arm 1: Facility-based testing intervention

Community Health Workers (CHWs) will provide information on the possibility to be tested in health centers for HCV infection. Information will be provided using information sheet and a dedicated leaflet in the selected households. If the participant agrees to participate, she/he will receive a voucher to go to referral health center for testing. In health center, after signature of consent form, HCV testing will be done using HCV RDT on a finger stick capillary whole blood. Results will be available in 15 minutes. In case of positive HCV RDT, an immediate blood sample collection (5 mL of whole blood) will be done in health center and sent to Provincial hospital laboratory to collect plasma. Plasma will be sent to University of Health Sciences (UHS) to perform HCV RNA . Results will be sent back to the health center and nurses will be in charge to give result to the participant and to refer to care in case of active infection (systematic appointment + phone call if the participant doesn't come)

Arm 2: Community-based testing intervention

After a dedicated training, CHWs will do the HCV RDT on a finger stick capillary whole blood directly in the household of participant. Information will be provided by the CHW using information sheet and a dedicated leaflet. If the participant agrees to participate, the signed consent form will be collected at this step before any RDT collection. In case of structural or societal barriers for household testing, it will be possible to provide testing in a specific location in the village. The onsite visits will be planned with the head of village who will be in charge to inform the population about the study. In case of absence of participant during the first visit, a second visit will be scheduled. In absence of participant during the second visit, no additional visit will be planned.

In case of positive HCV RDT, 5 blood spots will be collected immediately on DBS, dried at ambient temperature and put in an individual plastic bag with the ID number and sent to UHS (Rodolphe Merieux laboratory) for HCV RNA extraction and amplification. Results will be sent back to the referral health center and nurses will be in charge to give result to the participant and to refer to care in case of active infection.

Treatment phase

For positive HCV RNA, a consultation in the Provincial Hospital will be planned. The baseline assessment will include questionnaires (risk behaviours and socio-economic status), clinical exam, blood sampling and liver ultrasound. Symptomatic cirrhotic patients will be referred to a National Hospital in Phnom Penh to a hepatology department. For the others patients, DAA treatment using sofosbuvir and daclatasvir combination for 12 weeks (Cambodian Essential Medicine List 2018) will be proposed, after checking the result of creatinine and the possible drug-drug interactions.

Statistical methods

The study will be powered to detect an increase of HCV combined-testing uptake from 60%in the facility arm to 80% in the community arm among subjects older than 40 years.

The following assumptions and definitions will be used for the sample size calculation:

- an intra-class correlation of 0.01

- significance level α= 0.05

- power (1-β) = 80%

- clusters size: 50 persons aged more than 40 years old

- Design effect: 1.5

When accounting for the above-mentioned assumptions, 300 patients will need to be screened for HCV RNA and so, 6000 individuals aged above 40 years old will need to be screened by RDT (5% of patients are expected to have a positive HCV Ab.). With an estimated median testing uptake of 60-70%, if we want to reach 6000 participants screened, it would be necessary to propose testing to 8000 participants from 160 clusters (80 clusters per intervention).

Estimated planning or study timetable

First inclusion: May 2022

Inclusion time: 6 months.

Time of follow-up for each participant: 6 months

Last visit for the last patient: April 2023

2. STUDY SYNOPSIS

Study schedule for the screening phase

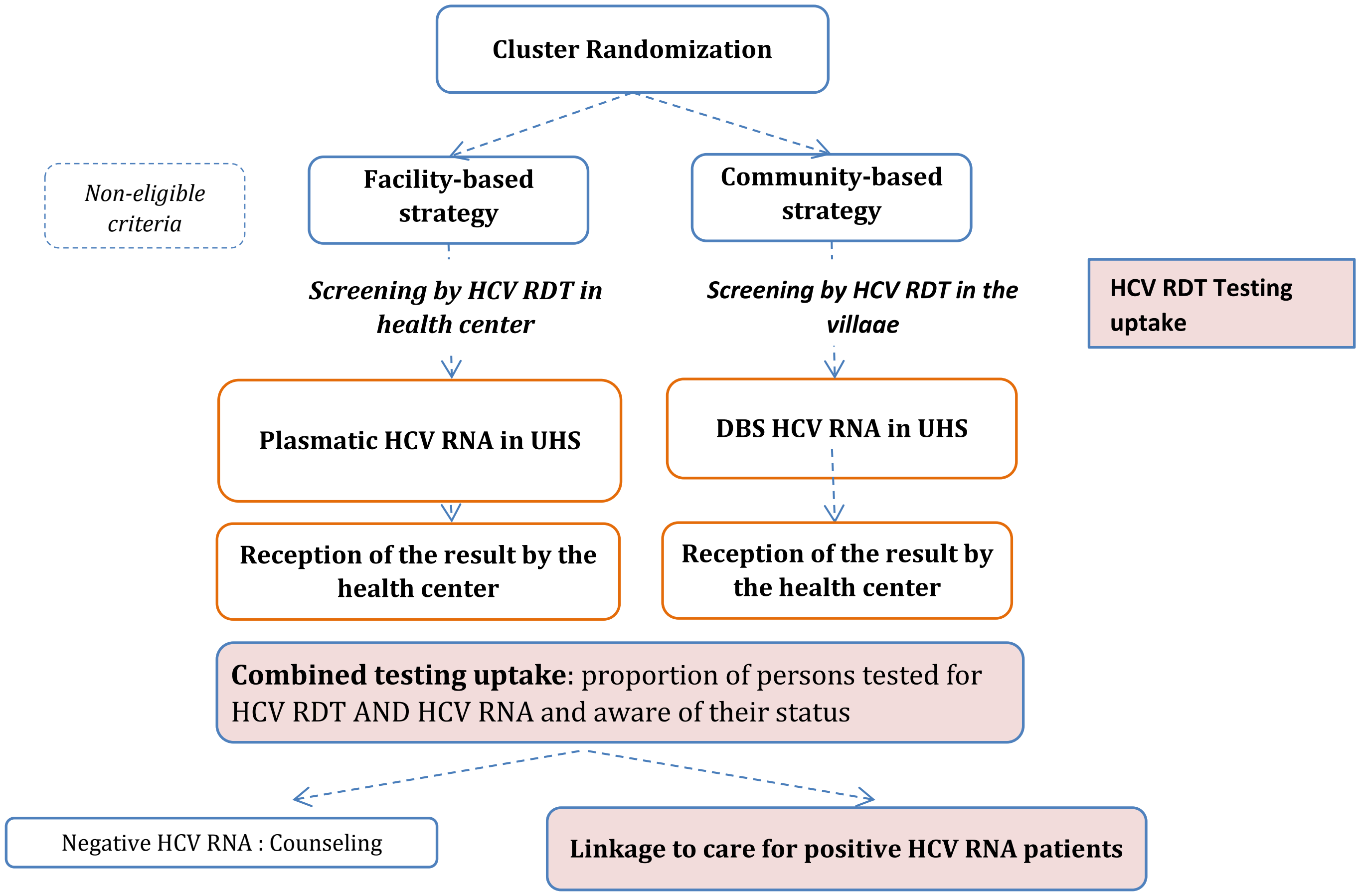

Study schedule for the therapeutic phase

	Therapeutic phase for positive HCV RNA	W4	W8	W12	W24	W28
Consent form	X					
Eligibility criteria	X					
Consultation and clinical exam	x	x	x	x	x	x
HIV RDT	x					
HBs Ag RDT	x					
HCV RNA					x	
AST / ALT	x	x	x	x	x	
Creatinine	x	x	x	x		
CBC	x	x			x	
Pregnancy test[4]	x	x	x			
PT	x				x[3]	
Bilirubin	x				x[3]	
Albumin	x				x[3]	
Fasting glycemia	x					
Score APRI / FIB4	x					
Liver ultra-sound	x				x[3]	
Biobank	x			x	x	
DAA delivery	x	x	x			
Adherence assessment		x	x	x		

(1) For patients in arm 1

(2) For patients in arm 2

(3) For cirrhotic patients

(4) Pregnancy test will be performed at inclusion, W4 and W8 for all women of childbearing age

3. SCIENTIFIC RATIONALE

HCV situation in Cambodia

In Cambodia, a Southeast-Asian country of estimated 15,7 million (2016 – World Bank) inhabitants, the prevalence of HCV infection in general population ranges from 3 to 5% (1)(2)(3)(4). A recent cross-sectional survey conducted by the University of Health Sciences (UHS) in four diverse geographical areas including the capital city of Phnom Penh and three provinces (Battambang, Siem Reap and Preah Sihanouk) reports that among 425 people living with HIV (PLHIV), prevalence of positive HCV Ab was 6.8% and prevalence of positive HCV RNA was 5.4% (Pheng P, Poster MOPEA0090 at IAS2017, Paris). Since the 90s, Cambodian government implemented a large access to systematic screening of blood donation for HBV and HCV and generalization of single use syringes. This latter action is particularly important because HCV transmission is mainly linked to parenteral infusions, surgery or endoscopy before years 2000 (5) and the most at risk population seems to be patients aged above 40 years (5)(6). In this population, prevalence of HCV infection increases with age and could reach 10 to 15% for persons aged more than 50 years old (MSF report). Systematic screening of HCV infection is not performed in Cambodia, and few patients with chronic HCV infections are aware of their status. Serological tests (ELISA and rapid tests) are available in public and private facilities while virological testing is limited to some laboratories in Phnom Penh. Predominant genotypes are genotypes 1 and 6 for more than 90% of patients (6)(7).

The treatment and care of HCV-infected patients are predominantly provided in the private sector and in 2 public hospitals (Kossamak hospital in Phnom Penh and Mong Reussey Referral hospital, Batambang province) within the MSF cohort. The use of Transient Elastography (Fibroscan) to assess liver fibrosis is rare as only two hospitals and one clinic in Phnom Penh are equipped. Patients with advanced stage of disease are referred to public hospitals. Hepato Cellular Carcinoma (HCC) is the first cause of cancer for men in Cambodia (8) and mainly linked to HBV and HCV infections(9)(10). Cirrhosis complications related to HCV seem also to be a major reason of hospitalization in public hospitals, but no precise data are available. Effectiveness of DAA within the MSF cohort was reported in 2 workshops: SVR12 was above 95% with the sofosbuvir/daclatasvir combination during 12 weeks.

The national HIV/HCV coinfection guidelines launched in April 2017 and training was completed in 6 provinces: Banteay Meanchey, Battambang, Kampong Cham, Phnom Penh, Siem Reap, and Tbaung Khmum. To date, 11,393 patients have been screened which represent 42.1% of the ART cohort at 6 selected provinces, 560 are positive for HCV Ab (4.9%) and 372 patients have received confirmatory viral load tests result (262 positive, 70%). NCHADS plan to continue screening to reach 42000 patients screened at the end of 2018. Regarding these preliminary results (3.5% of positive HCV RNA), approximatively 1500 patients will be treated with DAA.

Ministry of Health has implemented a working group on the thematic of viral hepatitis B and C infections within the CDC (Communicable Disease Control) department. The National Strategic Plan (NSP) for Viral Hepatitis (VH) 2020-2024 has been developed during a series of multi-stakeholder consultations from 2017 to July 2019. This is the first NSP-VH 2020-2024 and is aligned with the National Health Services Strategic Plan 2016-2020. The goals are towards the elimination of viral hepatitis C as a public health threat by 2030, based on clear public health approach that promotes the rational, cost effective and safe use of medicines at the operational district level. Successively, the National Guidelines for management of viral hepatitis B and C

infections has been developed during a series of multi-stakeholder consultations from July to December 2019.

Operational researches are also needed to determine where the funds must be allocated the most efficiently. Interventions focused on the cascade of care seem the most appropriate measure and targeting general population aged more than 40 years old seems a priority in Cambodia as they cumulate higher level of prevalence and higher risk of advanced liver diseases.

Cascade of care for HCV infection in LMICs

Availability of DAA, with cure rates approaching 90 to 95% along with their good tolerance, is expected to change the dynamic of HCV infection worldwide and give hope of eradication. However, the impact of DAA in the general population is completely related to the population-level cascade of care: screening programs must reach undiagnosed individuals, diagnosed individuals must be linked with care and people engaged with care must be assessed, receive treatment and be cured. In 2016 in the Western Pacific region, according to WHO, only 21% of people living with HCV have been diagnosed and 2% started treatment. There is a need to address how different interventions apply to the context of well-tolerated, simple, oral treatment regimens. The Georgia HCV elimination program has recently reported promising results (11) but the authors highlight that "high-quality screening, innovative linkage-to-care strategies, and cost-effective and simplified diagnostic and treatment regimens are needed » to reach the elimination target.

Testing strategy

The question of "who to test" and "how to test" in LMICs was discussed in a recent article (12). WHO recommended « that HCV serology testing be offered to individuals who are part of a population with high HCV seroprevalence or who have a history of HCV risk exposure/ behavior » (13). This recommendation includes « persons who have received medical or dental interventions in health-care settings where infection control practices are substandard » which was the case of Cambodia before years 2000. Targeted HCV testing interventions to individuals who are a part of risk groups for HCV infection or who have a history of HCV risk behaviors seems to be effective to diagnose cases and increase treatment uptake (14) but the reported programs are mainly heterogenic, and the majority did not use a comparison group (15). However, many countries in Europe and US recommend a systematic population-based screening for groups at risk of infection. In US, excepting classic at-risk groups (injecting drug use, hemodialysis, and prisoners), HCV infection is most prevalent among individuals born between 1945 and 1965 (baby-boomer population) (U.S. Department Of Health And Human Services/Centers for Disease Control and Prevention (CDC). HEPATITIS C: Why Baby Boomers Should Get Tested). Since 2012, CDC recommends HCV screening in this population. In France, recommendation changed in 2014: HCV screening must be done for all men aged 18-60, with also a particular warning for the baby-boomer population, as many persons are still undiagnosed in 2014 in this population(16). Screening could be done in different medical structures (17)(18)(19)(20) and could easily be integrated in routine primary care (21).

Nevertheless, all these studies were conducted in high-income countries and data in low-income countries are scarce. Rapid diagnosis tests are available in the majority of these countries but targeted testing interventions are related to country's epidemic situation and to the health care structures. Community approach with testing at a household level was reported effective in Egypt

to ensure both high uptake of testing, and equity in access regardless of sex, age, income level, or stage of disease (22). As recommended by WHO, « Operational research is needed to evaluate different approaches to increase the reach and uptake of screening services ».

However, a comprehensive and integrated approach to the elimination of HCV at a community level, must incorporate both preventive approaches to reduce transmission and new infections, and testing and treatment to reduce both the burden of disease and associated morbidity. A prompt linkage to care to ensure access to treatment is essential. In the Egyptian publication, the treatment coverage and cure were over 90% of the estimated HCV-infected village population.

HCV RNA quantification

WHO suggest that nucleic acid testing for the detection of HCV RNA be performed directly following a positive HCV serological test to establish the diagnosis of chronic HCV infection and that all patients with positive HCV RNA should be assessed for the treatment.

HCV RNA availability in resource-limited setting is challenging. HCV RNA could be done on plasma but requires syringes, tubes, centrifuges machines and skilled human resources. The Cepheid Xpert HCV viral load assay could be an alternative to central laboratory equipped for classical virological diagnosis (23)(24). The limited operator input allows considering an implementation in provincial hospital and a recent article report a good accuracy on finger-stick capillary whole blood (25). However, patient needs to come to provincial hospital, which could increase the risk of lost-to-follow-up after a positive Ab. And the total number of possible samples evaluated each day is limited, which could be challenging for a scale-up strategy.

The performance of the Omunis Generic HCV assay on an open polyvalent PCR platform was recently evaluated in France and Cambodia (26); this HCV PCR assay showed good concordances in clinical specimens when compared to the Roche assay. The cost per test of the Generic HCV assay has been estimated by MSF Access Campaign at $22.6 including the Arrow automated extraction kit and proteinase K reagent, which is close to the cost per test of the Cepheid Xpert HCV viral load assay estimated at $18 (27). This affordable HCV test could represent a surrogate method to manufacturer's PCR systems for the diagnosis and monitoring of HCV RNA testing in central laboratories from low- and middle- income countries. It could allow 40 to 82 samples per day and could be used on plasma or DBS (27). DBS could represent an alternative to simplify and decentralize access to HCV RNA, specifically at community level. DBS could easily be sent to equipped central laboratory and HCV RNA performed at this level. The performance seems good but sensitivity seems altered for patients with low-level viremia (28) and HCV RNA degradation could occurred in DBS samples stored at room temperature (29).

Linkage to care and access to treatment

Specific HCV services with nurse educational interventions and access to treatment increase retention in care, treatment completion and cure (30). Considering the high chance of cure for HCV infection after DAA treatment, reaching elimination target seems to be a non-utopian option if interventions to improve the cascade of care are implemented (31) and some countries, as Georgia, have implemented national programs to eliminate HCV infection with access to DAA whatever the fibrosis assessment (11).

Many DAA combinations has proved to be effective in Asia (32) but cost and affordability remains the major issue. In Cambodia, genotypes 1 and 6 represent almost 95% of HCV infections and genotype 2 is reported in 5% of cases (6)(7). Association of sofosbuvir and daclatasvir was reported to be effective for these 2 genotypes (33)(34) but also recently for genotype 3 (35) and is affordable at low price in the country. In a public health approach, it could be cost-effective to treat all the patients as it was recently reported in Korea (36).

Health care system organization in Cambodia

Private practitioners and clinics are particularly frequented for curative care, whereas health promotion and prevention activities are the domain of the public sector. The low utilization of the public health facilities for curative care remains a concern. Only 29% of unwell or injured patients sought care first in the public sector, while 57% sought care for their last episode at private providers, according to the 2010 Cambodian Demographic and Health Survey.

Public health services are available through a national network of Health Centers and Referral Hospitals; in principle, primary care services are available within two hours walk from home for the whole population:

• Health Centers provide maternal, neonatal and child health services, including immunization, nutritional education, screening for breast and cervical cancer, safe abortion; treatment and prevention of communicable diseases; treatment and prevention of NCDs (Non Communicable Diseases) and injuries; and through outreach activities.

• District Referral Hospitals provide outpatient care as well as inpatient treatment for referred cases, complicated tuberculosis cases, medical, surgical and obstetrical emergency cases, some surgery, maternal and child health services, provision of X-ray, ultrasound and laboratory services, and rehabilitation services.

However, many patients go straight to hospitals, bypassing the health centers.

Provincial Health Departments (PHD) operate a provincial hospital and cover from one to 10 Operational Districts (OD). Each OD covers a population of 100 000–200 000 with at least one Referral Hospital (RH) and a number of Health Centers (HC) that each cover 10 000–20 000 people.

As part of health system development, in 2003 (revised in 2008), the Community Participation Policy for Health was created and implemented, highlighting a key role for community health workers (CHWs) (37). Programs implementation are financed and supported by Provincial Health Departments (PHD) and the administrative Operational District (OD) in terms of structure and management. Each CHW is associated with a health centre where they could receive training and supervision and in some cases resources to support their role. The policy states that CHWs should be literate, live in the communities they serve and be elected by community members. Each CHW serves between 10 and 50 households depending on the community need. The CHWs are not a homogeneous group and vary widely in age, gender, experience, social status and political affiliation. Their roles include responsibility to analyze and identify health issues arising in the community and communicate these to health centres and to share information from health centres with the community. However, they face many challenges as lack of financial support, lack of training and lack of community interest and participation (38).

Study main hypothesis

- Comparative testing interventions is a prerequisite to improve the cascade of care
- Population aged more than 40 years old must be targeted in priority
- Testing interventions must include serological and virological tools as a package to improve active case detection
- Community approach using RDT/DBS combination could increase the testing uptake and the active case detection compared to facility approach
- The relevance of a systematic treatment is fully realized for all patients with active infection, most at risk of severe fibrosis and liver cancer. This strategy will not be compared and implemented in the 2 arms.

4. OBJECTIVES OF THE STUDY

Primary objective

The primary objective of the study is to compare the effectiveness of a community-based intervention to a facility-based intervention to improve the combined-testing uptake (Antibody + RNA) of HCV infection among general population aged more than 40 years old in Cambodia

Secondary objectives

- To compare the HCV antibody testing uptake between the 2 arms for the eligible population
- To compare the active case detection rate between the 2 arms for the eligible population
- To compare the linkage to care between the 2 arms for those with active infection
- To compare the cost-effectiveness of the two strategies
- To evaluate the treatment uptake
- To evaluate the effectiveness and safety of a 12-week dual-therapy of direct-acting antiviral (DAA) treatment

5. METHODOLOGY

Study design

This is a two-arms cluster-randomized controlled trial, in which clusters are defined as a group of 50 households corresponding to the service population of the CHWs.

The study will be conducted in 2 different provinces.

Study overview

A facility-based testing intervention (group 1) will be compared to a community-based intervention (group 2). The study will be conducted in 2 separated provinces: Siem Reap and Kampong Cham provinces. Each province has approximatively one million of inhabitants and one

provincial hospital. Siem Reap province is composed of 4 ODs, 103 communes, 5 RHs and 88 HCs. Kampong Cham province is composed of 9 ODs, 107 communes, 9 RHs and 98 HCs.

For the study, one OD in Siem Reap province and 2 ODs in Kompong Cham province with approximately the same number of villages and inhabitants and the same distance from PHD will be selected. The districts are sub-divided into communes. Communes are further divided into villages. Villages are sub-divided into groups. A group is generally a combination of 50 households and one group will be defined as one cluster.

In order to decrease the risk of contamination between clusters, in each province, 4 geographic area, separated by communes not involved in the study, will be selected. A random selection of the geographic area in each province will be done to be part of arm 1 or 2.

For each geographic area, a multistage cluster sampling method will be used to select study participants:

1- A Probability proportional to size (PPS) sampling method to select 10 villages in each selected GA will be realized in a first step

2- Then, a preliminary mission will be organized in order to identify and confirm the location of HHs in the 10 selected villages. During this mission, information on age of inhabitants will be collected with the heads of village and a list of HHs will be identified

3- A Simple random sampling (SRS) method will be realized to select 100 households per village within the 10 selected villages

4- In each selected household, all persons aged more than 40 years old will be proposed to be part of the study.

Tentative study agenda

First inclusion: May 2022

Inclusion time: 6 months.

Time of follow-up for each participant: 6 months

Last visit for the last patient: April 2023

6. STUDY POPULATION

Inclusion criteria

- All persons aged more than 40 years old
- Residing in the study area
- Informed consent obtained with oral information given and explained and the consent form signed by the participant and the nurse hired by the study at the latest the time of the RDT realization

Non-inclusion criteria

- Known HCV status with previous HCV treatment
- Severe disease present at inclusion involving life threatening

- Concurrent participation in any other clinical study without written agreement of the two study teams

7. STUDY ENDPOINTS

Primary endpoint

The primary endpoint is the **combined-testing uptake** defined as the number of persons tested for HCV RDT AND HCV RNA and aware of their status among the total number of persons eligible residing in the region where the intervention takes place

Secondary endpoints

The following secondary endpoints will be measured and compared between the 2 arms:

- **HCV antibody testing uptake** defined as the number of persons tested for HCV RDT and aware of their status among the total number of persons eligible residing in the region where the intervention takes place
- **Active case detection rate** defined as the number of persons with HCV active infection (positive HCV Ab and positive HCV RNA) and results given and explained among the total number of persons eligible residing in the region where the intervention takes place
- **Linkage to care** defined as the number of persons with at least one consultation in the Provincial Hospital among the estimated total number of persons with active infection residing in the region where the intervention takes place

The following secondary endpoints will be measured for the total population and not compared:

- **Treatment uptake** defined as the number of people initiating HCV treatment among the total number of persons with active infection linked to care
- **Liver-related morbidity and mortality** (decompensated cirrhosis, HCC)
- Proportion of patients with sustained virologic response 12 weeks after discontinuation of treatment (**SVR12**)
- Proportion of patients with **treatment failure** defined as absence of SVR12 or missing HCV-RNA at 12 weeks post-treatment (PT12) due to treatment discontinuation for AEs or death

8. STRATEGIES AND TREATMENTS

Strategies

Arm 1: Facility-based testing intervention

Community Health Workers (CHWs) will provide information on the possibility to be tested in health centers for HCV infection. Information will be provided using information sheet and a dedicated leaflet in the selected households. If the participant agrees to participate, she/he will receive a voucher to go to referral health center for testing. In health center, after signature of

consent form, HCV testing will be done using HCV RDT on a finger stick capillary whole blood. Results will be available in 15 minutes. In case of positive HCV RDT, an immediate blood sample collection (5 mL of whole blood on EDTA) will be done in health center and sent to Provincial hospital laboratory to collect plasma. Plasma will be sent to UHS to perform HCV RNA. Results will be sent back to the health center and nurses will be in charge to give result to the participant and to refer to care in case of active infection (systematic appointment + phone call if the participant doesn't come).

Arm 2: Community-based testing intervention

After a dedicated training, CHWs will do the HCV RDT on a finger stick capillary whole blood directly in the household of participant. Information will be provided by the CHW using information sheet and a dedicated leaflet. If the participant agrees to participate, the signed consent form will be collected at this step before any RDT collection. In case of structural or societal barriers for household testing, it will be possible to provide testing in a specific location in the village. The visits will be planned with the head of village and he will be in charge to inform the population. In case of absence of participant during the first visit, a second visit will be scheduled. In absence of participant during the second visit, no additional visit will be planned.

In case of positive HCV RDT, 5 blood spots will be collected immediately on DBS, put in a specific plastic bag with the ID number and sent to UHS (Rodolphe Merieux laboratory) for HCV RNA extraction and amplification. Results will be sent back to the referral health center of each village and nurses will be in charge to give result to the participant and to refer to care in case of active infection (systematic appointment + phone call if the participant doesn't come).

Patients with a known HCV positive status

Patients with a known HCV positive status will be tested by RDT to confirm HCV infection and will be followed according to the procedure described above. Patients with a prior history of HCV treatment could not be enrolled in the study.

Common management in the provincial hospital for positive HCV RNA patients

For negative HCV RNA, a specific counseling will be done.

For positive HCV RNA, a consultation in the Provincial Hospital will be planned. In the absence of consultation within 4 weeks, SMS reminder will be sent to participant. A correlation table will be available in the provincial hospital to confirm the identification of the participant. Trained general practitioners will do the consultation with the possible support of hepatologist by phone. The baseline assessment will include questionnaires (risk behaviours and socio-economic status), clinical exam, blood sampling and liver ultrasound.

Symptomatic cirrhotic patients will be defined as patients with at least one of these signs: ascites, collateral circulation of the abdominal wall around the umbilicus, encephalopathy, jaundice, HCC. These patients will be referred to a National Hospital in Phnom Penh to a hepatology unit.

For the others patients, DAA treatment will be proposed, after checking the result of creatininemia and the possible drug-drug interactions.

Treatments used in the study

None of the drugs used in the study is experimental.

Combination of daily sofosbuvir (400 mg) and daily daclatasvir (60 mg) will be provided to patients for a total duration of 12 weeks. DAA treatment will be introduced at the first consultation in the provincial hospital with a monthly follow-up. WHO pre-qualified Generic drugs (Hetero labs limited) will be used. The dose will be Sofosbuvir 400 mg one tablet per day and Daclatasvir 60 mg one tablet per day.

<u>Supply</u>

All investigational medicinal products will be sourced from Good Manufacturing Practices (GMP) certified manufacturers. The Monitoring and Data Management Center (MDMC) will centralize the procurement of these products and ensure access to these drugs to Provincial hospitals for the total duration of the study.

<u>Packaging and labelling of IMP</u>

In this study, investigational medicinal products will be prepared, packed and labelled in accordance with GMP (international conference of harmonization (ICH) guide for active pharmaceutical ingredients Q7,2000, http://www.gmp-compliance.org/guidemgr/files/3-1-18.PDF). A specific study label will be added to each drug bottle in compliance with the GMP and Cambodian laws and regulations. The label templates will be validated by the MDMC and the sponsor.

<u>Handling and storage</u>

The IMP's will be delivered by supplier to the University of Health Sciences at the beginning of the study and eventually during the study. The MDMC will store the IMP's at room temperature in a locked environment. The responsible pharmacist or investigator will maintain a temperature control log and medication inventory. Details on handling, shipment and storage of study drugs will be specified in a separate document (study specific standard operating procedures).

<u>Responsibilities</u>

In compliance with ICH Good Clinical Practices (GCP), the responsibility for the IMPs at the study site lies with the investigator and pharmacist and includes the following:

- Receipt of the study medication;
- Documentation of deliveries;
- Adequate management, storage and destruction of study medications;
- Keeping of records including dates, quantities and the batch numbers / expiry date assigned to the investigational medication and ID study subjects;
- Documentation including the dispensation of the study medications to subjects as specified by the protocol;
- Reconciliation of all investigational medications.

The following guidelines apply:

- The investigator agrees not to supply any medication to any person except to the patients included in the study;
- The pharmacist agrees to keep the medications in a locked, secure, storage facility, only accessible to authorized staff;
- A study medications inventory is maintained, including materials received and dispensed to patients;
- During the study, the pharmacist agrees to conduct a drug supply inventory and to record this on a Drug Accountability form.

The supply and handling of other drugs than IMPs provided to participants for their care will be performed according to the usual procedures of the study sites.

Accountability

Every time medication is dispensed, the pharmacist will record the study subject identification code, the name of the drug, the batch number, the expiry date and the accountability on the Drug Accountability form. Patients will be asked to bring back bottles of used or left-over drugs. The Drug Accountability form will be checked by the CRA during on site monitoring.

Once accountability has been checked by the study monitor, during the study and at the end of the study, any remaining drugs brought back by the patient will be destroyed by the pharmacy of the trial site, according to each country's regulations.

Treatment follow-up

Monthly consultation and treatment delivery will be applied. All adverse events will be assessed by the investigator and documented regardless of the possible causality with the concomitant treatments. Adherence will be evaluated every month using questionnaire and visual analog scale. Biological follow-up during the treatment is reported in table 1.

Treatment will not be initiated in the following situation:

- Indication to palliative care
- Hepatocellular carcinoma (HCC) diffuse with infiltration
- Hepato-renal syndrome type 1
- Severe renal insufficiency defined as a creatinine clearance < 30 ml/mn
- Active pregnancy
- Breastfeeding
- Contraindication for treatment with sofosbuvir or daclatasvir
- Hypersensitivity to the active substance or to any excipients
- Any condition which might, in the investigator's opinion, compromise the safety of the patient including very severe clinical condition

Patients with cirrhosis

Asymptomatic cirrhotic patients will be treated as the other patients and followed in the Provincial Hospital with sofosbuvir/daclatasvir combination for 12 weeks.

Symptomatic cirrhotic patients will be referred to National Hospital and treatment will be discussed case by case. Additional exams will be funded by the study. In case of difficult situation, a Decision Support Committee will help the physicians to take a decision.

Patients with HIV co-infection

- If HIV infection is known, ART-treated with a HIV RNA VL < 1000 copies/mL, patient could be treated with DAA considering the possible drug-drug interaction between ART and DAA. A reduced DCV dose (30 mg daily) will be recommended for patients receiving ritonavir-boosted atazanavir or other potent inhibitors of cytochrome P450 3A4 (CYP3A4) or P-glycoprotein, and a dose increase (90 mg) recommended with efavirenz or other moderate inducers of CYP3A4.

- If HIV infection is just diagnosed or not virologically controlled, patient will be addressed to the referral ART site to receive specific care before any intervention for HCV infection.

Patients with HBV co-infection

All patients with HCV active infection will be tested for HBsAg at inclusion. If HBsAg is positive, a treatment by tenofovir will be initiated from inclusion until 12 weeks after discontinuation of HCV treatment.

In case of sudden rising of ALT level during follow-up of HCV treatment, HBV DNA will be performed to eliminate reactivation of occult HBV infection and a treatment by tenofovir could be initiated in case of positive result.

Treatments used

Sofosbuvir: 400 mg once daily

Daclatasvir: 60 mg once daily

Treatments that could not be used or use with precaution because of potential drug-drug interactions

Refer to the respective SmPC daclatasvir and sofosbuvir in Appendix 7 and 8 for drug interaction information in medicinal products and Appendix 9 for the main drug-drug interactions.

9. STUDY IMPLEMENTATION

Study schedule

	Testing phase	Therapeutic phase for positive HCV RNA	W4	W8	W12	W24	W28
Consent form	X	X					
Eligibility criteria	X	x					
Consultation and clinical exam		x	x	x	x	x	x
HIV RDT		x					
HBs Ag RDT		x					
HCV Ab RDT	X						
HCV RNA plasma	x [1]					x	
HCV RNA DBS	x [2]						
AST / ALT		x	x	x	x	x	
Creatinine		x	x	x	x		
CBC		x	x			x	
Pregnancy test[4]		x	x	x			
PT		x				x[3]	
Bilirubin		x				x[3]	
Albumin		x				x[3]	
Fasting glycemia		x					
Score APRI / FIB4		x					
Liver ultra-sound		x				x[3]	
Biobank		x			x	x	
Socio-economic evaluation	x						
DAA delivery		x	x	x			
Adherence assessment			x	x	x		

(1) For patients in arm 1

(2) For patients in arm 2

(3) For cirrhotic patients

(4) Pregnancy test will be performed at inclusion, W4 and W8 for all women of childbearing age

Patients' participation in the study

Information

Patient participation in this clinical study is voluntary.

Each potential participant will be informed individually of the purpose, scope of the study, procedures involved, duration of follow-up, potential risks and benefits and any discomfort it may entail. In addition to oral explanations, a written information sheet will be systematically provided in Cambodian language (see Appendix A).

Consent

If the patient accepts to be enrolled in the study, he will write his/her full names clearly, and date and sign the consent form, as will do the investigator (the nurse or the CHW). Written informed consent must be obtained before any exam related to the study. The original copy of the form will be stored in a location that is secure and inaccessible to other clinic employees. Participants may be given, if they wish, a copy of the consent form after they sign.

Consent will be collected in health centres for Arm 1 and directly in the household of participants for Arm 2.

A second specific information sheet and consent form will be used for the therapeutic phase. Consent will be collected in the provincial hospital.

Information process for Arm 1

- The CHWs will be in charge to inform participants in their respective households:
  - o Provide information on the study using information sheet and leaflet
  - o Distribute dedicated voucher including serial number code. These vouchers will be used in HC as identification documents for nurses. One part will be given to participant and one part will be kept by the CHW and stored in health centres

Testing phase for Arm 1

The testing phase for arm 1 will take place in health center. During this visit, the investigator:

- Check to confirm that 1/ the participant is in the list of selected HH (2) the number on the participant's voucher match with the second part.
- Check eligibility criteria
- Collect the written consent form
- Attribute a identification code in the study
- Complete the confidential list including personal information (address, phone number) and keep it in a secured cupboard in the health center
- Fill the screening form
- Perform the HCV RDT on a finger stick capillary whole blood
- Give the result to the participant
- Provide specific counseling to avoid future transmission in case of negative result

- Collect blood sample (5 mL EDTA tube) in case of positive RDT and send it to Provincial hospital laboratory to collect plasma. Plasma will be sent to UHS with the ID number to perform HCV RNA
- Give an appointment to participant to receive the result of HCV RNA

Testing phase for Arm 2

The testing phase for arm 2 will take place directly in the household of participant or in a specific location in the village. During this visit, the CHW:

- Check eligibility criteria
- Provide oral information, information sheet
- Collect the written consent form and give an ID number
- Complete the confidential list including personal information (address, phone number) and keep it in a secured cupboard
- Fill the screening form
- Perform the HCV RDT on a finger stick capillary whole blood
- Give the result to the participant
- Provide specific counseling to avoid future transmission in case of negative result
- Collect DBS (5 spots) on a finger stick capillary whole blood in case of positive RDT and put it in a specific plastic bag with the ID number
- Keep the DBS in HC in the fridge and send to UHS on a regular basis
- Give an appointment to participant to receive the result of HCV RNA

Case detection phase

The results of HCV RNA will be centralized in HCs and nurses will be in charge to give the results.

During this visit, the nurse confirms the identification of the participant and informs the patient of the result of HCV RNA. The nurse will be in charge to confirm the awareness of testing status and to fill the CRF.

<u>For negative HCV RNA patients</u>

The nurse will be in charge to explain to the patient that HCV infection has occurred and is cured and don't need any follow-up and exams. The most important point is to explain to the patient that HCV Ab will stay positive for all life without any consequences for him. But re-infection is possible.

<u>For positive HCV RNA patients</u>

The nurse gives and explains the result to the participant and plan a visit in the Provincial Hospital in a short delay for liver disease assessment and treatment.

Liver disease assessment and treatment phase

<u>First visit in the Provincial Hospital (W0)</u>

- Collect clinical data including:

  - Socio-demographic: Gender, birth date, Ethnic origin, Profession, family situation, Level of income of the household, Contamination way, Liver diseases in the family
  - Drug abuse: Binge drinking, Alcohol dependence estimated by FACE questionnaire, Drug consumption and dependence
  - Anamnesis: Familial history of liver cancer, Personal history of liver disease, Vaccination HBV, Received Hepatitis C treatment in the past, was diagnosed as diabetics, Cardiovascular events, Current traditional medicine treatment
  - Co-morbidity: Metabolic syndrome, HIV infection, HBV infection,
  - Clinical examination: Weight, Height, BMI, Blood pressure, Proteinuria, Hepatic enlargement, Splenic enlargement, Ascites, Collateral circulation of the abdominal wall around the umbilicus, Encephalopathy, Ankle's edema, Extra hepatic signs

- Perform HIV and HBsAg RDTs
- Collect biological samples and ask creatinine in emergency to get the result in one hour
- Collect urine pregnancy test for women of childbearing age
- Assess fibrosis level: APRI Score, Fibrosis-4 score (FIB-4). CHILD score for cirrhotic patients
- Perform liver ultrasound: echostructure, Liver size, capsular contours, Liver nodules, Spleen size, Flow in the portal vein, Portosystemic shunt, Ascites
- Explain and propose DAA treatment for non-symptomatic cirrhotic patients: dose, possible side effects, adherence
- Give the prescription to the patient(monthly) and plan the next visit
- Refer symptomatic cirrhotic patients to national hospital

Follow-up visit (from W0 to W12)

From W0, the follow-up visit will take place at W4, W8 and W12.

During these visits, all patients will receive a clinical exam, an adherence counseling and an evaluation of adverse events (graded according to ANRS scale)

A blood sample will be done at W4 including complete blood count, AST/ALT and creatininemia.

A blood sample will be done at W8 and W12 including AST/ALT and creatininemia.

Urine pregnancy test for women of childbearing age will be done at W4 and W8.

Visit W24

HCV RNA will be performed to evaluate the SVR12. In case of cirrhosis, CBC, PT, bilirubin, albumin will be done to evaluate the Child score and liver ultrasound will be performed to screen HCC.

Visit W28

This visit will be the last visit of the study. In case of SVR12, patients will be informed of the cure. Recommendations to continue liver ultrasound follow-up every 6 months will be given for cirrhotic patients.

Procedure for non-responder's patients

In case of treatment failure, patients will be referred to the National Hospital in Phnom Penh. HCV genotype and genome sequencing will be done in IPC and a Transient Elastography will be performed. A dedicated committee will evaluate the results and give a decision for therapeutic options.

Communication of study results to the participants

As soon as final results of the study are available, they will be presented, both orally and in writing, to:

- The investigators;
- The national authorities;
- The participants.

A series of documents (written detailed report, and short summary) will be released to help investigators, national authorities and participants to understand the results of the study. Participants may be invited to attend a meeting during which the results will be presented and explained orally.

Permanent study treatment discontinuation

Reasons for permanent discontinuation of study treatment include:

- Willingness of the patient;
- Any medical event requiring discontinuation of the study treatment (drug-intolerance or occurrence of Serious Adverse Events (SAE), occurrence or worsening of a concomitant illness);
- Need for the patient to be treated with a non-authorized concomitant medication;
- Death of the patient;
- Non-adherence to the study treatment endangering the patient's health.

If a patient wishes to discontinue study treatment at any moment, investigators should explain the importance of remaining on study follow-up, or - failing this - of allowing routine follow-up data to be used for study purposes.

A premature discontinuation of study treatment must be notified by e-mail to the clinical study manager of the CTU as soon as the investigator is aware and reported in the patient CRF. The reasons and date of the discontinuation should be documented in the patient's medical file. Study treatment discontinuation should not lead to patient withdrawal from the study: patients discontinuing their study regimen have to be followed-up according to the scheduled study visits until the end of the study and all data should be collected (under the condition that informed consent has not been withdrawn).They should receive the best possible care given their health status and current best knowledge.

Withdrawal of consent and loss to follow-up

Consent withdrawal

A patient will be considered to have *withdrawn consent* if s/he no longer wishes to remain in the study. In this case, a "*withdrawal of consent form*" must be filled out.

Loss to follow-up

When a patient who has not explicitly withdrawn consent does not show up for routine visits, the study team (nurses, counsellors…) will search for him/her by all the way available (phone call, mailing, home-based care team). The study team will contact the patient via phone call or home visit, as long as s/he consented to phone calls and/or home visits in the consent form and provided a phone number and/or home address during the study.

A patient who does not show up for a given scheduled visit will be considered lost-to-follow-up when his/her last contact with the study team (either at the clinic, via telephone, or at home) was recorded prior to the date of his/her last scheduled visit to the clinic as per the study protocol.

Cost-effectiveness analysis

The objective of the cost-effectiveness analysis in this project is to evaluate the individual and societal benefit of a mobile HCV testing service strategy compared to a communication strategy to motivate people for being tested in health centers. The cost-effectiveness analysis aims the comparison of two different public health combined-testing strategies, which raises important methodological challenges. In order to reduce the impact of assumptions of the model on both the results and the conclusions, a special attention will be paid to the collection of the price of DAAs, the analysis perspective, the time horizon and the characteristics of other HCV-related diseases. Our analyses will also account for other costs such as non-medical costs (wage loss, transportation costs, etc.).

For the cost-effectiveness analysis, the following costs will be estimated

Costs specific to each arm

Arm 1: Facility-based screening intervention

- Costs of providing information in the community, time spent/person, salary/person
- Cost of HCV RDTs
- Costs of blood sample: human resources and material needed for

- Cost of blood samples conditioning and transfer between facilities, the Provincial hospital and Phnom Penh: per unit

Arm 2: Community-based screening intervention
- Costs of CHWs mobilization: time spent/person, salary/person
- Cost of HCV RDTs
- Cots of blood samples on DBS: human resources and material needed for
- Cost of blood samples conditioning and transfer towards Phnom Penh: per unit
- Cost of DBS analysis: per unit

Costs to be collected irrespective of the arm <u>per patient</u> and during the <u>whole follow-up</u>

- Complementary biological tests necessary for treatment initiation or follow-up: number of patients, type of test and patient-specific cost, human resources and inputs (i.e. for blood sampling)
- DAA treatment: number of days on treatment and cost of treatment
- Concomitant drugs used: number of days on treatment and cost of treatment
- Doctors' outpatient consultations: number of patients & cost of a consultation
- Nurses' or CHW outpatient consultations
- Inpatient care admissions: number of days per patient, cost of a day

For cirrhotic patients:
- Complementary biological tests and liver ultrasound: number of patients, type of test and patient-specific cost, human resources and inputs (i.e. for blood sampling)

For non-responders:
- Cost of HCV genotype
- Cost of genome sequencing
- Cost of Transient elastography
- Cost of human resources and inputs related to non-responders care before referral to the National hospital.

10. LABORATORY EVALUATIONS

Blood samples collection and tests

Blood samples will be taken:

(i) at screening phase, to measure HCV RDT and HCV RNA

(ii) at DAA initiation for positive HCV RNA patients, to measure HBsAg and HIV RDT, complete blood count, serum transaminases, serum creatinine, fasting glycemia, prothrombin time, bilirubin, albumin,

(iii) at week 4, to measure complete blood count, serum transaminases and serum creatinine,

(iv) at week 8 and week 12, to measure serum transaminases and serum creatinine,

(v) at week 24, to measure complete blood count, serum transaminases, serum creatinine, plasma HCV-RNA viral load;

	**test**	**DAA**	**W4**	**W8**	**W12**	**W24**
Complete blood count: EDTA (mL)		2	2			2
Biochemical analysis: Plasma, plain tube (mL)		5	5	5	5	5
Coagulation profile: Plasma citrate (mL)		2				
Plasma HCV RNA: EDTA tube (mL) or DBS	5					5
Biobank: EDTA (mL)		5			5	5
Total number of tubes at each visit	**1**	**4**	**2**	**1**	**2**	**4**
Quantity of blood at each visit (mL)	**5**	**14**	**7**	**5**	**10**	**17**

HCV RNA viral load

In the arm 1, HCV RNA will be done using the Biocentric Generic HCV assay on plasma. Five mL of whole blood will be collected on EDTA tube. To avoid the risk of HCV RNA degradation during the transportation, whole blood tubes will be sent to Provincial Hospital laboratory first to collect plasma. Then, plasma tube will be sent to the UHS laboratory to perform HCV RNA.

In the arm 2, blood will be collected on DBS. DBS will be packaged in sealable plastic bags with a desiccant until transported to health center. In health center, packaged DBS will be kept in fridge. One time per week, the head of HC or nurse will insert bundled DBS into rip-resistant envelope and give to taxi for transportation to UHS in Phnom Penh. The HCV RNA will be eluted from DBS using DBS elution buffer (PBS, BSA 10%, Tween 0,05%) and manually extracted using the Qiamp Viral RNA Mini kit then amplifies using the Biocentric Generic HCV assay on the BioRad CFX96 thermocycler.

All the samples collected during the screening phase will be destroyed after HCV RNA completion.

Biobank

During the therapeutic follow-up, blood samples will be collected for all patients and frozen at week 0, 12 and 24.

Biobank will be centralized at UHS, in Rodolphe Merieux laboratory and preparation of the aliquots will be done at this location. Plasma collected from each 5 mL blood sample will be divided in 2 mL of plasma frozen in 2 aliquots of 1 mL each. A specific database will be used to

register the samples. All the samples will be kept frozen for a period of 15 years after agreement of participant.

11. Clinical SAFETY

11.1. Definition

11.1.1. Adverse Event (AE)

Any untoward medical occurrence in a clinical trial participant, which does not necessarily have a causal relationship with the research or the study product.

An adverse events include:

- Any increase in frequency or intensity of an event or pre-existing condition
- Any condition (even if it was present before the start of the trial) detected after the subject signed the informed consent

An adverse events does not include:

- A medical or surgical procedure (only the condition that led to such a procedure is an adverse event)

11.1.2. Adverse Reaction (AR)

All untoward and unintended responses to an investigational medicinal product related to any dose administered.

11.1.3. Serious Adverse Event/Reaction (SAE/SAR)

A serious adverse event/reaction refers to any untoward medical occurrence or reaction that at any dose:

- results in death;
- is life-threatening*;
- requires hospitalization or prolongation of existing hospitalization**;
- results in persistent or significant disability or incapacity;
- is a congenital anomaly or birth defect;
- is an "important medical event" including adverse event of special interest***

*means that the subject was at immediate risk of death at the time of the event; it does not refer to an event which hypothetically might have caused death if it were more severe.

** hospitalization is defined as admission to hospital, regardless of the length of stay, even if hospitalization is a precautionary measure allowing continued observation.

<u>Exceptions:</u>

Do not constitute a serious adverse event:

- Hospitalization for a pre-existing condition that has not worsened, scheduled before inclusion in the trial
- Hospitalization without harmful or unwanted manifestation (hospitalization for administrative or social reasons, elective hospitalization for medical or surgical treatment, hospitalization predefined by the protocol, etc.)

***the investigator's medical opinion can decide whether an event can be qualified as serious. The following should also be considered serious: events that are not immediately life threatening or which do not result in death or hospitalization but may jeopardize the subject or may require medical or surgical intervention to prevent one of the above characteristics/consequences mentioned above. Examples: allergic bronchospasm requiring intensive treatment at an emergency room or at home, blood dyscrasias, convulsions.

11.1.4. Unexpected Serious Adverse Reaction or Suspected Unexpected Serious Adverse Reaction (SUSAR)

Any adverse reaction, the nature, frequency, outcome or severity of which is not consistent with the applicable Reference Safety Information (RSI): Summary of the Product Characteristics (SmPC) of Daclatasvir,and Sofosbuvir.

11.1.5. Adverse event of special interest (AESI)

AESI will be considered as SAEs and therefore declared as such to the promoter.

For this research, AESI include:

- Any grade 4 biological or clinical event;
- Any event leading to permanent or temporary discontinuation of the investigational medicinal product;
- Any hepatitis B virus (HBV) reactivation
- Cardiac arrhythmias

11.1.6. New fact

A new fact is defined as any safety data that could modify significantly the evaluation of the benefit/risk ratio of the investigational medicinal product or the clinical trial, likely to affect the safety of participants or that could modify the investigational medicinal product administration,

the trial documentation or the conduct of the trial, or to suspend or interrupt or modify the protocol or similar trials.

Examples: a serious adverse event which could be associated with the trial procedures and which could modify the conduct of the trial, recommendations of the DSMB, if any, where relevant for the safety of subjects.

11.2. Responsibilities of the investigator

The investigator should:

- Report any non-serious AEs in the proper CRF AE form.
- Report any SAEs and AESI as a detailed, written report, using "SAE initial notification form" (in CRF SAE section), and notified to the sponsor, immediately and no later than 24h after being made aware of it.
- Ensures that all relevant information is forwarded to the sponsor within 7 days after the initial notification.
- Send to the sponsor all relevant documentation related to reported SAE (e.g. hospitalization report, laboratories results…), without omitting to make it anonymous and note the identification number of the participant in the trial.
- Follow any SAE until participant's clinical recovery is complete and laboratory results have returned to normal or baseline, or until the event has stabilized (even if the participant left the trial, when the SAE is possibly related to the study drug).
- Report all new relevant information on SAE or AESI using "SAE complementary notification form" (in CRF SAE section) and notified to the sponsor.
- Report any pregnancy and its outcome, concerning the enrolled woman, as a detailed, written report, using the "Initial pregnancy notification form" and notified to the sponsor, immediately and no later than 48 hours after being made aware of it.
- Follow the subject until the end of the pregnancy or its interruption and to notify the outcome to the sponsor using the "Final pregnancy notification form" (CRF).

Warning:

If the pregnancy outcome fulfilled a seriousness criteria (eg: **anomaly or birth defect, fetal death, voluntary or therapeutic interruption of pregnancy, miscarriage needed a hospitalization**), the investigator has to notify it to the sponsor as an SAE.

Any AE must be reported, if it occurs for a participant, throughout the duration of the research, therefore from the moment the subject signed the informed consent and until the end of the follow-up of the participant.

SAE occurring to a subject after the treatment of that subject has ended should be reported to the sponsor if the investigator becomes aware of them, specially if related to the study drug. The investigator does not need to actively monitor subjects for adverse events once the trial has ended, unless provided otherwise in the protocol.

Notification:

The investigator sends the SAEs, pregnancies recorded in corresponding form of the CRF, dated and signed, with all relevant anonymized documentation related to the SAE (e.g. hospitalization report, laboratories results…), to the Clinical Project Manager of the CTU, by email: nsothy@uhs.edu.kh

Then the CTU will send, immediately the eCRF SAE notification form and all documentation related to the SAE, to the Inserm-ANRS Pharmacovigilance unit, by fax: 01 53 94 60 02 or by email: pharmacovigilance@anrs.fr

11.2.1. AE evaluation

The adverse event

The investigator should assess, if possible, the diagnosis of all adverse events. Diagnosis, or if not available, syndrome should be reported whenever possible.

Date of "event onset" on an adverse event should be earlier (or the same day) than date of seriousness.

When medical or surgical procedures (e.g.: surgery, endoscopy, tooth extraction, transfusion) occurred; the condition that leads to the procedure should be notified.

The severity

The severity (i.e. intensity) of all AE should be graded using the appropriate table below and reported by the investigator in the corresponding form of the CRF.

" ANRS scale to grade the severity of AE in adults" (see appendix)

The seriousness

For any AE, the investigator must determine whether the event meets one or more of the severity criteria previously described.

Deaths must be reported for subjects as the outcome of an adverse event and not as an adverse event itself if the cause is known. If the cause is unknown, the death should be reported as "unknown cause of death".

The causality

The investigator must assess the causality of all AE in relation to the study drug, concomitant medication and the research, sing the definitions below.

• Reasonably possible related: there are facts (evidence) or arguments to suggest a possible or potentially possible causal related.

• Not reasonably possible related: the event seems to be more related to other causes than to the study drug, such as the patient's clinical condition (underlying pathology, medical history, etc.) or a concomitant drug (without pharmacological interaction with the drug tested).

11.2.2. Potential risks of the research and management guidelines in case of adverse event or pregnancy occurrence

Risk related to capillary and blood sample

Capillary sampling from a finger, is a medical procedure, whom may be performed on patients of any age, for specific tests that require small quantities of blood, and for patient's diagnosis, management and treatment.

Main risk for blood sample with is :

- Infection (abscess)
- Pain
- Hematoma on site injection

However the tests are carried out by professionals and precautions are used to prevent these risks.

Risk related to HVC treatment and stopping rules

For risk management of suspected adverse event, please refer to each Interventional Medicinal Product SmPC.

For combination daclatasvir/sofosbuvir:

- Patient treated with combination daclatasvir based regimen should be under close surveillance by their investigators for signs and symptoms of hypersensitivity. Hypersensitivity reactions are mostly characterized by rash, constitutional findings, and sometimes, organ dysfunction, including hepatic failure, pulmonary and renal dysfunctions. Patient should contact his/her investigator promptly if he/she develops a rash. The investigator will determine if the symptoms should be considered as potential hypersensitivity. In case of rash grade 2 or 3, dual daclatasvir based regimen could be temporally or definitely discontinued by the investigator. Discontinue daclatasvir based regimen and other suspect agents immediately if signs or symptoms of severe skin reactions or hypersensitivity reactions develop (including, but not limited to, severe rash

or rash accompanied by fever, general malaise, fatigue, muscle or joint aches, blisters, oral lesions, conjunctivitis, facial oedema, hepatitis, eosinophilia, angioedema).

- Clinical status including liver aminotransferases should be monitored. In case of elevated aminotransferase, the investigator should report to the Decision Support Committee which will provide recommendations about further investigations and potential treatment discontinuation. The decision to discontinue temporally study drugs should be documented in the CRF. All definitive study drugs discontinuation will be declared to the clinical study manager of the CTU. After study drugs been discontinued, the patient will remain in the protocol and will be followed according the calendar scheduled by the protocol if he accepts.
- Concomitant use of certain molecules with sofosbuvir and daclatasvir may require patient close monitoring (clinical or biological) when initiating or during the treatment. This is notably the case of amiodarone, beta-blockers, diabetic medication and antagonist vitamin K. Refer to the respective SmPC of sofosbuvir and daclatasvir.

11.3. Responsibilities of the sponsor

11.3.1. Recording and assessment of SAE

The sponsor

- Keep detailed records of all SAE which are reported to him by investigators.
- Assesses the causality of the SAE in relation to the study drug, concomitant medication (in case of drug-drug interaction) and the research.

In the absence of information on causality from the reporting investigator, the sponsor should consult the reporting investigator and encourage him to express an opinion on this aspect.

The causality assessment given by the investigator should not be downgraded by the sponsor. If the sponsor disagrees with the investigator's causality assessment, the opinion of both the investigator and the sponsor should be provided in the report to the National Competent Authority.

All SAE for which the investigator or the sponsor considers that a causal relationship is a reasonable possibility are considered as suspected SAR.

- Assesses if the SAE is expected or not using the applicable Reference Safety Information.

11.3.2. Reporting of safety data to the National Competent Authority and the Ethics Committee

11.3.2.1. SUSAR reporting (Suspected Unexpected Serious Adverse Reaction)

All Suspected Unexpected Adverse Reactions (SUSAR) have to be reported, within the legal timeframe, by the sponsor to Ethic Committee and DSMB.

The timelines for expedited initial reporting (day 0) starts as soon as the information containing the minimum reporting criteria has been received by the sponsor.

For fatal and life-threatening SUSAR, the sponsor should report within 7 calendar days.

SUSAR which are not fatal and not life-threatening are to be reported within 15 calendar days.

If significant new information on an already reported case is received by the sponsor, this information should be reported as a follow-up report within 8 days after being made aware of the relevant complementary information.

11.3.2.2. New fact reporting

When a new event is likely to affect the safety of participants, the sponsor and the investigator take appropriate urgent safety measures to protect participants against any immediate hazard.

The sponsor inform without delay the Ethic Committee and DSMB of safety data that may be relevant in terms of subject safety, or safety issues which might alter the current benefit-risk assessment of the trial.

The safety office shall transmit a written report, within 15 days to the applicable Competent Authority and DSMB.

If the new fact requires substantial amendments, the sponsor should notify any substantial protocol modification, to the National Competent Authority and the concerned Ethic Committee, within 15 days of the safety measures implementation.

11.3.2.3. Annual safety reporting

Once a year throughout the clinical trial, the sponsor should submit to the DSMB (at demand), principal investigator, project leader, international project leader and the Ethics Committee, an annual safety report.

The annual safety report may be submitted to the coordinating investigator for approval.

The annual safety report should be submitted no later than 60 calendar days from the first patient inclusion to conduct the clinical trial in any country.

Appendix or Figure x : AE Notification

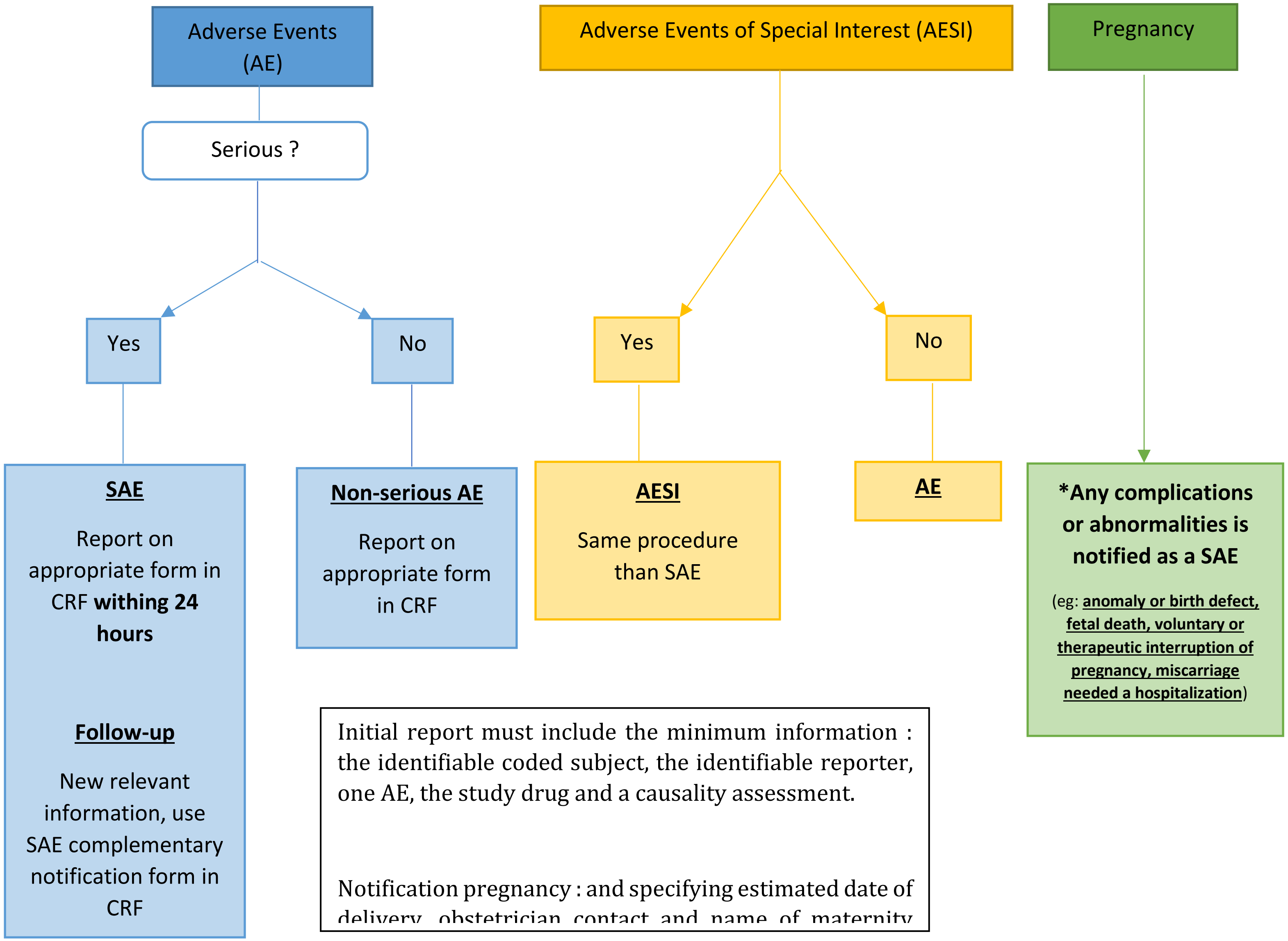

12. TRIAL BOARDS AND COMMITTEES

12.1 Trial scientific committee

Composition

The trial scientific committee will consist of: (i) the investigators, the clinical coordinators and statisticians/methodologists of the trial; (ii) the virologists; (iii) a representative of the communicable disease department of the Ministry of Health; (iv) other external experts; and (v) sponsor representatives. Members of the scientific committee are listed in annex 1.

Meeting agenda

The trial scientific committee will meet before the beginning of the inclusion phase, as well as once or twice per year until the end of the trial. The trial sponsor or one or several board members may also request a special meeting at any time.

Role

The role of the trial scientific committee is to ensure that the trial is carried out appropriately, not only scientifically but also clinically and ethically. The members of the scientific committee report directly to the sponsor.

- They will ensure that the trial personnel carry out the trial properly, adhere to the trial protocol and maintain patient safety;
- They will guarantee that the trial remains scientifically relevant by ensuring the relevancy of the trial questions and that the methods used are valid and appropriate;
- They will make all decisions regarding necessary and relevant protocol modifications, such as:
    - Actions needed to facilitate patient recruitment;
    - Decisions to open or close participating trial sites.
- They will enforce the rules pertaining to access to the trial data as well as reports and publications of the results;
- They will remain in contact with the sponsor, the DSMB and the coordinating investigators, and will make sure investigators and other trial personnel have access to up-to-date information.

At the end of each meeting, a report containing the meeting minutes, signed by the Scientific Committee chair will be sent to the members of the scientific committee, DSMB Members and the director of ANRS EID.

12.2 Data Safety & Monitoring Board (DSMB)

Composition

The DSMB members will be selected in collaboration between the coordinating investigators and the ANRS EID before the beginning of the inclusion phase.

Meeting agenda

The DSMB will meet once at the beginning of the inclusion phase, and at least every 12 months until the end of the trial. The sponsor, the scientific committee or the DSMB may request to increase the frequency of these meetings.

Role

The DSMB is a consultative board for the scientific committee and to the sponsor. Its members will provide general advice on the progress of the trial, including the rate of inclusions, the quality of follow-up, the overall rate of drug-related adverse events, changes in biological markers, the overall incidence of primary outcomes, and the number of subjects needed.

They will help to make difficult decisions that require an independent assessment while the trial is underway. Advice may be needed regarding:

- Premature discontinuation of the trial (because the rate of adverse events is high, the trial is no longer feasible, or the available data are sufficient);
- Substantial changes to the protocol that becomes necessary during the inclusion or follow-up phases, or to account for new scientific information.

The DSMB will conduct and interpret intermediate analyses, if any.

At the end of each meeting, a written report containing the DSMB's opinion will be sent to the sponsor and the members of the scientific committee.

12.3 Decision Support Committee (DSC)

Roles of the DSC

The DSC will provide advices and recommendations to investigators regarding:

- Occurrence of liver adverse event
- Any morbidity event occurring during the study
- Decision on DAA management
- Any important clinical issue for which the investigators wish to share experience before taking a decision

Composition of the DSC

The DSC will consist of 1/ Two physicians specialized in HCV infection 2/ One virologist 3/ The two principal investigators of the study. Other specialists could be called at DSC's request.

Organization of the DSC

The DSC will meet regularly and must be accessible on rapid request through phone calls, e-mails, private online forum, or any other means in case of morbidity event.

The investigators of each site will be responsible to prepare the documents for the meeting and to send it by e-mail at least two days before the meeting.

The responsibility of DSC is to provide a recommendation on a written document. One member of the DSC will be in charge of the written report. This report will be send to the methodological centre in UHS.

13. COORDINATION, MONITORING, DATA MANAGEMENT

Coordination

The UHS Research Grant Management Office is the referent for the trial methodology and management. The UHS team is responsible, in coordination with the clinical, laboratory and administrative coordinators, for the overall trial management (preparation and organisation of the trial, monitoring, data management and analysis). The team will include the clinical coordinators, the statistician, the clinical research assistants, one Lab technician and one data manager.

- **Preparation and organisation of the trial**: the UHS team will finalize and review the Case Report Forms (CRF) with all the collaborators, prepare the recruitment procedures, and organize the trial reference documentation. The MDMC team and the clinical coordination team will prepare the operation manual describing in details the trial procedures for each collaborators (clinical and laboratory activities, treatment dispensation, data management).
- **Database**: The trial database will be developed and maintained by the MDMC in collaboration with NCHADS Data Management Unit. The data manager will also be in charge of the data entry and implementation of data quality insurance plans. H/She has to prepare daily monitoring reports and the data file necessary for the trial analysis.
- **Data analysis and analysis report**: will be coordinated at the UHS MDMC.
- **Reporting**: the operational team will be responsible for finalizing:
    - the necessary reports and presentations for the Scientific Advisory Board and the DSMB meetings;
    - the trial yearly progress reports; The operational team will bring its support in preparing any communications on the trial (poster, oral presentation, papers).

Trial documents

Essential trial documents will be retained at the MDMC for 15 years. Data will be kept on a secured database installed on a server at the MDMC centre.

Data management

1.1.1 Data collection

Screening phase

E-CRF in Khmer language with the patient code of participants for both arms (Community and facility arms) will be used to collect data of the participant in this study. For that purpose, tablets will be given to community workers and nurses. Each community worker for community arm and each health centre for facility arm will get his own tablet. Data will be collected in household for community arm and in health centre for facility arm. At the end of each day, for community arm, community workers will be in charge to return the tablets in referral health centre. Tablets will be stored in a secured cupboard.

Treatment phase

CRF paper in English/Khmer language with the patient code of participants will be used to collect data of the participants. Data will be collected during each consultation in the Provincial Hospital.

The CRA from the MDMC will come on a regular basis to check for completeness, accuracy and legibility of data reported on the CRF. He/she will bring back the validated CRF to the MDMC for data entry.

1.1.2 Data entry and checking

Data will be entered at the MDMC (UHS). The trial database will be developed by a database developer; he will closely work with the coordination team.

After entry, the database will be checked for consistency. If any inconsistency or question on the data, the CRA will go back to the clinical monitors at sites for clarification during their monitoring visit. The corrections will be entered and followed in the data base.

Data protection and confidentiality

All the collected patient data will be strictly confidential and coded.

Only persons mandated by the sponsor and involved in the study management and health authorities are able to access to medical files of patients in order to check the accuracy of the collected data.

In order to respect patient confidentiality, each subject who accepts to participate in the study will be allocated a unique identifying code, which is composed of arm, village code, household code and participant code: |__| / |__|__| / |__|__|__| / |__|__||__| (Arm / Village / HH / participant).

Only the code of the subject will be reported in the Case Report Form (CRF).

The trial data will be recorded and be managed informatically at the Monitoring and data management center (MDMC) of UHS and the ANRS pharmacovigilance unit. Inserm-ANRS declares to the French National Commission of Informatic and Liberty (CNIL) that data management is compliant with the requirements of the modified Law n° 78-17 of January 6 1978 – Law 2018-493 - of June 20 2018 relative to information technology, files and civil liberties.

Monitoring

Monitoring will be conducted according to the Good Clinical Practice (ICH Harmonized Tripartite Guidelines for Good Clinical Practice 1996) to guarantee the good quality of the research and safeguard the health and the rights of the patient.

1.1.3 On site

The on-site trial activities (patient management; drug management and dispensation; trial monitoring) will be done under the responsibility of the head of project, based at the UHS in Phnom Penh. They will be responsible for coordinating all clinical activities for the trial:

- Recruitment, training and management of the clinical monitors;
- Organising and supporting clinical patient follow-up on site;
- Supervision of trial drug management;

- Member of the decision support committee;

On each investigational site, a physician known will have the medical responsibility to conduct the trial according to the protocol. They will be responsible, under the supervision of the head of project, in organizing the trial follow-up and management on site:

- Organisation of patient screening, enrollment and follow-up;
- Report clinical and biological data in the Case Report Form (CRF) according to the most updated SOP that will be provided;
- Report and document all Serious Adverse Events and sent follow-up report when necessary;
- Communication and coordination with the Decision Support Committee (DSC) and MDMC;
- Preparation and facilitation of the monitoring visit;

The clinical monitor must give the MDMC monitor access to relevant hospital or clinical records, to confirm their consistency with the CRF entries. No information about the identity of the subjects should appear on the CRF. The CRF must be completed within the week following the patient's visit. All CRF must be signed by the physician responsible of the patient follow-up. CRF will be considered as the source document for all the study sites regarding all the informations it contains.

1.1.4 Monitoring activities

Monitoring activities will be coordinated by the coordination center. The trial monitors will visit the site regularly during the study:

- Check the adherence to the protocol and to Good Clinical Practice (patient informed consent, protocol visit and blood test schedule…);
- Assist in the trial organisation and management on site (communication between collaborators, treatment or material availability, tracing laboratory samples follow-up and result in collaboration with the lab technician);
- Check the completeness and the accuracy of patient data on the CRF;
- Collect and check the Serious Adverse Events reporting, documentation and follow-up, and send the forms to the sponsor;
- Evaluate the progress of enrolment.

14. STATISTICAL ANALYSES

Calculation of number of patients needed

The study will be powered to detect an increase of HCV combined-testing uptake from 60%in the facility arm to 80% in the community arm among subjects older than 40 years.

According to Cambodia DHS report 2014, the average rural household size is 4 persons and the proportion of persons aged more than 40 years old is 25%, the cluster size is so estimated to 50.

The following assumptions and definitions will be used for the sample size calculation:

- an intra-class correlation of 0.01
- significance level α= 0.05
- power (1-β) = 80%
- clusters size: 50
- Design effect: 1.5

When accounting for the above-mentioned assumptions, 300 patients will need to be screen for HCV RNA and so, 6000 individuals aged above 40 years old will need to be screened by RDT (5% of patients are expected to have a positive HCV Ab). With an estimated median testing uptake of 60-70%, if we want to reach 6000 participants screened, it would be necessary to propose testing to 8000 participants from 160 clusters (80 clusters per intervention).

Statistical analysis plan

Description of the participants' characteristics at inclusion (sociodemographic characteristics). These variables will be described by their median, interquartile range, mean, standard deviation, or frequencies (%), as appropriate.

Primary outcome

The percentage, and its 95% confidence interval, of persons tested for HCV RDT AND HCV RNA and aware of their status among the total number of persons eligible residing in the region where the intervention takes place will be compared between the 2 arms.

An analysis stratified on age and province of location will also be performed.

Secondary outcomes

The percentage, and its 95% confidence interval, of persons tested for HCV RDT and aware of their status among the total number of persons eligible residing in the region where the intervention takes place will be compared between the 2 arms.

An analysis stratified on age and province of location will also be performed.

The number of persons with HCV active infection (positive HCV Ab and positive HCV RNA) and results given and explained among the total number of persons eligible residing in the region where the intervention takes place will be compared between the 2 arms

The number of persons with at least one consultation in the Provincial Hospital among the estimated total number of persons with active infection residing in the region where the intervention takes place will be compared between the 2 arms.

Analysis of variance (ANOVA) will be used to detect the significance differences between the primary and secondary outcome variables among the two clustered trial arms. Confidence levels, confidence intervals, and effect sizes will be computed. ANCOVA model will be conducted to analyse the main outcome intervention by taking baseline as a covariate, and other descriptive correlation statistics will be tested for the association between covariate.

15. ACCEPTIBILTY STUDY (new)

15.1. Summary

To address the burden of Hepatitis C virus (HCV) which is more prevalence and at higher risk of advanced liver disease among population older than 40 years in Cambodia, interventions focused on the cascade of care seem to be the most appropriate measure. Community versus facility-based services to improve the screening of active HCV infection in Cambodia (CAM-C) is conducting in 2 provinces in Cambodia (Siem Reap and Kampong Cham provinces) and the aims are to compare the effectiveness of a community-based intervention to a facility-based intervention to improve the combined-testing uptake (Ab & RNA) of HCV infection among general population aged more than 40 years old in Cambodia. The CAM-C methodology is described in the attached.

CAM-C did not yet conclude whether there is enough evidence to support the hypothesis that community-based services are more accepted by the beneficiaries. Whatever the result is, in order to scale up the most acceptable strategy countrywide, it is necessary to understand the cost-effectiveness and acceptability of each strategy.

Our proposed qualitative study will explore the acceptability of facility-based and community-based HCV testing/screening strategies in HCV management and control.

The overall objective of this qualitative study is to explore the acceptability of HCV testing among the population toward the facility-based testing intervention and community-based testing intervention.

This is a qualitative assessment method where participants will take part in semi-structured interviews and focus groups discussion.

The semi-structured interviews will be conducted with leaders in health sectors such as head of HCs, head of ODs; and health care providers who directly involved in the intervention such as HC staff, community health workers, and VHSG. The interviews will explore the context of community-based as well as facility-based HCV testing; the health providers' interests, expectations, and concerns; the perceived strengths and weaknesses of the two testing intervention methods; and the degree to which the HCV testing respond to Cambodia's HCV management and control needs.

Focus groups discussion will be conducted with people who accepted to be tested for HCV and also with people who do not accept to be tested for HCV in the CAM-C study. To

increase the diversity of data, the focus groups will be conducted with a purposively selected sample of beneficiaries in the 8 geographical areas of the CAM_C study.

While the exact number of participants cannot be precisely determined, based on the research question and the previous studies on similar topics, we expect to get approximately:

- 10 interviews with stakeholders (Head of health centres and head of ODs);
- 20 interviews with healthcare providers who are directly involved in the intervention such as HC staff, community health workers, and VHSG;
- 8 focus group discussions x 8 participants/focus group = 64 participants; 4 focus group discussions are to conduct among people who accepted to be tested for HCV and 4 focus group discussions are to conduct among people who did not accept to be tested for HCV.

We will conduct simple tabulations using Stata 17 (Stata Corporation, Texas, USA) to summarize the characteristic of participants (doctors, nurses, midwives, and other beneficiaries). The qualitative analysis will be done using grounded theory principles.

The study potentially provides policymakers with evidence that may be used to inform decisions regarding HCV testing/screening cascade in order to contribute to the debates surrounding HCV care and control.

15.2. Research questions

RQ1: What are the benefits, drawbacks, and challenges of facility-based HCV testing strategy?
RQ2: How feasible of facility-based HCV testing strategy in implementing countrywide?
RQ3: What are the benefits, drawbacks, and challenges of community -based HCV testing strategy?
RQ4: How feasible of community-based HCV testing strategy in implementing countrywide?

15.3. Study objectives

The overall objective of this qualitative study is to explore the acceptability of HCV testing among the population toward the facility-based testing intervention and community-based testing intervention.

The specific objectives are:

- To understand the advantages of facility-based HCV testing
- To understand the advantages of community-based HCV testing

- To understand the disadvantages of facility-based HCV testing
- To understand the disadvantages of community-based HCV testing
- To understand the challenges of facility-based HCV testing
- To understand the challenges of community-based HCV testing
- To explore ways to improve HCV testing uptake in Cambodia to better address the HCV burden in the country

15.4. Methods

This is a qualitative assessment where participants will take part in semi-structured interviews and focus groups discussions

Study setting

The semi-structured interviews will be conducted with leaders in health sectors such as heads of HCs, heads of ODs; and healthcare providers who are directly involved in the intervention such as HC staff, community health workers, and VHSG. The interviews will explore the context of community-based as well as facility-based HCV testing; the health providers' interests, expectations, and concerns; the perceived strengths and weaknesses of the two testing intervention methods; and the degree to which the HCV testing responds to Cambodia's HCV management and control needs. The individual interviews will be conducted by experienced researchers based on an interview guide that would contain both common items and items adapted to the respondents' background, expertise, and experience. Each interview is expected to be about 60-90 minutes long.

Focus groups discussion will be conducted with people who accepted to be tested for HCV and also with people who do not accept to be tested for HCV. To increase the diversity of data, the focus groups will be conducted with a purposively selected sample of beneficiaries in each geographical area: 4 geographical areas in Kampong Cham province and 4 geographical areas in Siem Reap province. In each location, one focus group discussion will be held. In each province, we will conduct 2 focus group discussions among the population who accepted to be tested for HCV, and another 2 focus group discussions among population who do not accept to be tested for HCV in the CAM-C study. Each focus group will include eight participants. The participants must be similar in terms of social class and diverse in terms of gender. A focus group guide will be developed to facilitate the discussion. The guide will address participants' knowledge of and experience with using and not using the HCV testing services; the decision-making process in seeking the test; the barriers to the decision; the formal and informal costs of accessing health services; the social construction of HCV, including health beliefs and practices;

the experiences of navigating the healthcare system; and the perception of areas and how the HCV testing should be implemented. The focus group discussions are expected to last 90-120 minutes each.

The debriefing will take about 10 minutes for interview participants and 15 minutes for focus group participants. The interviews and focus group discussions will be audio-recorded (with participants' consent), transcribed, and translated into English.

Study materials

Appendix 7: includes the interview guides for the interviews with head of HCs, head of ODs, and other health care providers, and focus group discussion guides for people in the coverage area of the CAM-C study who accepted to be tested for HCV and who do not accept to be tested for HCV.

The research materials will be translated into Khmer language, and the research team will provide training to the research assistants on qualitative data collection, transcribing the interviews and focus groups discussion, and translating them into English language.

Study population

This is a qualitative study. According to the principles of qualitative methodological framework, the sampling is guided by the analysis (theoretical sampling), and the sample size is not predetermined. Instead, enough participants will be recruited to reach data saturation (i.e., new interviews/focus groups would not yield new meaningful data) and theoretical saturation (i.e., the categories, subcategories, properties, and the relation among them are fully developed and more information will be redundant).

While the exact number of participants cannot be precisely determined, based on the research question and the previous studies on similar topics, we expect to get approximately:

- 10 interviews with stakeholders (Head of health centers and head of OD);
- 20 interviews with health care providers who directly involved in the intervention such as HC staff, community health workers, and VHSG;
- 8 focus group discussions x 8 participants/focus group = 64 participants; 4 focus group discussions are to conduct among people who accepted to be tested for
- HCV and 4 focus group discussions are to conduct among people who did not accept to be tested for HCV.

The Head of HCs, head of ODs and health care providers will be identified by UHS research teams before implementing the research. To maximize the diversity of the sample, we

will identify a variety of professionals, including health officials, doctors, nurses, midwives and community health workers. Interviewing them will provide a rich and nuanced understanding of how the HCV testing procedure in Cambodia respond to the country's needs regarding HCV management and control.

Identified interviewees will be reached through personal contact or e-mails. The scripts for personal contact and introductory letters/e-mails have been included in **Appendix 8**. They provide a brief explanation of the nature and purpose of the study, potential risks and benefits, compensation for participation, estimated duration, and contact information. If they are interested in taking part in the study, they will undergo the informed consent process before starting the interview/focus group discussion.

No screening will be conducted. The study has broad inclusion criteria, allowing for the participation of a wide variety of participants. For the head of HC/OD and health care providers, the selection of potential participants will be predetermined based on relevance to the research objectives. To maximize the diversity of experiences and perspectives and to get a comprehensive understanding of the topic of interest, the potential participants occupy different positions in the health system (e.g., health officials, doctors, nurses, midwives, community health workers, etc.) and possess different types of expertise.

We will conduct simple tabulations using Stata 17 (Stata Corporation, Texas, USA) to summarize the characteristic of participants (doctors, nurses, or midwives). The qualitative analysis will be done using grounded theory principles.

Strategies to mitigate the risks

Although the recruitment of participants does not collect identifiable information, it is possible that some potential participants are contacted through phone calls or emails. After the data collection is completed, we do not need to contact the participants, the identifiable information (names and email addresses) will be deleted from our records within 15 days. The identifiable information will not be associated with research data.

Other than that, we could anticipate some other potential risks.First, some participants could become distressed during the research process, for instance when recalling the state of illness and the hardship seeking medical care. However, the topics are not sensitive and do not emphasize the disclosure of emotions and feelings. Participants could feel the risk of deductive disclosure. Anonymize the data in our reports and publications (replacing any identifiable information, such as names, locations, or job titles) with pseudonyms and general descriptors will help to minimize this risk. If some information or quotes pose a high risk of identifying the

participants, we will either exclude such information or modify them to reduce the risk of identification while preserving the essence of the data. In case this alteration takes place, it will clearly be indicated in the methodological section of the report.

Second, there might be the risk of potential upset. If it happens, we will employ several strategies to mitigate the risk. First, during the informed consent process, we will clearly communicate the research objectives and the types of questions to be asked. Potential participants will be informed that they may encounter sensitive topics and can choose not to participate if they feel uncomfortable. During the informed consent process, potential participants will also be told that the participation is entirely voluntary, and they have the right to withdraw at any moment from the study or skip questions with which they do not feel comfortable, without any repercussions. During the interview or focus group, the researcher will look for signs of distress in participants, and will handle them with care: checking with the participant to see if they feel comfortable with continuing the interview/focus group, taking a break, and reiterating the right of withdrawal. After the interview or focus group discussion, participants will be debriefed and given the chance to express their feelings or discuss their concerns.

Third, there could be also minimal risks of physical harm or discomfort, for instance the prolonged sitting, and eye strain. To mitigate the risk of discomfort due to sitting for extended periods during interviews or focus groups, we will provide comfortable sitting arrangements and ask participants if they would like to have breaks. To mitigate the potential risk of eye strain while reading the informed consent materials, we will use clear font types and sizes.

There are no participants who should be excluded because of the potential risks. However, minors, individual with severe cognitive impairments, and individuals in the acute stage of a mental health crisis will be excluded as they may not be appropriate asking them for consent.

The data will be stored offline, under a password-protected folder at UHS research office, by the study investigators and an external disk drive for backing up. The data will be accessed or viewed by the PIs and by the members of the research team only.

15.5. Benefits

There are no direct benefits to participants but the participant will receive a gift in maximum 15$ as compensation for their time spend. However, the research has the potential of providing indirect benefits to participants. By sharing ideas, experiences, and concerns about

the two HCV testing strategies, participants get the chance to have their voices heard and contribute to the debates surrounding HCV care and control. The study also potentially provides policymakers with evidence that may be used to inform decisions regarding HCV testing/screening cascade.

16. SCIENTIFIC COMMUNICATION

All written or oral communications of the study's results must receive the approval of the coordinating investigators and of the scientific committee of the study.

The MDMC will realize data analysis. This analysis will lead to a report, submitted to the scientific committee for approval. This report will help to prepare scientific publications whose final draft must be approved by the scientific committee (SC).

SC shall have sole competence to include – if necessary – additional researchers to the papers related to the research project. All rules related to submission/publication, defined by ANRS EID, will be followed.

All publications will include the name of sponsor as follows ("Inserm-ANRS. French National Institute for Health and Medical Research ANRS Emerging Infectious Diseases (ANRS EID) – Autonomous agency of Inserm is the sponsor of the project") followed by the ANRS EID study number (ANRS 12384 +Cam-C), as well as the investigators, the composition of SC

In case of ancillary studies, results will be published after approval by SC. In addition, these results will be published only after the publication of the main results obtained during the research project. The main project will be quoted (ANRS 12384). Results obtained through ancillary studies will be also transmitted to Inserm-ANRS for information.

17. ETHICS AND LEGAL CONSIDERATIONS

17.1. Ethics

This research project will be conducted with regard to fundamental ethical principles that are described in the updated version of the Declaration of Helsinki (64th World Medical Association [WMA] General Assembly, Fortaleza, Brazil, October 2013), and in the ANRS ethics charter for research in developing countries (July 2017) (https://www.anrs.fr/fr/actualites/377/charte-dethique

17.2. Legal and regulatory considerations

This trial is being conducted in conformity with the Public Health Code, as modified, notably, by Public Health Law no. 2004-806 of August 9, 2004 and its subsequent texts, and the Law n° 2012-300 of March 05th 2012 on research involving the human person (Jardé Law) and its subsequent texts.

The protocol complies with the Decision of November 24, 2006, part of the Public Health Code, established the guidelines for Good Clinical Practice for biomedical research on drugs for human use and ICH Good Clinical Practice E6 (R2) 09 November 2016 as well as European Directive no. 2005/28/EC establishing the principles and detailed guidelines concerning the application of good clinical practice with regard to investigational drugs for human use, as well as the requirements for granting authorization to manufacture or import these drugs.

The protocol, information sheets, and informed consents (**appendix 1 and 2**) will receive the approval from the National Ethics Committee for Health Research (NECHR). Subsequent amendments after the start of the test will also be submitted for approval to the NECHR.

The research will be performed according to the present protocol. All researchers and investigators participating to this project will respect the protocol, especially in obtaining informed consent (see below).

Amendment to the protocol

Any "substantial" change is subject to a written amendments which is submitted to the Scientific Research Council, the Sponsor, and then for approval to the NECHR. Substantial changes are those that have a significant impact on the following criteria:

- the protection and safety of people,

- the conditions of validity of the search,

- the interpretation of scientific documents that support the research process (particularly the protocol),
- how to conduct research, such as changing the duration of follow-up or consultation rhythms, removals or other invasive medical procedures.

After approval by the NECHR and the sponsor, the amendment is signed by the coordinating investigators and the sponsor, in the same way as the version of the protocol approved by the competent authorities.

All amendments to the protocol should be made known to all investigators participating in the research. The investigators undertake to respect the content.

17.3. Information and consent

The consent of each potential participant is a prerequisite before starting any sampling or before obtaining any specific information related to the research project. The informed consent must be signed by each participant after giving full research description:

- What is being studied?
- What is the procedure/protocol?
- Who is sponsoring the study?
- What are the risks and burdens?
- What are the benefits?
- Whom to contact with questions/concerns

Each potential participant will be given time to think about the information before making a decision.

Each subject must fully understand that they have the full freedom to accept or refuse to participate in the study. Once one subject is included in the research project, he has to understand that he can also withdraw from it whenever he wants without any problems/consequences and he will continue to benefit from the regular medical care and check-up.

When one person gives his/her consent for participating to the study, he (she) will write his (her) last name and first name, date and sign the informed consent.

Each signed informed consent will be kept temporarily in a secure cupboard in health centers and centralized in the research grant management office of UHS in a safe manner and in a safe place for a total duration of 15 years after the end of the research project.

17.4. Data confidentiality

All information collected from enrolled subjects will be strictly confidential and coded.

During the implementation and by the end of the research, information collected among participants must be de-identified and names, personal addresses must not be indicated.

For this purpose, each participant will receive a coded identification (ID) number.

17.5. Type of collected data during the research project

All data obtained through this research project will be recorded in a database at MMC (UHS). All the computer data will be treated in accordance with the Data Protection Act of 6th January 1978 (law n°78-17 Data Protection [DP] Officer) that was modified on 6th August 2004 (law 2004-801 DP Officer), with the implementing decree numbered 2005-1309 on 20th October 2005. We will follow this procedure in order to be in agreement with the treatment of personal data obtained through medical research programs.

17.6. Final report

MMC will write the final report, in partnership with principal investigators.

This final report will be established within one year following the end of the research and will be transmitted to Insem-ANRS and NECHR.

17.7. Archiving system

Forms and data related to the research project are key documents. They can be useful to demonstrate that researchers and investigators respect GCP and current laws/legislation.

Consequently, all these documents will be archived by MMC and Inserm-ANRS in accordance with current archiving regulations for clinical research

Informed consents will be kept in a safe manner at MMC in sealed envelopes on which ID numbers of the participants will be indicated, as well as name and signature of the coordinating investigators.

No documents must be destroyed without prior authorization from Inserm-ANRS.

18. ACCESS TO DATA AND SPECIMENS

All research data and all collected samples will be under the responsibility of the MMC and coordinating investigators.

In case of request by other research teams to use results obtained during the research and/or to use stored samples, these teams will write a proposal and send it to the principal investigators, the scientific committee and the NECHR for approval.

After the dissolution of the SC, data and samples will be under the responsibility of Inserm-ANRS and the coordinating investigators. In case of request by other research teams to use results obtained during the research and/or to use stored samples, these teams will write a proposal and send it to Inserm-ANRS, which will take a decision in agreement with coordinating investigators and NECHR.

Data or samples may be transferred to national or French or health authorities (drug agency ...), to other national or international private or public research teams. The data or samples thus transmitted will be framed by appropriate and adapted guarantees provided in a sharing agreement under the control of Inserm-ANRS and under conditions guaranteeing the confidentiality of your data or samples.

19. DUTIES OF INVESTIGATORS

According to GCP aimed to achieve high quality of the research, each investigator must:

- To respect participants' rights and to ensure subjects' welfare,
- To ensure his availability as well as availability of his team,
- To ensure that enrollment will be feasible according to the research protocol,
- To organize technical infrastructures for the implementation of sampling, filling in of questionnaires and archiving of documents/records during the research study and 15 years after the end of the research,
- To collect and archive in a safe way signed informed consents,
- To ensure that researchers are following the protocol and to allow completed questionnaires to be regularly sent to MMC,
- To accept a possible audit of the research project carried out by Inserm-ANRS itself or by other agencies if necessary.

20. REFERENCES

1. Caruana SR, Kelly HA, De Silva SL, Chea L, Nuon S, Saykao P, et al. Knowledge about hepatitis and previous exposure to hepatitis viruses in immigrants and refugees from the Mekong Region. Aust N Z J Public Health. 2005 Feb;29(1):64–8.

2. Ol HS, Bjoerkvoll B, Sothy S, Van Heng Y, Hoel H, Husebekk A, et al. Prevalence of hepatitis B and hepatitis C virus infections in potential blood donors in rural Cambodia. Southeast Asian J Trop Med Public Health. 2009 Sep;40(5):963–71.

3. Thüring EG, Joller-Jemelka HI, Sareth H, Sokhan U, Reth C, Grob P. Prevalence of markers of hepatitis viruses A, B, C and of HIV in healthy individuals and patients of a Cambodian province. Southeast Asian J Trop Med Public Health. 1993 Jun;24(2):239–49.

4. Akkarathamrongsin S, Praianantathavorn K, Hacharoen N, Theamboonlers A, Tangkijvanich P, Poovorawan Y. Seroprevalence and genotype of hepatitis C virus among immigrant workers from Cambodia and Myanmar in Thailand. Intervirology. 2011;54(1):10–6.

5. Goyet S, Lerolle N, Fournier-Nicolle I, Ken S, Nouhin J, Sowath L, et al. Risk factors for hepatitis C transmission in HIV patients, Hepacam study, ANRS 12267 Cambodia. AIDS Behav. 2014 Mar;18(3):495–504.

6. Yamada H, Fujimoto M, Svay S, Lim O, Hok S, Goto N, et al. Seroprevalence, genotypic distribution and potential risk factors of hepatitis B and C virus infections among adults in Siem Reap, Cambodia. Hepatol Res. 2015 Apr;45(4):480–7.

7. Lerolle N, Limsreng S. High Frequency of Advanced Hepatic Disease among HIV/HCV Co-Infected Patients in Cambodia: The HEPACAM Study. Journal of AIDS & Clinical Research [Internet]. 2012 [cited 2014 May 4];03(06). Available from: http://www.omicsonline.org/2155-6113/2155-6113-3-161.digital/2155-6113-3-161.html

8. Eav S, Schraub S, Dufour P, Taisant D, Ra C, Bunda P. Oncology in Cambodia. Oncology. 2012;82(5):269–74.

9. Narin P, Hamajima N, Kouy S, Hirosawa T, Eav S. Characteristics of liver cancer at Khmer-soviet Friendship Hospital in Phnom Penh, Cambodia. Asian Pac J Cancer Prev. 2015;16(1):35–9.

10. Chassagne F, Rojas Rojas T, Bertani S, Bourdy G, Eav S, Ruiz E, et al. A 13-Year Retrospective Study on Primary Liver Cancer in Cambodia: A Strikingly High Hepatitis C Occurrence among Hepatocellular Carcinoma Cases. Oncology. 2016;91(2):106–16.

11. Nasrullah M, Sergeenko D, Gvinjilia L, Gamkrelidze A, Tsertsvadze T, Butsashvili M, et al. The Role of Screening and Treatment in National Progress Toward Hepatitis C Elimination - Georgia, 2015-2016. MMWR Morb Mortal Wkly Rep. 2017 Jul 28;66(29):773–6.

12. Easterbrook PJ, WHO Guidelines Development Group. Who to test and how to test for chronic hepatitis C infection - 2016 WHO testing guidance for low- and middle-income countries. J Hepatol. 2016 Oct;65(1 Suppl):S46-66.

13. Guidelines for the Screening, Care and Treatment of Persons with Hepatitis C Infection [Internet]. Geneva: World Health Organization; 2014 [cited 2016 Mar 16]. (WHO Guidelines Approved by the Guidelines Review Committee). Available from: http://www.ncbi.nlm.nih.gov/books/NBK263483/

14. Aspinall EJ, Doyle JS, Corson S, Hellard ME, Hunt D, Goldberg D, et al. Targeted hepatitis C antibody testing interventions: a systematic review and meta-analysis. Eur J Epidemiol. 2015 Feb;30(2):115–29.

15. Zuure FR, Urbanus AT, Langendam MW, Helsper CW, van den Berg CHSB, Davidovich U, et al. Outcomes of hepatitis C screening programs targeted at risk groups hidden in the general population: a systematic review. BMC Public Health. 2014 Jan 22;14:66.

16. Brouard C, Le Strat Y, Larsen C, Jauffret-Roustide M, Lot F, Pillonel J. The undiagnosed chronically-infected HCV population in France. Implications for expanded testing recommendations in 2014. PLoS ONE. 2015;10(5):e0126920.

17. Bert F, Rindermann A, Abdelfattah MA, Stahmeyer JT, Rossol S. High prevalence of chronic hepatitis B and C virus infection in a population of a German metropolitan area: a prospective survey including 10 215 patients of an interdisciplinary emergency unit. Eur J Gastroenterol Hepatol. 2016 Jul 19;

18. Orkin C, Leach E, Flanagan S, Wallis E, Ruf M, Foster GR, et al. High prevalence of hepatitis C (HCV) in the emergency department (ED) of a London hospital: should we be screening for HCV in ED attendees? Epidemiol Infect. 2015 Oct;143(13):2837–40.

19. Calderon Y, Cowan E, Schramm C, Stern S, Brusalis C, Iscoe M, et al. HCV and HBV testing acceptability and knowledge among urban emergency department patients and pharmacy clients. Prev Med. 2014 Apr;61:29–33.

20. O'Connell S, Lillis D, Cotter A, O'Dea S, Tuite H, Fleming C, et al. Opt-Out Panel Testing for HIV, Hepatitis B and Hepatitis C in an Urban Emergency Department: A Pilot Study. PLoS ONE. 2016;11(3):e0150546.

21. Coyle C, Kwakwa H, Viner K. Integrating Routine HCV Testing in Primary Care: Lessons Learned from Five Federally Qualified Health Centers in Philadelphia, Pennsylvania, 2012-2014. Public Health Rep. 2016 Jun;131 Suppl 2:65–73.

22. Shiha G, Metwally AM, Soliman R, Elbasiony M, Mikhail NNH, Easterbrook P. An educate, test, and treat programme towards elimination of hepatitis C infection in Egypt: a community-based demonstration project. Lancet Gastroenterol Hepatol. 2018 Jul 17;

23. Gupta E, Agarwala P, Kumar G, Maiwall R, Sarin SK. Point -of -care testing (POCT) in molecular diagnostics: Performance evaluation of GeneXpert HCV RNA test in diagnosing and monitoring of HCV infection. J Clin Virol. 2017 Mar;88:46–51.

24. McHugh MP, Wu AHB, Chevaliez S, Pawlotsky JM, Hallin M, Templeton KE. Multicenter Evaluation of the Cepheid Xpert Hepatitis C Virus Viral Load Assay. J Clin Microbiol. 2017 May;55(5):1550–6.

25. Grebely J, Lamoury FMJ, Hajarizadeh B, Mowat Y, Marshall AD, Bajis S, et al. Evaluation of the Xpert HCV Viral Load point-of-care assay from venepuncture-collected and finger-stick capillary whole-blood samples: a cohort study. Lancet Gastroenterol Hepatol. 2017 Jul;2(7):514–20.

26. Nouhin J, Bollore K, Castera-Guy J, Prak S, Heng S, Kerleguer A, et al. Analytical and field evaluation of the Biocentric Generic HCV assay on open polyvalent PCR platforms in France and Cambodia. J Clin Virol. 2018 Sep 17;108:53–8.

27. putting_hiv_and_hcv_to_the_test.pdf [Internet]. [cited 2018 Oct 22]. Available from: https://www.msf.org/sites/msf.org/files/putting_hiv_and_hcv_to_the_test.pdf

28. Soulier A, Poiteau L, Rosa I, Hézode C, Roudot-Thoraval F, Pawlotsky J-M, et al. Dried Blood Spots: A Tool to Ensure Broad Access to Hepatitis C Screening, Diagnosis, and Treatment Monitoring. J Infect Dis. 2016 Apr 1;213(7):1087–95.

29. Greenman J, Roberts T, Cohn J, Messac L. Dried blood spot in the genotyping, quantification and storage of HCV RNA: a systematic literature review. J Viral Hepat. 2015 Apr;22(4):353–61.

30. Zhou K, Fitzpatrick T, Walsh N, Kim JY, Chou R, Lackey M, et al. Interventions to optimise the care continuum for chronic viral hepatitis: a systematic review and meta-analyses. Lancet Infect Dis. 2016 Sep 5;

31. Scott N, Doyle JS, Wilson DP, Wade A, Howell J, Pedrana A, et al. Reaching hepatitis C virus elimination targets requires health system interventions to enhance the care cascade. Int J Drug Policy. 2017 Aug 7;

32. Lim SG, Aghemo A, Chen P-J, Dan YY, Gane E, Gani R, et al. Management of hepatitis C virus infection in the Asia-Pacific region: an update. Lancet Gastroenterol Hepatol. 2017 Jan;2(1):52–62.

33. Pol S, Bourliere M, Lucier S, Hezode C, Dorival C, Larrey D, et al. Safety and efficacy of daclatasvir-sofosbuvir in HCV genotype 1-mono-infected patients. J Hepatol. 2017 Jan;66(1):39–47.

34. Hlaing NKT, Mitrani RA, Aung ST, Phyo WW, Serper M, Kyaw AMM, et al. Safety and efficacy of sofosbuvir-based direct-acting antiviral regimens for hepatitis C virus genotypes 1-4 and 6 in Myanmar: Real-world experience. J Viral Hepat. 2017 May 5;

35. Wehmeyer MH, Ingiliz P, Christensen S, Hueppe D, Lutz T, Simon KG, et al. Real-world effectiveness of sofosbuvir-based treatment regimens for chronic hepatitis C genotype 3 infection: results from the multicenter German hepatitis C cohort (GECCO-03). J Med Virol. 2017 Jul 15;

36. Kim DY, Han K-H, Jun B, Kim TH, Park S, Ward T, et al. Estimating the Cost-Effectiveness of One-Time Screening and Treatment for Hepatitis C in Korea. PLoS ONE. 2017;12(1):e0167770.

37. cambodia_nationalhealthplan.pdf [Internet]. [cited 2018 Oct 24]. Available from: http://www.wpro.who.int/health_services/cambodia_nationalhealthplan.pdf

38. Ozano K, Simkhada P, Thann K, Khatri R. Improving local health through community health workers in Cambodia: challenges and solutions. Hum Resour Health [Internet]. 2018 Jan 6 [cited 2018 Oct 24];16. Available from: https://www.ncbi.nlm.nih.gov/pmc/articles/PMC5756401/

39. Caille A, Kerry S, Tavernier E, Leyrat C, Eldridge S, Giraudeau B. Timeline cluster: a graphical tool to identify risk of bias in cluster randomised trials. BMJ. 2016 Aug 16;354:i4291.

21. APPENDIX

Appendix 1: Information sheet screening phase

Appendix 2: Consent form screening phase

Appendix 3: Information sheet treatment phase

Appendix 4: consent form treatment phase

Appendix 5: composition of the SAB

Appendix 6: composition of the DSMB

Appendix 7: Interview guides for acceptability study

Appendix 8: Recruitment material for the acceptability study(Information sheet and consent form of acceptability study)

Appendix1: information sheet, screening phase

Community versus facility-based services to improve the screening of active HCV infection in Cambodia: a cluster randomized controlled trial

ANRS-2384: Cam-C Study

Information sheet

Screening Phase

Version 4.0 – April 2022

……………………………

Sponsor: Inserm-**ANRS** (French National Institute for Health and Medical Research (Inserm)

ANRS Emerging Infectious Diseases – Autonomous agency of Inserm (ANRS EID), located at 2 rue Oradour-sur-Glane – 75015 Paris, France.

Coordinating investigators: Pr SAPHONN Vonthanak (University of Health Sciences) / Pr Jean-Charles DUCLOS VALLEE (Centre Hépato Biliaire, Hôpital Paul Brousse, France)

This information sheet is made to help you to take the decision to participate or not in this study.

You are free to answer "yes" or "no" to the question: do you wish to participate to this study?

You have the right to take time, to discuss about this study and to ask all the questions that you wish.

You can, if you want, be accompanied by a person of trust in the respect of the confidentiality of your pathology to help you in your decision-making.

You can also change your mind at any moment and ask to stop the participation to the study. We just ask that you inform the investigators as soon as possible.

If you do not want to participate to this study, you will continue to receive the best possible care.

GLOSSARY and DEFINITION:

- Combined-testing strategy (Antibody + RNA): Association of the 2 tests, Rapid Diagnosis Test (RDT) + HCV RNA viral load

- Coded data: the first and last names are associated with a code known only by the doctor and the persons who have access to the medical data

- Data: information collected as part of the study

- HCV: Hepatitis C Virus

- RDT: Rapid Diagnostic Test

- Right of access: right to obtain confirmation as to whether or not your personal data are being processed and where to access them if it is the case

- HCV RNA: Viral load of HCV = number of HCV in one mL of blood

- Sponsor: legal and financial manager of the study and responsible of the processing data

- WHO: World Health Organization

What is a health research study?

A health research study is a way to find out new information about a screening, a disease and /or the treatment of disease.

Why are you proposed to participate in this study?

You are aged more than 40 years old, and, so, you could eventually be infected by hepatitis C virus, as we know that the proportion of infection is high for this age group in Cambodia.

Hepatitis C infection is due to a virus (HCV), which could be responsible of liver disease in the long term. Sometimes the body gets rid of the virus and there is no liver disease. But sometimes, the virus stays in the body and the person is chronically infected. In that case, without treatment, hepatitis C infection could cause severe damage to the liver. In consequence, the liver would not be able to work properly and some people could also develop a liver cancer.

Hepatitis C infection is very common in Asia. The World Health Organization (WHO) recommends « that HCV serology testing be offered to individuals who are part of a population with high HCV seroprevalence or who have a history of HCV risk exposure/ behavior ». But WHO also mentions that « Research is needed to evaluate different approaches to increase the reach and uptake of screening services ».

For this reason, researchers developed this study to know what the best effective way is to screen HCV infection in Cambodia and identify patients with active HCV infection.

What is the aim of the study?

The purpose of this study is to compare the effectiveness of a community-based combined-testing strategy (RDT + HCV RNA) to a facility-based combined-testing strategy to improve the active case detection of HCV infection among general population aged more than 40 years old in Cambodia.

We plan to enroll 8000 participants, to screen 6000 persons and to treat 200 persons. The total duration of the study will be 12 months.

The screening phase will be divided in 2 parts:

<u>1/ HCV Antibody testing using rapid diagnosis test (RDT)</u>

The rapid test that we are proposing is made on a finger stick capillary whole blood. The result could be available in 15 minutes.

If this test is negative, you are not infected by HCV.

If this test is positive, it means that you were in contact with HCV in the past and you could be chronically infected. But you could also get rid of the virus. That's why it is necessary to perform another test called HCV RNA

2/ HCV RNA testing

If HCV RNA is negative, you get rid of the virus and you do not need any exam or treatment.

If HCV RNA is positive, you have an active infection; you need to have exams and probably a treatment.

To know, what is the best way (community-based or facility-based strategy) to perform these 2 exams, we will randomly select different groups of households to participate to one of the two following strategies:

Strategy 1/ A facility-based testing strategy. Information will be provided in your village and you will receive a voucher to go to referral health center for testing. HCV testing using HCV RDT (the first test) will be done in the health center. In case of positive HCV RDT, an immediate blood sample collection (1 tube 5 ml) will be done in health center and sent to University of Health Sciences to perform HCV RNA (the second test). Results will be sent back to the health center that will be in charge to give you the result and to refer you to Provincial Hospital for care in case of active infection (positive RDT + positive HCV RNA).

Strategy 2/ A community-based testing strategy. In that strategy, Community Health Workers (CHWs) will do the HCV RDT (the first test) on a finger stick capillary whole blood directly in your household. If you don't wish to do this test in your household, it will be possible to do the test in a specific location in the village as school or pagoda. The visits will be planned with the head of village and he will be in charge to inform you. If you are not present during the first visit, a second visit will be scheduled. In case of absence during the second visit, no additional visit will be planned.

In case of positive HCV RDT, 5 additional blood spots will be collected on a blotting paper. This technique is called Dried Blood Spot. No blood sample will be done. The blotting paper will be put in a specific plastic bag and sent to Phnom Penh (University of Health Sciences) to do the HCV RNA (the second test). Results will be sent back to the referral health center and nurse will be in charge to give you the result and to refer you to Provincial Hospital for care in case of active infection.

All persons aged more than 40 years old could be enrolled in the screening phase. All the persons living in the same group of households will use the same strategy for testing.

What does the participation to the study involve?

You have full freedom in accepting or refusing to participate in this study.

If you accept to participate in this study, you will sign the consent form that will also be signed by the heath care provider who offered you to participate in this study. Consent form will be signed in health center for facility arm and in your household for community arm.

We will collect one spot of blood on a finger stick to perform the HCV RDT. The result of this test will be provided 15 minutes after the collection of blood.

- If the result of this test is negative, it means that you don't have infection by HCV. Your counselor will explain to you preventive measures in order to avoid future infection.

- If the result is positive, it means that you could be chronically infected by HCV. In this case, we will perform the HCV RNA testing:

Strategy 1: we will collect 5 ml of blood in a tube. This tube will be sent to University of Health Sciences (Rodolphe Merieux laboratory) in Phnom Penh to perform the HCV RNA testing. Result will be available in the health center and you will be appointed to get it. The tube will be destroyed after analysis

Strategy 2: 5 spots of blood on a finger stick will be collected on a blotting paper and sent to the University of Health Sciences (Rodolphe Merieux laboratory) in Phnom Penh. When results will be available, nurse will inform you to come in the health center to get the result and counseling.

For the 2 strategies, the interpretation of the HCV RNA results is the same:

- HCV RNA is negative: it means that you get rid of the virus and you do not need any exam or treatment. Your counselor will explain to you preventive measures in order to avoid future infection.

- HCV RNA is positive: it means that you have an HCV active infection and that you need to go in consultation in the Provincial Hospital to receive care and possible treatment. All information related to this management will be provided in the Provincial Hospital with another information sheet.

All the samples collected during the screening phase will be destroyed at the end of analysis.

Cost-effectiveness analysis

In order to assess the cost and efficiency of these 2 interventions, a cost-effectiveness analysis will be conducted. We will also propose to you to answer to an interview and/or questionnaire on your socio-economical situation. You are free to refuse and, in any case, it will not affect your follow-up on the study.

What are the advantages and disadvantages of participating?

The advantage to participate to this first phase is to have the opportunity to have a free rapid screening for HCV, which is not currently available in Cambodia. If you are infected by HCV, you will be proposed a specific consultation and a possible access to HCV treatment. If you are not infected we will provide advices to avoid future infection.

The disadvantages could be associated with the blood drawing. This blood collection could possibly include lightheadedness, bleeding and/or bruising at the puncture site.

What happens if there is a problem?

In case of problem, you should report it to your health care provider or the person in charge of the study at your hospital or health center. As a research sponsor, the Inserm-ANRS purchase liability insurance for damage. This insurance guarantees compensation in case you experience any harm as the result of your involvement in this study.

Data processing and associated rights

As part of the study you may participate in, a treatment of your personal health data is implemented to analyze the results of the study with regard to the objectives.

The sponsor of the study, Inserm-ANRS, is a French research agency that fulfills a public interest mission and for scientific research purposes. This justifies the processing of personal and health data for scientific research purposes, under European and French regulations.

Your sociodemographic, clinical and biological data will be collected because they are necessary for the research. At the hospital, they are accessible to people mandated by Inserm-ANRS to

ensure the quality of research. Outside the hospital, in order to be treated confidentially, your data will be coded beforehand and then transmitted to Inserm-ANRS or to those acting on its behalf in France or abroad. Neither your name nor your first name will be associated with them.

During the research, your data and samples will be transferred for research purposes to other research teams as well as to health authorities (French or foreign). The following partner structures will be involved in the screening phase of the study: the University of Health Science in Cambodia (for the data management and statistical analysis), the provincial hospitals of Siem Reap and Kampong Cham in Cambodia (for the plasma collection of the participants in Strategy 1 with a positive HCV RDT and for the medical visits as part of the study), and the 'Rodolphe Merieux laboratory' (UHS) in Cambodia (for the HCV RNA testing of the participants in Strategy 1 and for the HCV RNA extraction and amplification of the participants in Strategy 2).

You have the right to obtain a copy of the guarantees relating to the transfer of your data.

At the end of the research, if you agree (by ticking a specific box on the consent form), your data or samples may be used and transferred to other research teams (national, international, private or public).

If this transfer happens, it will be done in order to conduct other research on Hepatitis C and associated diseases. You will be informed of the purpose of any new processing on the website https://anrs.fr

Besides, the transferred data or samples will be framed by appropriate and adapted guarantees provided in a sharing agreement under the control of Inserm-ANRS and under conditions guaranteeing the confidentiality of your data or samples. You have the right to obtain a copy of the guarantees relating to the transfer of your data.

Your rights

In accordance with the French law[1], you have a right to rectify your data collected, the right to oppose (right to opposition) or to limit their use (right to limitation of treatment). You also have the right to withdraw your consent to the research at any time without having to justify yourself. No new data will be collected afterwards. However, the data collected prior to the withdrawal of your consent cannot be erased as their erasure would compromise the achievement of the objectives of the research. They will continue to be processed under conditions that guarantee their confidentiality. National Health Authorities or the Scientific Committee who monitor this study may also decide to end it.
The rights above-mentioned are exercised with the study doctor following you as part of the research.

If you have any questions regarding these rights, you can contact your study doctor or the Data Protection Officer of the Sponsor by email (dpo@inserm.fr) or by mail (Data Protection Officer, INSERM, 101 rue de Tolbiac, 75013 Paris).

Finally, you have the right to lodge a complaint with the French supervisory authority: the CNIL (National Commission for Information Technology and Liberties, , https://www.cnil.fr/fr/cnil-direct/question/adresser-une-reclamation-plainte-la-cnil-quelles-conditions-et-comment).

[1] French law n ° 78-17 of 6 January 1978 amended in particular by Law 2018-493 of June 20, 2018, relating to Technology Data Files and Civil Liberties

Storage and archiving periods

In total, the processing of your data will last from 2022 until 2026 (time for inclusions, follow-up, analysis, etc).

Afterwards, your data will be kept for 15 years in accordance with current archiving regulations for clinical research.

Expenses and compensations

You will not pay any cost related to the HCV Rapid Test and HCV RNA.
We will not pay any transportation cost from your home to the health center.

Contact for further information

During this first phase, you can ask any questions or request additional information from your health care provider or the person in charge of the study at your hospital or health center.
It is Dr/ Mr/ Mrs…………………… (Phone: ……………………….

Appendix 2: consent form screening phase

Community versus facility-based services to improve the screening of active HCV infection in Cambodia: a cluster randomized controlled trial

ANRS 12384 study: Screening phase

Informed consent

Version 4.0 – April.2022

Approved by NECHR, Date: 25 February 2019

Sponsor: **Inserm-ANRS** (French National Institute for Health and Medical Research (Inserm)

ANRS Emerging Infectious Diseases – Autonomous agency of Inserm (ANRS EID)

Coordinating investigators: **Pr SAPHONN Vonthanak (UHS) / Pr Jean-Charles DUCLOS VALLEE (Centre Hépato Biliaire)**

I, (full name)……………………., the undersigned, declare that I have read or have been read the information sheet attached with this form and I have clearly understood the objectives, advantages and disadvantages of the screening phase.

I had the opportunity to ask all questions and discuss this research study with the health care provider who proposed me to participate, whose name and signature are shown below, as well as with his/her staff.

They answered all my questions in an understandable language. The risk and benefits have been explained to me. I believe that I have not been unduly influenced by any team member to participate in the research study by any statement or implied statements. A time for thought is possible if I wish to take time before making my decision.

I understand that I may be given, if I wish, a copy of this consent form after signing it. I understand that my participation in this screening phase is voluntary, that I may choose to refuse to participate, that, if I accept, I may withdraw my consent in any time with no penalty or disadvantage to my routine care.

I have noted that samples blood will be made during the search and kept coded. They will permit to carry out the analyzes planned for this study.

I understand that clinical and personal informations will be kept confidential. I agree that the data recorded for this research will be collected and computerized. I understand that the right of access provided by the amended law of 6 January 1978 relating to data, files and freedoms and the General Regulation on Data Protection (RGPD - Regulation (EU) 2016/679) is required at all times to the person in charge to follow me during the study and that I may exercise my right of rectification, opposition and limitation.

I accept that the scientists involved in this research as well as the persons appointed by the Sponsor, health authorities in France and abroad have access to my medical records, in respect of confidentiality

By signing this consent form, I have not waived any of the legal rights that I have as a participant in a research study.

Therefore, I fully agree to participate in this screening phase ❑Yes ❑ No

I agree to be contacted by phone or at home in relation to this screening phase. ❑Yes ❑ No

I agree that my stored data would be further used for other studies approved by the National Ethics Committee, including studies on HCV infection. ❑Yes ❑ No.

I agree to answer to questions regarding my socioeconomic situation ❑Yes ❑ No.

Participant name
signature ____________________ Date |__|__| / |__|__| / |__|__|__|__|

I, Dr., Mr, Mrs, ..., the undersigned, have fully explained the relevant details of this research study to the participant named above and believe that the participant has understood and has knowingly given his(her) consent .

I solemnly promise I will respect all the terms and conditions mentioned in this consent form, keep full confidentiality, and respect the individual's rights and freedom as well as the requirements of the scientific work.

Investigator Signature_______ ________________________ Date |__|__| / |__|__| / |__|__|__|__|

Appendix 3: Information sheet treatment phase

Community versus facility-based services to improve the screening of active HCV infection in Cambodia: a cluster randomized controlled trial

The ANRS 12384 Cam-C study

Information sheet

Treatment Phase

Version 4.0 – 04.April 2022

....

Sponsor: Inserm-**ANRS (**French National Institute for Health and Medical Research (Inserm)

ANRS Emerging Infectious Diseases – Autonomous agency of Inserm (ANRS EID), located at 2 rue Oradour-sur-Glane – 75015 Paris, France

Data controller: INSERM, 101 rue de Tolbiac, 75013 Paris

Coordinating investigators: **Pr SAPHONN Vonthanak (University of Health Sciences) / Pr Jean-Charles DUCLOS VALLEE (Centre Hépato Biliaire)**

This information sheet is made to help you to take the decision to participate or not in this study.

You are free to answer "yes" or "no" to the question: do you wish to participate to this study?

You have the right to take time, to discuss about this study and to ask all the questions that you wish.

You can, if you wish, to be accompanied by a person of confidence in the respect of the confidentiality of your pathology to help you in your decision-making to participate or not to the research and throughout this study.

You can change your mind at any moment and ask to stop the participation to the study. We just ask you to inform investigators as soon as possible.

If you don't want to participate to this study, you will continue to receive the best possible care.

GLOSSARY and DEFINITION:

- Biobank: Collection of biological samples taken from the people involved in the research (ex: blood) and prepared for their analysis and conservation.

- Coded data: the first and last names are associated with a code known only by the doctor and the persons who have access to the medical data

- Data: information collected as part of the study

- HCV: Hepatitis C Virus

- Right of access: right to obtain confirmation as to whether or not your personal data are being processed and where to access them if it is the case.

- Sponsor: legal and financial manager of the study and responsible of the processing data

What is a health research study?

A health research study is a way to find out new information about a screening, a disease and /or the treatment of disease.

Why are you proposed to participate in this second part of the study?

You are aged more than 40 years old, and, HCV active infection has been identified during the screening phase of the ANRS 12384 study.

Hepatitis C infection is due to a virus (HCV), which could be responsible of liver disease in the long term. Without treatment, hepatitis C infection could cause severe damage to your liver. In consequence, your liver would not be able to work properly and some persons could also develop a liver cancer.

For this reason, investigators propose you to be referred to the Provincial Hospital in order to assess the liver disease and to propose you a treatment for HCV infection.

What is the aim of the second part of the study?

In this second phase, we would like to evaluate the effectiveness of a consultation without delay in the Provincial Hospital to manage your HCV infection. A consultation in the Provincial Hospital will be planned and HCV treatment for 12 weeks will be proposed with a reduced liver disease assessment. In some cases detailed below, HCV treatment could not be possible in the Provincial Hospital and you will be referred to a National Hospital in Phnom Penh. We plan to treat 200 participants. The total duration of this phase is 6 months.

What does the participation to the study involve :

You have full freedom in accepting or refusing to participate in this study.

If you refuse to participate in this study, you can still benefit from the available consultation in Cambodia for HCV infection and your medical care will not be affected by your refusal.

If you accept to participate in this study, you will sign the consent form that will also be signed by the heath care provider who offered you to participate in this study.

Detailed follow-up

At inclusion visit, we will collect information about personal and familial medical history (questionnaire), perform clinical examination and use questionnaires to evaluate risk factors of HCV infection. We will collect 14 mL of blood (4 tubes) to perform biological analysis. We will also collect urine sample for pregnancy test for women of childbearing age (40-under 50 years old). A liver ultrasound will be performed in the same day.

After this first evaluation, several situations could occur:

1/ You have a severe liver disease and it will be too dangerous for you to be treated in the Provincial Hospital. You will be referred to Phnom Penh to be followed in a specific liver unit where others exams will be performed. A specific committee will review your results and take a decision for the treatment's option. Transportation and supplementary exams costs will be funded by the study.

2/ The HIV rapid test is positive and you are not aware of this possible HIV infection. In that case, we will give you the address of the closest testing center in order to confirm HIV infection.

3/ The pregnancy test is positive. In that case, the HCV treatment is not authorized and you will be addressed to antenatal care for further follow-up.

4/ You have a severe renal insufficiency. In that case, the HCV treatment is not authorized and you will be addressed to a specific consultation for further follow-up.

5/ You don't have any of the previous situations and you could be treated in the Provincial Hospital. In that case, we will propose you to start immediately a treatment for HCV infection. This treatment is composed of two different drugs that you must take every day during 12 weeks. The first drug is called sofosbuvir and you have to take one tablet of 400mg every day with food. The second drug is called daclatasvir and you have also to take one tablet of 60mg every day with food. You can take the two tablets together. During the duration of the treatment, you must come back to the Provincial Hospital every month. At each consultation, you will have a clinical exam including a report of any adverse event that could occur and we will collect 7 ml of your blood (2 tubes) for visit 4 weeks, 5 ml of your blood (1 tube) for visit 8 weeks and 10 ml of your blood (2 tubes) for visit 12 weeks. It is important to take your treatment every day at the same time to avoid forgetting. If you are a woman of childbearing age, in order to not take a risk, it will be important not to start a pregnancy during the treatment and use a contraceptive method. **It is very important to avoid any medication without the authorization of your study doctor, including traditional medicine**.

After 12 weeks, you can stop the treatment and you have to wait 12 weeks more before the next consultation. During this consultation (24 weeks), we will collect 17 ml of blood (4 tubes) to perform HCV RNA and others biological analysis.

- If HCV RNA is negative, it means that you are cured for HCV infection. In some cases, you could have to repeat some exams (explained below)
- If HCV RNA is positive, it means that you not succeed to cure the virus. It happens for 5 to 10% of cases. You will be referred to Phnom Penh in a specific liver unit where others exams will be performed including resistance testing of the drugs. A specific committee will review your results and give you a recommendation. Transportation and supplementary exams costs will be funded by the study.

End of study

Your study participation will last until week 28. Even if you are cured for HCV infection, in case of cirrhosis, you need to continue liver ultrasound follow-up every 6 months because liver cancer could occur later. This exam will not be funded by the study as the period is long and not known but you will be referred to continue follow-up.

What happens if there is a problem?

In case of a problem, you should report it to your health care provider.
As a research sponsor, the Inserm-ANRS purchase liability insurance for damage. This insurance guarantees compensation in case you experience any harm as the result of your involvement in this study including side effects of drugs.

Constitution of a biobank

Some biological samples (plasma, whole blood) will be collected at inclusion visit, 12 and 24 weeks visits, frozen, labelled with a code (without your name) and kept in the freezers of the laboratory of the Fondation Mérieux in University of Health Sciences, Phnom Penh, Cambodia and will be used in accordance with the protocol.
If your samples are not used completely at the end of this research, they will be preserved in a sustainable way to be used for further purposes than the ones directly related to this study, unless if you give an opposition. For these further utilizations, an explicit authorization will be requested from

the National Ethics Committee. You will also be informed of the purpose of any new processing on the website https://anrs.fr

If you agree that your data may be used for further purposes (specific box on the consent form), you will still be able to exercise your rights by contacting your study doctor. In case of difficulties, you can also contact the Data Protection Officer of the Sponsor by email. (dpo@inserm.fr) or by mail (Data Protection Officer, INSERM, 101 rue de Tolbiac, 75013 Paris).

All the samples will be kept frozen for a period of 15 years after your agreement.

Cost-effectiveness analysis

In order to assess the cost and efficiency of these 2 interventions, a cost-effectiveness analysis will be conducted. We will also propose you to answer to an interview and/or questionnaire on socio-economical situation. You are free to refuse and, in any case, it will affect your follow-up on the study.

What are the advantages and disadvantages of participating?

Advantages

- You will know if you are actively infected by HCV
- You will have an opportunity to be treated for your HCV infection
- You will benefit the very closely monitored medical care with addition clinical and biological examination
- You will be reimbursed for the transportation fees used to come to the hospital for your treatment follow-up (not screening phase)
- You will contribute to spread the knowledge of HCV in your country.

Possible risks and disadvantages

- You will have to come more frequently to the hospital for the purpose of the study.
- Inconvenient could be associated with the drawing of blood. That includes light-headedness, bleeding and/or bruising at the puncture site and minor infection at the vein puncture site.
- HCV drugs can produce side effects (as explained below).

During the study duration, an independent committee will assess the safety of the study (serious and non-serious adverse events) and monitor its correct implementation. If necessary, e.g. for security reasons, they may decide to discontinue the study.

HCV treatment side effects

All medicines have side effects. During the study, your doctors will be very alert to identify potential side effects and will take the necessary steps to treat them if they occur. A summary of these side effects is available in appendix of this document. We ask you to come back to see your doctor as soon as you notice anything abnormal.

During the study if your health status deteriorates and that other treatments should be preferred, or in case adverse events occur, you can decide with your study doctor to interrupt study drugs at any time while continuing follow-up in the study.

What happens when the study ends?

At the end of your participation, your physician will give your results. If the treatment doesn't succeed to cure the hepatitis C, you will be referred to Phnom Penh to perform supplementary biological and morphological exams and a specific committee will give you a therapeutic decision. All exams and transportation will be funded by the study.

Data processing and associated rights

As part of the study you may participate in, a treatment of your personal health data is implemented to analyze the results of the study with regard to the objectives.
The sponsor of the study, Inserm-ANRS, is a French research agency that fulfills a public interest mission and for scientific research purposes. Inserm-ANRS will process your personal and health data for scientific research purposes, under European and French regulations.

Your sociodemographic, clinical and biological data will be collected because they are necessary for the research. At the hospital, they are accessible to people mandated by Inserm-ANRS to ensure the quality of research. Outside the hospital, in order to be treated confidentially, your data will be coded beforehand and then transmitted to Inserm-ANRS or to those acting on its behalf in France or abroad. Neither your name nor your first name will be associated with them.

Data and samples transfer

During the research, your data and samples will be transferred for research purposes to other research teams as well as to health authorities (French or foreign). The following partner structures will be involved in the treatment phase of the study: the University of Health Science in Cambodia (for the data management and statistical analysis) and the provincial hospitals of Siem Reap and Kampong Cham in Cambodia (for the medical visits during the study).
You have the right to obtain a copy of the guarantees relating to the transfer of your data.
At the end of the research, if you agree (by ticking a specific box on the consent form), your data or samples may be used and transferred to other research teams (national, international, private or public).
If this transfer happens, it will be done in order to conduct other research on Hepatitis C and associated diseases. You will be informed of the purpose of any new processing on the website https://anrs.fr

Besides, the transferred data or samples will be framed by appropriate and adapted guarantees provided in a sharing agreement under the control of Inserm-ANRS and under conditions guaranteeing the confidentiality of your data or samples.

You have the right to obtain a copy of the guarantees relating to the transfer of your data.

Your rights

In accordance with the French law[2] and the General Data Protection Regulation[2], you have a right to rectify your data collected, the right to oppose (right to opposition) or to limit their use (right to limitation of treatment). You have the right to withdraw your consent to research at any time without having to justify yourself. No new data will be collected afterwards. However, the data collected prior to the withdrawal of your consent cannot be erased as their erasure would compromise the achievement of the objectives of the research. They will continue to be processed under conditions that guarantee their confidentiality. National Health Authorities or the Scientific Committee who monitor this study may also decide to end it.

---

[2] French law n ° 78-17 of 6 January 1978 amended in particular by Law 2018-493 of June 20, 2018, relating to Technology Data Files and Civil Liberties

[4] Regulation (EU) 2016/679 of the European Parliament and of the Council of 27 April 2016 on the protection of natural persons with regard to the processing of personal data and on the free movement of such data (General Data Protection Regulation)

If you have any questions regarding these rights, you can contact your study doctor or the Data Protection Officer of the Sponsor by email (dpo@inserm.fr) or by mail (Data Protection Officer, INSERM, 101 rue de Tolbiac, 75013 Paris).

Finally, you have the right to lodge a complaint with the French supervisory authority: the CNIL (National Commission for Information Technology and Liberties, , https://www.cnil.fr/fr/cnil-direct/question/adresser-une-reclamation-plainte-la-cnil-quelles-conditions-et-comment).

Storage and archiving periods

In total, the processing of your data will last from 2022 until 2026 (time for inclusions, follow-up, analysis, etc).

Afterwards, your data will be kept for 15 years in accordance with current archiving regulations for clinical research.

Expenses and compensations

You will not pay any costs related to this study: the costs of the biological and radiological diagnosis, of hospitalization and transportation expenses for protocol visits will be covered by the study during the entire duration of your follow-up.
Your participation is entirely voluntary and you will not receive any financial compensation.

Contact for further information
During the study period, you can ask any questions or request additional information from your health care provider or the person in charge of the study at your hospital or Health Centre.

Dr/ Mr/ Mrs ……………………   Phone number: ……………………

Appendix : Summary side effects

Possible adverse effects of medication

- All medicines can cause side effects, although not everybody gets them.
- In order to determine if what you are feeling is due to a medication, you should report any new symptoms to your doctor.
- In the event of severe symptoms or an allergic skin reaction with fever, itching, fatigue, swelling of the face or lips, shortness of breath, muscle or joint pain, you should consult your doctor as soon as possible or go to the hospital emergency unit.
- Some side effects can happen straight away while others may take weeks or months.

HCV treatment

Daclatasvir and Sofosbuvir have common main side effects including :

- Headaches
- Fatigue
- Nausea
- Musculoskeletal pain
- Heart rhythm disorder, in association with medicinal products that lower heart rate
- If you have ever had hepatitis B virus infection, the hepatitis B virus could become active again during or after treatment of hepatitis C virus with these treatments. Hepatitis B virus becoming active again (called reactivation) may cause serious liver problems. Your healthcare provider will monitor you if you are at risk for hepatitis B virus reactivation during treatment and after you stop taking these treatments.

Sofosbuvir is often used in combination, some of the side effects listed below may be observed :

- Abnormal blood test results
- Loss of appetite
- Mood swings, anxiety, difficulty sleeping
- Abnormal liver test

For women of childbearing age

Administered this medicine during pregnancy could be toxic to the developing unborn child

You should consider the use of adequate contraception to prevent pregnancy and continue its use for at least 5 weeks after the last administration.

If you are pregnant, think you may be pregnant or are planning to have a baby, ask your doctor for advice about taking this medicine.

The doctor who follows you as part of this study remains at your disposal to answer all your questions.

Appendix 4: consent form therapeutic phase

**Community versus facility-based services to improve the screening of active HCV infection in Cambodia: a cluster randomized controlled trial**<br>**ANRS 12384 Study : Treatment phase**

Informed consent

Version 3.0 05 March 2021

Sponsor: **Inserm- ANRS** (Institut National de la Santé et de la Recherche Médicale; France REcherche Nord & sud Sida-hiv Hépatites)

Coordinating investigators: **Pr SAPHONN Vonthanak (UHS) / Pr Jean-Charles DUCLOS VALLEE (Centre Hépato Biliaire)**

I, (full name)……………………., the undersigned, declare that I have read or have been read the information sheet attached with this form and I have clearly understood the objectives, advantages and disadvantages of the treatment phase.

I had the opportunity to ask all questions and discuss this research study with the health care provider who proposed me to participate, whose name and signature are shown below, as well as with his/her staff.

They answered all my questions in an understandable language. The risk and benefits have been explained to me. I believe that I have not been unduly influenced by any team member to participate in the research study by any statement or implied statements. A time for thought is possible if I wish to take time before making my decision.

I understand that I may be given, if I wish, a copy of this consent form after signing it. I understand that my participation in this screening phase is voluntary, that I may choose to refuse to participate, that, if I accept, I may withdraw my consent in any time with no penalty or disadvantage to my routine care.

I have noted that samples of plasma and blood will be made during the search and kept coded. They will permit to carry out the analyzes planned for this study.

I understand that clinical and personal information will be kept confidential. I agree that the data recorded for this research will be collected and computerized. I understand that the right of access provided by the amended law of 6 January 1978 relating to data, files and freedoms and the General Regulation on Data Protection (RGPD - Regulation (EU) 2016/679) is required at all times to the person in charge to follow me during the study and that I may exercise my right of rectification, opposition and limitation.

I accept that the scientists involved in this study as well as the persons appointed by the Sponsor, health authorities in France and abroad have access to my medical records by the team in charge of the study, in respect of confidentiality.

By signing this consent form, I have not waived any of the legal rights that I have as a participant in a research study.

Therefore, I fully agree to participate in this treatment phase ❑**Yes** ❑ **No**

I agree to be contacted by phone or at home in relation to this treatment phase. ❑**Yes** ❑ **No**

I agree that my stored samples and data would be further used for other studies approved by the National Ethics Committee, including studies on HCV infection. **Yes** **No.**

I agree to answer to questions regarding my socioeconomic situation **Yes** **No.**

Participant name
signature ___________________ Date |__|__| / |__|__| / |__|__|__|__|

I, Dr., Mr, Mrs, ... the undersigned, have fully explained the relevant details of this research study to the participant named above and believe that the participant has understood and has knowingly given his(her) consent .

I solemnly promise I will respect all the terms and conditions mentioned in this consent form, keep full confidentiality, and respect the individual's rights and freedom as well as the requirements of the scientific work.

Investigator Signature_______ _______________________ Date |__|__| / |__|__| / |__|__|__|__|

Appendix 5 : composition of the SAB

anrs France Recherche Nord & sud Sida-hiv Hépatites
Agence autonome de l'Inserm

List of members- Scientific committee of the ANRS 12384 study
Version N° 1 30/01/2019

Study title : Community versus facility based services to improve active case detection of HCV infection in Cambodia : a cluster randomized controlled trial

Coordinating data and methodology center : UHS Phnom Penh Cambodia
Name of the president of the Scientific committee : Olivier Ségéral

Name Surname	Field of competence	Institution	Voting member	Contact
**Project team**				
Pr SAPHONN Vonthanak	South Coordinating investigator	UHS	Yes	[illegible]onthanak@[illegible]hs.edu.kh
Pr Jean-Charles DUCLOS VALLEE	North Coordinating investigator	CHU Paul Brousse	Yes	jean-charles.duclos-vallee@aphp.fr
Dr NETH Sansothy	Clinical coordinator	UHS	Yes	[illegible]nsothy@[illegible]uhs.edu.kh
Dr Olivier SEGERAL	Clinical coordinator	ANRS/UHS	Yes	oliseg@hotmail.com
Dr BY Youlet	Virologist	Fondation Merieux/UHS	Yes	youlet.by@fondation-merieux.org
Dr Eric NERIENNET	Virologist	Fondation Merieux	Yes	enerrienet@gmail.com
Dr SUY Sovanthida	Statistician	UHS	Yes	sovanthidasuy@uhs.edu.kh
Dr MAM Sovatha	Data manager	UHS	Yes	sovatha@uhs.edu.kh
Pr Laurence MEYER	Methodology	INSERM SC10-US019, Villejuif	Yes	laurence.meyer@inserm.fr
Dr Luis SAGAON TEYSSIER	Cost effectiveness	UMR912 SESSTIM/ORS PACA, Marseille	Yes	luis.sagaon-teyssier@inserm.fr
**External experts**				
Dr TEK Bunchhoeung	CDCD	MoH	Yes	bunchhoeung@yahoo.com
Pr Bruno GIRAUDEAU	Methodology	CHU Tours	Yes	bruno.giraudeau@univ-tours.fr
Dr Francois-Xavier BABIN	Director of Health System and Diagnostic	Fondation Merieux	Yes	fx.babin@fondation-merieux.org
**Sponsor**				
Dr Laure-Amélie MONTEYNARD	Project Manager	International Research and Collaboration unit ANRS	Yes	laure-amelie.monteynard@anrs.fr
Dr Isabelle Fournier	Senior Project Manager	International Research and Collaboration unit ANRS	No	Isabelle.fournier@anrs.fr
Dr Lena [illegible]	Vigilance	ANRS	Yes	lena.[illegible]@anrs.fr
Dr Alpha Diallo	Vigilance – Unit Head	ANRS	No	alpha[illegible]@anrs.fr

Comments :

....

Date : Le 05/02/2019

Signature of the ANRS director :

Page 1/1

Appendix 6: composition of the DSMB

France REcherche Nord & sud Sida-hiv Hépatites

Agence autonome de l'Inserm

Appendix 2

LIST OF DMC MEMBERS
DATA MONITORING COMMITTEE FOR ANRS STUDY 12384 CAM-C

Version 01 of 18/10/2019

Study Title: Community versus facility-based services to improve the screening of active HCV infection in Cambodia : a cluster randomized controlled trial

SURNAME First name	Field of competencies	Institution
TUAILLON Edouard	Virology	*CHU de Montpellier, France*
SOGNI Philippe	Hepatitis	*Hôpital Cochin, France*
LARMARANGE Joseph	Methodology	*IRD-CEPED, France*

Name of the Data Monitoring Committee Chair (to be defined during the 1st meeting – cf. Meeting opinion of that meeting).

Comments :

....
....
....
....
....
....

Date : October 21, 2019

Signature of ANRS Director

Agence Nationale de Recherches sur le Sida et les Hépatites Virales - ANRS -

Page 1/1

Appendix 7 : Interview guide for the acceptability study

- **Interview guide for head of health centers and head of operational districts**
- **Focus group guide for people who accepted and do not accept to be tested for HCV in the CAM-C study**

Note: This is an inductive qualitative approach. Researchers gain an in-depth comprehension of the subject matter through empirical evidence. Data collection and analysis occur concurrently. With emerging patterns, it could influence participant selection and data collection tools.

Although the interviews will be semi-structured, the structure is open to modification: from interviews that are included, some questions might be rearranged and new questions might be added, and some might be discarded. We will inform the Ethics committee if the interview guide needed to be significantly altered.

INTERVIEW GUIDE

Head of health centers and head of operational districts

Introduction

- Welcome participants, introduce yourself
- Explain the purpose of the interview and the issues to be addressed.
- Invite participants to introduce him/herself and briefly share their experience with CAM-C study and the HCV testing procedure.

Interview guide

- Can you briefly describe your role?
- How do you involve with HCV testing and care cascade provided in the CAM-C?
- In your opinion, how effective the community-based/the facility-based HCV testing in improving testing uptake?
- How would you describe the current HCV testing in Cambodia?
- How do existing HCV testing in Cambodia affect HCV testing uptake?
- In your opinion, what are the strengths and weaknesses of community-based/facility-based HCV testing strategy?
- Can you discuss any recent changes after implementing community-based/facility-based HCV testing intervention?

- How do you think the community-based/facility-based HCV testing strategy can be improved to better address the HCV screening in Cambodia?
- How would you evaluate the sustainability of these testing strategies in Cambodia, particularly in relation to HCV testing uptake?
- What are your expectations for the future development of testing strategies in Cambodia, particularly to improve HCV testing uptake?
- How can Cambodia ensure that HCV testing strategies remain responsive to the evolving needs of its population, particularly in relation to HCV prevention, treatment, and control?

FOCUS GROUP DISSCUSION GUIDE FOR THE ACCEPTABILITY STUDY

People who accepted to be tested for HCV in the CAM-C study

&

People who do not accept to be tested for HCV in the CAM-C study

Introduction

- Welcome participants, introduce the facilitator and the note-taker.
- Explain the purpose of the focus group discussion and the issues to be addressed.
- Establish, in cooperation with the participants, the rules for respectful and inclusive communication.
- Invite participants to introduce themselves and briefly share their experience with the HCV testing particularly the CAM-C project.

The understanding of HCV and HCV screening

- In your words, what is HCV? (Are there things that people do to know their HCV status? If so, which things?)
- How can someone tell if they have HCV?
- What people should do if they want to know their HCV status?
- How did you learn about the HCV testing intervention?
- Did you get HCV tested? Did you participate in one of the CAM-C intervention strategies? What are the reasons you chose (not) to do so?
- What benefits and services are provided by the intervention?
- Did the HCV testing intervention impact your health-seeking behavior? If so, how?
- What are the strengths of the HCV testing intervention you know? (Use examples)
- What are the limitations of the HCV testing intervention you know? (Use examples)
- Do you think the HCV testing interventions have made it easier for people to get HCV screening?
- How well do you think the HCV testing interventions help with preventing or treating HCV?
- From your experiences, how good are the healthcare services for HCV screening in Cambodia? Can you give some examples?
- How well do you think doctors and nurses communicate to patients about how to screen for HCV? Are there any ways you think they could do better?

- Have you had any problems getting HCV screening in Cambodia? What do you think can be done to make this better?
- What are some ways you think these strategies can be made better to help with HCV screening?
- How much do you think people in Cambodia know about HCV screening? What can be done to help people know more and understand the benefits of these testing/screening strategies?
- Ask participants if they have anything else to add about HCV screening in Cambodia.
- Thank participants for the willingness to take part in the research

Appendix 8: RECRUITMENT MATERIALS FOR THE ACCEPTABILITY STUDY

Community versus facility-based services to improve the screening of active HCV infection in Cambodia: Acceptability study

ANRS 12384 study

Script to introduce the study information and to recruit participants

Sponsor: **Inserm-ANRS** (Institut National de la Santé et de la Recherche Médicale; France REcherche Nord & sud Sida-hiv Hépatites)

Coordinating investigators: **Pr SAPHONN Vonthanak (UHS) / Pr Jean-Charles DUCLOS VALLEE (Centre Hépato Biliaire)**

Dear [Participant's Name],

My name is ___________________, and I am a researcher at the University of Health Sciences (UHS) in Phnom Penh. Our university is conducting a research project about acceptability of HCV testing/screening in Cambodia.

As part of the study, we are conducting qualitative research to understand the experiences, perspectives, and challenges faced by beneficiaries, and health care providers (including head of social health centers, head of ODs, doctors, nurses, and VHSG). Additionally, we are also collecting data from people who accepted and not accepted to be tested for HCV in each of the interventions of the CAM-C study

We believe that your insights and experiences could greatly contribute to our research. Therefore, we would like to invite you to participate in an [interview/focus group discussion] as part of this study. The [interview/focus group] is expected to take approximately [60-90 minutes for interview/90-120 minutes for focus group] and will be held at a location convenient for you. Your participation is entirely voluntary, and you may withdraw from the study at any time without any negative consequences. Each participant will receive a gift maximum 15$ as compensation for your time spent with us.

To ensure confidentiality and protect your privacy, we will not use your name or any identifiable information in our research reports. The data collected will be anonymized.

If you are interested in participating or would like more information about the study, please feel free to contact our researchers listed below. Our researchers would be more than happy to answer any questions you may have and provide additional details about the research.

We greatly appreciate your time and consideration, and we hope you will consider participating in this important study. Your experiences and perspectives could make a valuable contribution to HCV testing/screening uptake.

Thank you.

Sincerely,

Dr. Neth San sothy
University of Health Sciences
Phnom Penh, Cambodia
nssothy@uhs.edu.kh

Dr. Dyna Khuon
University of Health Sciences
Phnom Penh, Cambodia
khuondyna@uhs.edu.kh

CONSENT MATERIALS FOR THE ACCEPTABILITY STUDY

- **Informed consent form – Interviews**
- **Informed consent form – Focus groups**

Community versus facility-based services to improve the screening of active HCV infection in Cambodia: Acceptability study

ANRS 12384 study

INFORMED CONSENT FORM - INTERVIEWS

Sponsor: **Inserm-ANRS** (Institut National de la Santé et de la Recherche Médicale; France Recherche Nord & sud Sida-hiv Hépatites)

Coordinating investigators: **Pr SAPHONN Vonthanak (UHS) / Pr Jean-Charles DUCLOS VALLEE (Centre Hépato Biliaire)**

Purpose of the Study: This study aims to explore the acceptability of HCV testing among the population toward facility-based testing intervention and community-based testing intervention. It aims to collect data from people who decide to have HCV tested and not to have HCV tested. It also collects data from individuals involved in the implementation of the intervention, such as the head of the health center, head of ODs, doctors, nurses, community workers, and VHSG.

Procedures: By participating in this study, you will be asked to engage in an interview lasting approximately 60-90 minutes. The interview will be conducted at a location convenient for you. The session will be audio-recorded to ensure accuracy in data analysis.

Voluntary participation: Your participation in this study is completely voluntary. You may withdraw from the study at any time without any negative consequences and without the need to justify the withdrawal.

Confidentiality: Your confidentiality will be protected throughout the study. We will not use your name or any identifiable information in our research reports. All data collected will be anonymized.

Compensation: As a token of appreciation for your time and effort, you will receive a gift maximum 15$ as compensation for participating in the study.

Risks and Benefits: The risks involved in participating in this study are minimal. However, discussing your experiences may evoke emotions or memories that may be uncomfortable. If you feel uncomfortable at any point during the study, you have the right to skip the questions that are uncomfortable, ask for a break, or stop your participation.

The study does not provide any direct benefits to participants. However, the information provided may inform strategies for improving the HCV testing/screening uptake in order to better management and control of HCV in Cambodia.

Questions: If you have any questions or concerns about this study, please feel free to contact the principal investigator.

Consent: Your signature below indicates that you have read and understood the information provided above, have had the opportunity to ask questions and have them answered, and agree to participate in the study.

Participant name
signature ____________________ Date |__|__| / |__|__| / |__|__|__|__|

Investigator Signature__________________________ Date |__|__| / |__|__| / |__|__|__|__|

Community versus facility-based services to improve the screening of active HCV infection in Cambodia: Acceptability study

ANRS 12384 study

INFORMED CONSENT FORM – FOCUS GROUP

Sponsor : **Inserm-ANRS** (Institut National de la Santé et de la Recherche Médicale; France REcherche Nord & sud Sida-hiv Hépatites)

Coordinating investigators: **Pr SAPHONN Vonthanak (UHS) / Pr Jean-Charles DUCLOS VALLEE (Centre Hépato Biliaire)**

Purpose of the Study: This study aims to explore the acceptability of HCV testing among the population toward facility-based testing intervention and community-based testing intervention. It aims to collect data from people who decide to have HCV tested and not to have HCV tested. It also collects data from individuals involved in the implementation of the intervention, such as the head of the health center, head of ODs, doctors, nurses, community workers, and VHSG.

Procedures: By participating in this study, you will be asked to engage in a focus group discussion lasting approximately 90-120 minutes. The focus group will be conducted at a location convenient for you. The session will be audio-recorded to ensure accuracy in data analysis.

Voluntary participation: Your participation in this study is completely voluntary. You may withdraw from the study at any time without any negative consequences and without the need to justify the withdrawal.

Confidentiality: Your confidentiality will be protected throughout the study. We will not use your name or any identifiable information in our research reports. All data collected will be anonymized.

Compensation: As a token of appreciation for your time and effort, you will receive a gift maximum 15$ as compensation for time in participating in the study.

Risks and Benefits: The risks involved in participating in this study are minimal. However, discussing your experiences may evoke emotions or memories that may be uncomfortable. If

you feel uncomfortable at any point during the study, you have the right to skip the questions that are uncomfortable, ask for a break, or stop your participation.

The study does not provide any direct benefits to participants. However, the information provided may inform strategies for improving the HCV testing/screening uptake in order to better management and control HCV in Cambodia.

Questions: If you have any questions or concerns about this study, please feel free to contact the principal investigators.

Consent: Your signature below indicates that you have read and understood the information provided above, have had the opportunity to ask questions and have them answered, and agree to participate in the study.

Participant name
signature ____________________ Date |__|__| / |__|__| / |__|__|__|__|

Investigator Signature_________________ Date |__|__| / |__|__| / |__|__|__|__|
