# Supplementary material for: Community-based versus facility-based services to improve hepatitis C screening in Cambodia: a cluster randomized controlled trial (ANRS 12384 Cam-C study)
Source: Lancet Reg Health West Pac. 2025 Oct 8;63:101703. doi: 10.1016/j.lanwpc.2025.101703 (PMC12538039; doi:10.1016/j.lanwpc.2025.101703)
Supplement: Supplementary File–Tables [file mmc2.docx]

**Supplementary file 1. Sensitivity analysis: Inverse probability weighting (IPW) to correct differences between the facility-based and community-based arms**

**Table S1. Weighted Baseline characteristics according to strategy (i.e., study arm) at the individual level**

| **Characteristics** | **Facility-based strategy (n=7692)** | **Community-based strategy (n=7692)** | **p-value** | **Total** |
| --- | --- | --- | --- | --- |
|  | **% or Median[IQR]** | **% or Median[IQR]** |  | **% or Median[IQR]** |
| **Age (years)** | 56 [47-65] | 56 [47-65] |  | 55 [47-65] |
| **Gender** |  |  |  |  |
| Men | 42.1 | 42.1 | 0.974 | 42.2 |
| Women | 57.8 | 57.9 |  | 57.8 |
| **Marital status** |  |  |  |  |
| Single | 2.9 | 2,9 | 0.999 | 2.9 |
| Married/cohabitating | 77.0 | 76.9 |  | 77.0 |
| Widowed/divorced/separated | 20.1 | 20.1 |  | 20.1 |
| **Activity workload** |  |  |  |  |
| No activity | 64.0 | 64.2 | 0.985 | 64.1 |
| Work ≤7 days per week and <8h per day | 17.0 | 17.0 |  | 17.0 |
| Work 7 days per week and ≥ 8h per day | 19.0 | 18.8 |  | 18.9 |
| **Other household member(s) previously diagnosed with and treated for HCV HCV^a^** |  |  |  |  |
| Yes | 1.3 | 1.3 | 0.941 | 1.3 |
| No | 98.7 | 98.7 |  | 98.7 |
| **Pre-study history of HCV testing (participant)^b^** |  |  |  |  |
| Yes | 3.2 | 3.2 | 0.967 | 3.2 |
| No | 96.8 | 96.8 |  | 96.8 |
| **Perceived health status at the time of the interview^c^** |  |  |  |  |
| Good/Very good | 64.4 | 64.5 | 0.972 | 64.5 |
| Very poor/poor/moderate | 35.6 | 35.5 |  | 35.5 |
| **Province** |  |  |  |  |
| Kampong Cham | 56.6 | 56.7 | 0.926 | 56.7 |
| Siem Reap | 43.4 | 43.3 |  | 43.3 |
| ^a^ 31 missing values; ^b^ 32 missing values; ^c^ 26 missing values | |  |  |  |

**Table S2. Weighted Characteristics according to study strategy (i.e., study arm) at the individual level for participants who had an HCV RDT (N= 5590)**

| **Characteristics** | **Facility-based strategy (n=5590)** | **Community-based strategy (n=5590)** | **p-value** | **Total** |
| --- | --- | --- | --- | --- |
|  | **%(n) or Median[IQR]** | **%(n) or Median[IQR]** |  | **%(n) or Median[IQR]** |
| **Age (years)** | 56 [47-65] | 56 [47-65] | 0.047 | 56 [47-65] |
| **Gender** |  |  |  |  |
| Men | 1065 (41.0) | 1187 (39.7) | 0.337 | 2252 (40.3) |
| Women | 1535 (59.0) | 3338 (60.3) |  | 3338 (59.7) |
| **Marital status** |  |  |  |  |
| Single | 65 (2.5) | 83 (2.8) | 0.257 | 148 (2.7) |
| Married/cohabitating | 2018 (77.6) | 2265 (75.7) |  | 4283 (76.6) |
| Widowed/divorced/separated | 517 (19.9) | 642 (21.5) |  | 1159 (20.7) |
| **Activity workload** |  |  |  |  |
| No activity | 1724 (66.3) | 2158 (72.2) | <0.001 | 3882 (69.5) |
| Work ≤7 days per week and <8h per day | 397 (15.3) | 416 (13.9) |  | 813 (14.5) |
| Work 7 days per week and ≥ 8h per day | 479 (18.4) | 416 (13.9) |  | 895 (16.0) |
| **Other household member(s) previously treated for HCV** |  |  |  |  |
| Yes | 4 (0.2) | 15 (0.5) | 0.026 | 19 (0.3) |
| No | 2596 (99.8) | 2975 (99.5) |  | 5571 (99.7) |
| **Pre-study history of HCV testing (participant)** |  |  |  |  |
| Yes | 19 (0.7) | 39 (1.3) | 0.035 | 58 (1.0) |
| No | 2581 (99.3) | 2951 (98.7) |  | 5532 (99.0) |
| **Perceived health status at the time of the interview** |  |  |  |  |
| Good/Very good | 1667 (64.1) | 2033 (68.0) | 0.002 | 3700 (66.2) |
| Very poor/Poor/Moderate | 933 (35.9) | 957 (32.0) |  | 1890 (33.8) |
| **Province** |  |  |  |  |
| Kampong Cham | 1145 (44.0) | 1733 (58.0) | <0.001 | 2878 (51.5) |
| Siem Reap | 1455 (56.0) | 1257 (42.0) |  | 2712 (48.5) |
| ^a^ One participant refused to provide a blood sample for an HCV RNA test |  |  |  |  |

**Table S3. Weighted Mixed effects model to assess the effect of the community-based strategy (i.e., study arm) with respect to the facility-based strategy (i.e., study arm) on combined testing uptake (Intention-to-treat and per protocol estimations)**

|  | **Model 0** | | | **Model 1*** | | |
| --- | --- | --- | --- | --- | --- | --- |
|  | **OR** | **95%CI** | **p-value** | **aOR** | **95%CI** | **p-value** |
| ***Fixed effects*** |  |  |  |  |  |  |
| **Strategy** |  |  |  |  |  |  |
| Community-based (ref. Facility-based) | 2.29 | [1.28 – 4.13] | 0.005 | 2.30 | [1.29 – 4.10] | 0.005 |
| **Age** (continuous, years) |  |  |  | 1.11 | [1.05 – 1.18] | <0.001 |
| **Age²** (non-linear effect) |  |  |  | 0.999 | [0.998 – 0.9994] | <0.001 |
| **Gender**: Women (ref. Men) |  |  |  | 1.16 | [1.01 – 1.33] | 0.031 |
| **Marital status** (ref. single) |  |  |  |  |  |  |
| Married/cohabitating |  |  |  | 1.99 | [1.38 – 2.87] | <0.001 |
| Widowed/divorced/separated |  |  |  | 1.95 | [1.33 – 2.86] | 0.001 |
| **Activity workload** (ref. No activity) |  |  |  |  |  |  |
| Work ≤7 days per week and <8h per day |  |  |  | 0.30 | [0.16 – 0.57] | <0.001 |
| Work 7 days per week and ≥ 8h per day |  |  |  | 0.24 | [0.14 – 0.42] | <0.001 |
| **Other household member(s) previously diagnosed with and treated for HCV** |  |  |  |  |  |  |
| Yes (ref. No) |  |  |  | 0.25 | [0.07 – 0.87] | 0.030 |
| **Pre-study history of HCV testing (participant)** |  |  |  |  |  |  |
| Yes (ref. No) |  |  |  | 0.12 | [0.06 – 0.26] | <0.001 |
| **Perceived health status at the time of the survey** |  |  |  |  |  |  |
| Very poor/Poor/Moderate (ref. good/ very good) |  |  |  | 0.36 | [0.17 – 0.78] | 0.009 |
| **Province**: Siem Reap (ref. Kampong Cham) |  |  |  | 3.17 | [1.70 – 5.90] | <0.001 |
| **Random effects:**  **80 villages / 160 clusters** | **Coeff.** | **95%CI** | | **Coeff.** | **95%CI** | |
| **σ^2^_clusters_** | 0.33 | [0.21 – 0.52] | | 0.40 | [0.26 – 0.63] | |
| ICC-clusters | 0.35 | [0.27 – 0.45] | | 0.35 | [0.27 – 0.45] | |
| **σ^2^_villages_** | 1.47 | [0.93 – 2.32] | | 1.39 | [0.86 – 2.26] | |
| ICC-villages | 0.29 | [0.21 – 0.39] | | 0.27 | [0.19 – 0.38] | |

*****60 missing values resulted in a sample of N=7632: the combination of the variables ‘other household members previously diagnosed with and treated for HCV’, ‘pre-study history of HCV testing (participant)’, and ‘perceived health status at the time of the survey’.

**Table S4. Weighted mixed effects model to assess the effect of the community-based strategy with respect to the facility-based strategy on secondary outcomes**

|  | **Confirmation testing uptake  (HCV RDT & HCV RNA)  n=5590** | | | **Active HCV infection detection  (positive HCV RDT & HCV RNA) n=5590** | | | **Linkage to care***  **n=93** | | |
| --- | --- | --- | --- | --- | --- | --- | --- | --- | --- |
|  | **aOR^a^** | **95%CI** | **p-value** | **aOR^a^** | **95%CI** | **p-value** | **aOR^b^** | **95%CI** | **p-value** |
| ***Fixed effects*** |  |  |  |  |  |  |  |  |  |
| **Strategy** |  |  |  |  |  |  |  |  |  |
| Community-based (ref. Facility-based) | 0.60 | [0.31 - 1.19] | 0.145 | 0.76 | [0.36 – 1.59] | 0.461 | 0.39 | [0.34 – 4.56] | 0.457 |
|  |  |  |  |  |  |  |  |  |  |
| **Random effects: 80 villages / 160 clusters** | **Coeff.** | **95%CI** | | **Coeff.** | **95%CI** | |  |  |  |
| **σ^2^_clusters_** | 2.02 | [0.89 – 4.60] | | 0.18 | [0.003 – 10.61] | |  |  |  |
| ICC-clusters | 0.42 | [0.29 – 0.55] | | 0.48 | [0.33 – 0.63] | |  |  |  |
| **σ^2^_villages_** | 0.35 | [0.01 – 0.67] | | 0.25 | [0.01 – 42.8] | |  |  |  |
| ICC-villages | 0.06 | [0.002 – 0.67] | | 0.04 | [0.0001 – 0.906] | |  |  |  |
| *Mixed effects model was not better than single-level logistic model (LR-test p=0.259). In addition, the IPW technique has not been applied given the small sample; **^a^** adjusted for age, age², gender, marital status, activity workload, other household member(s) previously diagnosed with and treated for HCV, pre-study history of HCV testing (participant), and perceived health status; **^b^** adjusted for age, age² and gender. | | | | | | | | | |
